# Evolutionarily diverged on-switch for actin assembly in fungal endocytosis

**DOI:** 10.1083/jcb.202506135

**Published:** 2026-10-28

**Authors:** Juliette Sebellin, Fabina Binth Kandiyoth, V.T. Bagyashree, Andrea Picco, Nicolas Carvalho, Markku Hakala, Anne-Sophie Rivier-Cordey, Alphée Michelot, Christopher P. Toret, Marko Kaksonen

**Affiliations:** 1 Department of Biochemistry, University of Geneva, Geneva, Switzerland; 2 Aix Marseille University, CNRS, IBDM, Turing Centre for Living Systems, Marseille, France; 3 MIDA.science, Geneva, Switzerland; 4 CNRS International Research Laboratory Bridging Mechanobiology and Medicine (IRL 3639 BMC), Mechanobiology Institute, National University of Singapore (NUS), Singapore

## Abstract

Clathrin-mediated endocytosis is a conserved eukaryotic trafficking process where an Arp2/3 complex–nucleated branched actin network provides force for vesicle formation. The mechanisms that initiate endocytic actin assembly are incompletely understood. In the fission yeast, *Schizosaccharomyces pombe*, actin assembly is initiated by Dip1, an Arp2/3 complex activator. In the budding yeast, *Saccharomyces cerevisiae,* the initiation of actin assembly has remained a mystery. Here, we show that *S. cerevisiae* Ldb17, the homolog of Dip1, functions as an on-switch for endocytic actin assembly. Unexpectedly, the regulation of Ldb17 is more complicated than that of constitutively active Dip1. Ldb17 is controlled by a coat protein, Sla1, via separate recruitment and activation steps. This regulation was likely lost in the *S. pombe* lineage, and this simplification may be related to other changes in actin assembly between these species. Our findings add a key missing piece in the understanding of endocytosis in *S. cerevisiae* and reveal an intriguing evolutionary tinkering of the actin on-switch.

## Teaser

Branched actin networks provide force for endocytic vesicle formation, yet what triggers their assembly in budding yeast has remained unknown. Sebellin et al. identify Ldb17 as the endocytic actin on-switch, controlled by the coat protein Sla1 through separate recruitment and activation steps, a regulation lost in fission yeast.

## Introduction

Clathrin-mediated endocytosis is a conserved eukaryotic process in which cells internalize plasma membrane vesicles for intracellular trafficking to regulate nutrient uptake, cell signaling, and membrane protein and lipid homeostasis (McMahon and Boucrot, 2011). Most clathrin-mediated endocytic proteins are conserved in fungi and animals (Dergai et al., 2016), and assemble sequentially at endocytic sites to temporally mediate endocytic event initiation, membrane bending, and vesicle release (Kaksonen and Roux, 2018). However, the mechanisms of membrane bending and vesicle production have diverged between species. How this variation is achieved with largely conserved machinery remains unknown (Kaksonen and Roux, 2018). Even between fungi, *Saccharomyces cerevisiae*, *Schizosaccharomyces pombe*, and *Ustilago maydis,* a surprising endocytic variation occurs in protein amounts, sequential assemblies, and invagination and vesicle budding kinetics (Picco et al., 2025; Sun et al., 2019).

In fungi, actin is essential for membrane bending and vesicle formation, and actin assembly is precisely timed (∼10 s) during the final endocytic steps (Picco et al., 2025; Sun et al., 2019). Specifically, the major endocytic Arp2/3 complex activators, WASP and class I myosin motors, assemble around the endocytic coat. The coat contains Pan1, another Arp2/3 complex activator, and together these proteins stimulate Arp2/3 complex–dependent branched actin network assembly (Picco et al., 2015; Sun et al., 2006). These branched actin networks are anchored to the membrane and to the endocytic coat via Sla2, Ent1, and Ent2, clathrin-binding adaptor proteins. Actin network assembly at the plasma membrane propels the linked coat and associated membrane inward, thereby driving vesicle budding (Kaksonen et al., 2003; Skruzny et al., 2012). In *S. cerevisiae* and *U. maydis,* WASP arrives 15–20 s before actin assembly (Picco et al., 2025; Sun et al., 2015). The pre-assembly of WASP may facilitate an isotropic actin network assembly around the coat (Mund et al., 2018). However, in *S. pombe* WASP co-assembles with actin (Sirotkin et al., 2005; Sirotkin et al., 2010; Picco et al., 2025). This shift in actin regulation and endocytic assembly divergence is significant given the central role of actin in endocytosis, but how the actin assembly timing is controlled is unknown.

In *S. pombe*, Dip1 is a proposed trigger for actin assembly at endocytic sites (Basu and Chang, 2011). WISH, Dip1, and Spin90 (WDS) proteins are an ortholog family that share a conserved structured core containing an Arp2/3 complex–binding region (Wagner et al., 2013). *S*. *pombe* Dip1 and mammalian Spin90 have a conserved function in actin filament nucleation (Cao et al., 2020; Kim et al., 2006a; Wagner et al., 2013). Unlike other types of Arp2/3 complex activators, WDS proteins are able to nucleate a new unbranched actin filament by activating the Arp2/3 complex independent of a preformed actin filament. Dip1 activates the Arp2/3 complex on a different face than WASP and does not depend on a WCA domain (Balzer et al., 2018; Wagner et al., 2013). The newly formed filament could provide the first filament for formation of an actin branch via WASP-dependent activation of the Arp2/3 complex at endocytic sites (Balzer et al., 2020). In the *S. pombe* endocytic pathway, WASP, Dip1, and actin all assemble at similar times and Dip1 may provide the initial switch that triggers WASP-activated branch assembly (Basu and Chang, 2011). WASP and Dip1 synergize their activities *in vitro*, which implies a potent cooperation for endocytic branched actin assembly (Balzer et al., 2020). The Dip1 homolog in *S. cerevisiae*, Ldb17, localizes to endocytic sites just prior to actin assembly, and mutants have endocytosis and actin patch defects (Burston et al., 2009). However, it has not been studied in detail in the budding yeast endocytic pathway and nothing is known about the *U. maydis* ortholog.

The literature suggests a mechanistic role of Ldb17 and Dip1 in endocytic actin network nucleation. The *S. cerevisiae* and *U. maydis* versus the *S. pombe* endocytic pathway assemblies differ in this critical moment of actin assembly. We sought to investigate WDS protein function at fungal endocytic sites to understand how Dip1 mechanisms relate to the *S. cerevisiae* pathway and uncover mechanistic divergences.

## Results

### Fungal WDS proteins colocalize with late coat proteins and show actin-dependent dynamics

**Figure 1.**
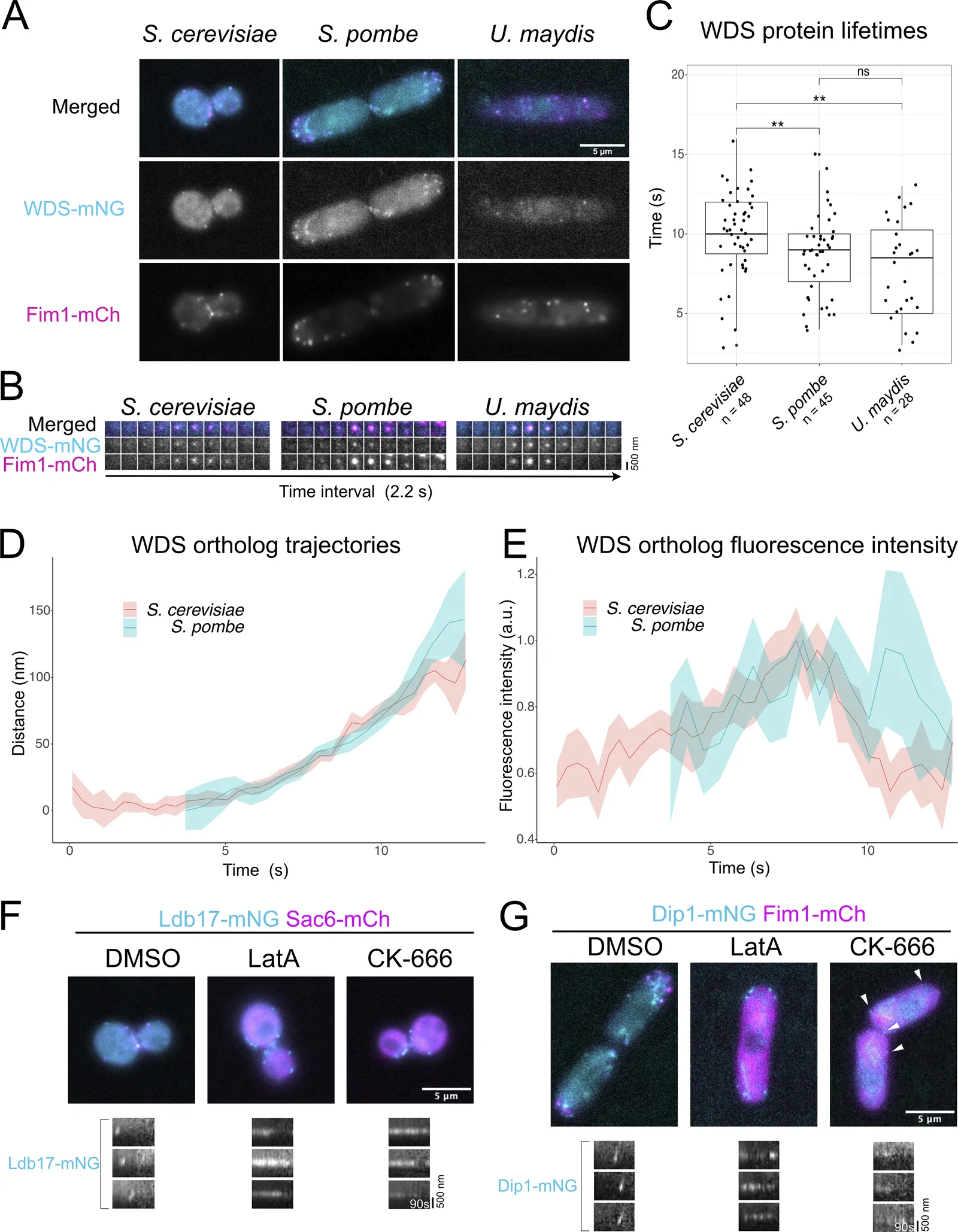
**Dikarya WDS protein localizations. (A)** Equatorial epifluorescence images for WDS and Fim1 orthologs in each species. **(B)** Two-color patch montages from TIRF microscopy movies that show indicated WDS ortholog (cyan) and co-expressed Fim1 ortholog (magenta) in each of the three species. **(C)** Boxplot showing WDS ortholog lifetime. *n* is the number of patches taken from 5 to 10 cells. ** = P ≤ 0.01. **(D)** Trajectory plot of WDS orthologs in *S. cerevisiae* (red) and *S. pombe* (blue). Error is the 95% confidence interval. Approximately 30 trajectories were acquired for each species and aligned and averaged as previously described (Picco and Kaksonen, 2017). The two species were aligned to maximize overlap of average inward movements. **(E)** Fluorescence intensity profile of WDS orthologs in *S. cerevisiae* (red) and *S. pombe* (blue). Error is the 95% confidence interval. **(F)** Equatorial epifluorescence images of Ldb17 (cyan) and Sac6 (magenta) treated with DMSO, LatA, and CK-666. Associated kymographs are displayed below. **(G)** Equatorial epifluorescence images of Dip1 (cyan) and Fim1 (magenta) treated with DMSO, LatA, and CK-666. Associated kymographs are displayed below. Arrows in white.

We wanted to compare the assembly dynamics and motile behavior of WDS family proteins in three species of Dikarya within a phylogenetic context: two ascomycete species, *S. cerevisiae* and *S. pombe,* and a basidiomycete species, *U. maydis*. In these species, the endocytic assembly pathways are quantitatively described, differ significantly in assembly timing, and provide an evolutionary context to understand directional changes in endocytic mechanisms (Picco et al., 2025). We tagged the endogenous WDS ortholog in each species C-terminally with mNeonGreen (mNG) in strains that also expressed an endogenous fimbrin ortholog tagged with mCherry (mCh). Fimbrin marks the actin filaments at endocytic sites and provides a temporal landmark for endocytic actin-dependent vesicle formation stage (Kaksonen et al., 2005; Sirotkin et al., 2010). The cells were imaged at the midplane with live epifluorescence microscopy. In all three species, the WDS family proteins localized to cortical patches that colocalized with fimbrin patches (Fig. 1 A and Video 1). Specifically, 98% of *S. cerevisiae* (*n* = 99 patches/7 cells), 96.7% of *S. pombe* (*n* = 92 patches/5 cells), and 93.4% of *U. maydis* (*n* = 76 patches/4 cells) fimbrin ortholog patches were colocalizing with a WDS protein patch. This observation suggested that the endocytic role of the WDS proteins is broadly conserved in Dikarya. WDS proteins are expressed at low levels in all three species. The WDS-GFP signals are close to the level of cellular autofluorescence, which resulted in detection of cellular autofluorescent structures unrelated to WDS proteins. This was previously observed in *S. pombe* (Balzer et al., 2019). Therefore, we used TIRF microscopy to minimize photobleaching and cytoplasmic background to analyze the timing of WDS protein localization at endocytic sites. In *S. cerevisiae*, Ldb17 arrived <5 s prior to actin assembly at endocytic sites, which is consistent with earlier observations (Burston et al., 2009). In comparison, *S. pombe* (Dip1) and *U. maydis* (UMAG_01405, henceforth called Dip1) orthologs appeared just before fimbrin (Fig. 1 B), in agreement with earlier observations, which showed that *S. pombe* Dip1 and Wsp1 arrive ∼1 s before actin assembly (Basu and Chang, 2011). We found that Ldb17 had a slightly longer lifetime (∼10 s) than the other two WDS proteins, ∼9 s (*S. pombe* and *U. maydis*) (Fig. 1 C), which is consistent with the recruitment differences between species. These results suggest a similar arrival for Dikarya WDS proteins just prior to actin assembly, which is consistent with an actin trigger function.

**Video 1.** **Movies for Figure 1.** Movie order and labelings correspond to the indicated related main figure panels. Equatorial epifluorescence image sequences for WDS (cyan) and Fim1 (magenta) orthologs. (Top) Indicated species, (middle) *S. cerevisiae* with indicated actin poison, and (bottom) *S. pombe.* The time interval between frames is 2.2 s. Scale bars are 2 µm. Time indicated in the timestamp applies to all individual panels.

Since the WASP assembly mechanisms diverged in *S. cerevisiae* and *S. pombe* (Picco et al., 2025), we focused on these species. We imaged Ldb17 and Dip1 patches at equatorial planes in *S. cerevisiae* and in *S. pombe*, respectively, and then applied particle tracking of the endocytic patches. Particle tracking provides information about the relative position and movement of endocytic proteins during vesicle formation. Both orthologs internalized at the end of the patch lifetime at similar speeds for ∼100–150 nm (Fig. 1 D). Ldb17 fluorescence intensity peaked at 7 s before falling precipitously. Dip1 showed a similar pattern of assembly and disassembly, but the signal was noisier due to a weaker signal at endocytic sites in *S. pombe*, likely due to a comparably lower number of molecules (Fig. 1 E). The motile behaviors of Ldb17 and Dip1 proteins are consistent with endocytic coat proteins that internalize at a similar initial rate in *S. pombe* and *S. cerevisiae* (Kaksonen et al., 2005; Sirotkin et al., 2010). This motile behavior would also be consistent with the finding that Dip1 stays bound to the activated Arp2/3 complex (Balzer et al., 2019). Therefore, Dip1, and possibly Ldb17, could move inward together with the assembling actin filament network at a similar rate as the coat proteins that are also propelled by the growing actin network.

Both Dip1 and Ldb17 localize independently of actin (Basu and Chang, 2011; Burston et al., 2009). To further compare the assembly dynamics of these proteins, we inhibited actin assembly with latrunculin A (LatA) or CK-666. LatA sequesters actin monomers and depolymerizes actin filaments (Ayscough et al., 1997), whereas CK-666 is an Arp2/3 complex inhibitor (Nolen et al., 2009). In the LatA-treated *S. cerevisiae* cells, persistent Ldb17 patches flickered at the membrane for the duration of the movie (2.5 min) and remained immotile (Fig. 1 F and Video 1). Dip1 patches similarly flickered and remained immotile in LatA-treated *S. pombe* cells (Fig. 1 G and Video 1). In the presence of CK-666, Ldb17 and Dip1 were both recruited to persistent, nonmotile patches that flickered similar to LatA (Fig. 1, F and G). However, unlike Ldb17, Dip1 recruitment was weaker in the presence of CK-666 relative to LatA (Fig. 1 G, white arrowheads), which suggests the Arp2/3 complex inhibition has a stronger effect on Dip1 behavior. Together, these results indicate that WDS proteins are recruited independently of actin, while Dip1 has an additional sensitivity for Arp2/3 complex function, which hints at a potential difference between orthologs. Moreover, actin dynamics are essential for normal WDS protein movement and disassembly at endocytic sites, which suggested a largely shared relationship with the actin cytoskeleton.

### Dip1 and Ldb17: Similar mutant phenotypes but different *in vitro* behaviors

**Figure 2.**
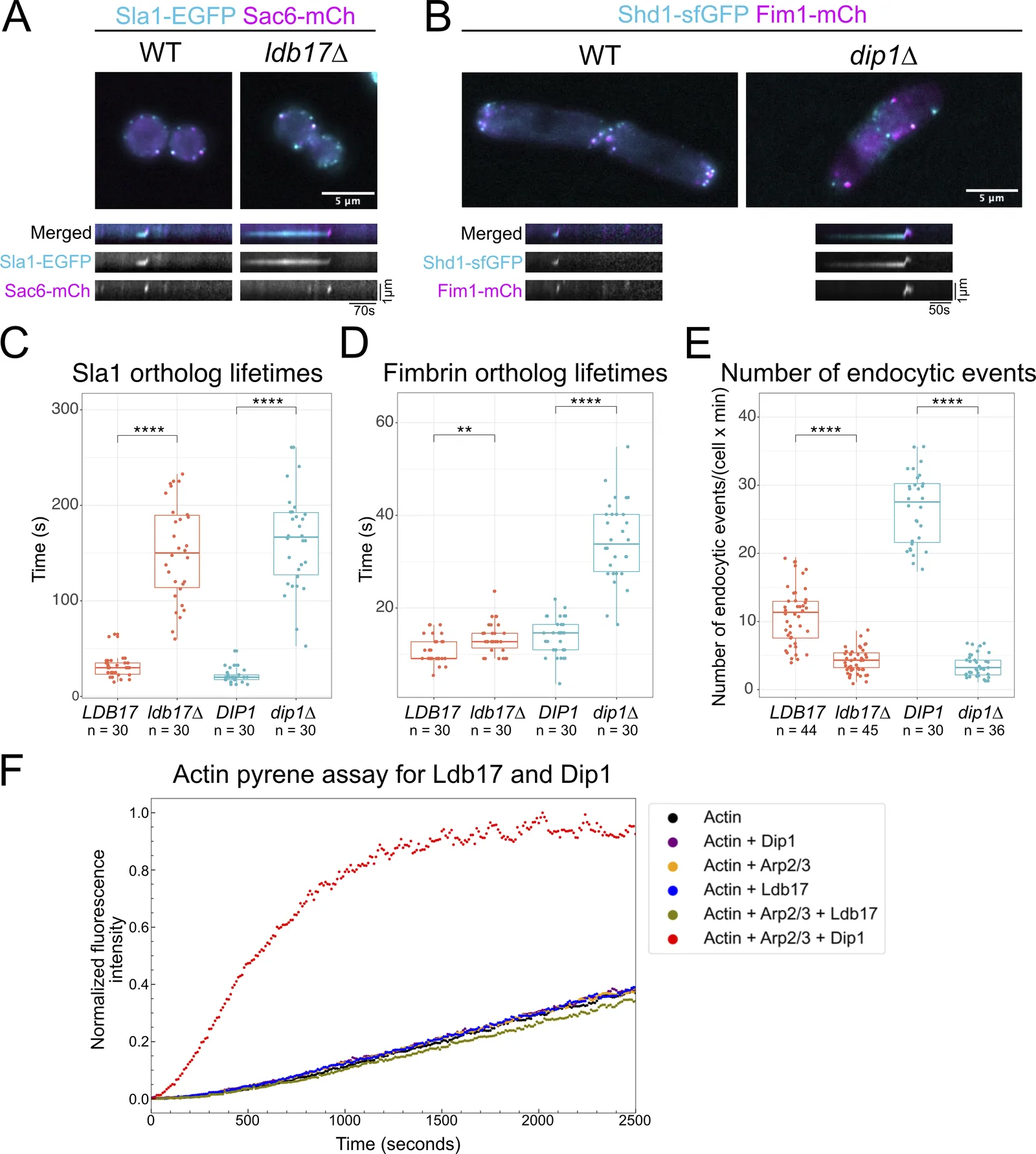
**Comparison of WDS protein functions in *S. cerevisiae* and *S. pombe*. (A)** Equatorial epifluorescence images of *S. cerevisiae* for the coat marker Sla1-EGFP (cyan) and the actin marker Sac6-mCh (magenta) in WT and Ldb17 knockout. Associated kymographs are displayed below. **(B)** Equatorial epifluorescence images of *S. pombe* for the coat marker Shd1-EGFP (cyan) and the actin marker Fim1-mCh (magenta) in WT and Dip1 knockout. Associated kymographs are displayed below. **(C)** Boxplot of coat lifetime in *S. cerevisiae* and *S. pombe* using Sla1-EGFP or Shd1-EGFP in the context of *ldb17Δ*, *dip1Δ*, and their respective WT. *n* is the number of patches taken from 5 to 10 cells. **** = P ≤ 0.0001. **(D)** Boxplot of actin lifetime in *S. cerevisiae* and *S. pombe* using Sac6-mCh or Fim1-mCh in the context of *ldb17Δ*, *dip1Δ*, and their respective WT. *n* is the number of patches taken from 5 to 10 cells. ** = P ≤ 0.01 and **** = P ≤ 0.0001. **(E)** Number of endocytic events per cell per minute in the context of *ldb17Δ*, *dip1Δ*, and their respective WT. *n* is the number of events measured from 5 to 10 cells. **** = P ≤ 0.0001. **(F)** Time course of actin polymerization monitored by pyrene fluorescence. The protein concentrations are 2 µM for actin, 0.1 µM for Arp2/3 complex, and 1 µM for all the other proteins.

We next compared WDS protein function in the two species by gene deletion. Previously, *S. pombe dip1* deletion was shown to delay the initiation of actin assembly at endocytic sites (Basu and Chang, 2011). We imaged endocytic proteins Sla1-EGFP as a marker for the vesicle coat and Sac6-mCh as a marker for endocytic actin in *S. cerevisiae* and their respective orthologs in *S. pombe*, Shd1-sfGFP and Fim1-mCh. In wild-type (WT) *S. cerevisiae* cells, Sla1 first appeared at the plasma membrane as immotile patches and then internalized with actin assembly (Fig. 2 A and Video 2) as previously described (Kaksonen et al., 2003). In *ldb17* null cells, Sla1-EGFP patches still internalized with the arrival of actin similar to WT (Fig. 2 A). However, a marked difference occurred in the early immotile phase, which was dramatically extended (Fig. 2 A, kymographs). In *S. pombe,* Dip1 deletion resulted in a similar extension of the immotile phase of Shd1-sfGFP patches prior to actin-associated internalization (Fig. 2 B). In addition, in *S. pombe,* Dip1 mutant cells were shorter and rounder than WT cells, while no obvious cell shape change was seen in *S. cerevisiae* (Fig. 2, A and B). These results suggest a conserved role of the WDS proteins in regulating endocytosis in these two species.

**Video 2.** **Movies for Figures 2, 5, and 6.** Movie order and labelings correspond to the indicated related main figure panels. Equatorial epifluorescence image sequences. (Top) *S. cerevisiae* with Sla1-EGFP (cyan) and Sac6-mCh (magenta) in WT and ldb17Δ cells. (Upper middle) *S. pombe* with Shd1-EGFP (cyan) and Fim1-mCh (magenta) in WT and dip1Δ cells. (Lower middle) *S. cerevisiae* with Pan1-EGFP (cyan) and Sac6-mCh (magenta) in WT, ldb17Δ, sla1Δ, and ldb17Δ sla1Δ cells. (Bottom) *S. cerevisiae* with Las17-EGFP (cyan) and Sac6-mCh (magenta) in WT, ldb17Δ, sla1Δ, and ldb17Δ sla1Δ cells. The time interval between frames is 2.2 s. Scale bars are 2 µm. Time indicated in the timestamp applies to all individual panels.

The lifetimes for Sla1 patches were significantly extended and more variable in both *ldb17Δ* and *dip1Δ* mutants relative to the WT cells (Fig. 2 C). Fimbrin patch lifetimes were also extended in the mutants in both species, but a more dramatic effect occurred in *S. pombe* (Fig. 2 D). We then counted the number of Sla1-EGFP patches that internalized per cell within 1 min, which corresponds to the rate of actin patch initiation. This analysis showed a significant reduction in the frequency of endocytic events in the WDS protein mutants (Fig. 2 E). These results reveal a similar delay for both species in actin assembly at endocytic sites in WDS protein mutants.

**Figure S1.**
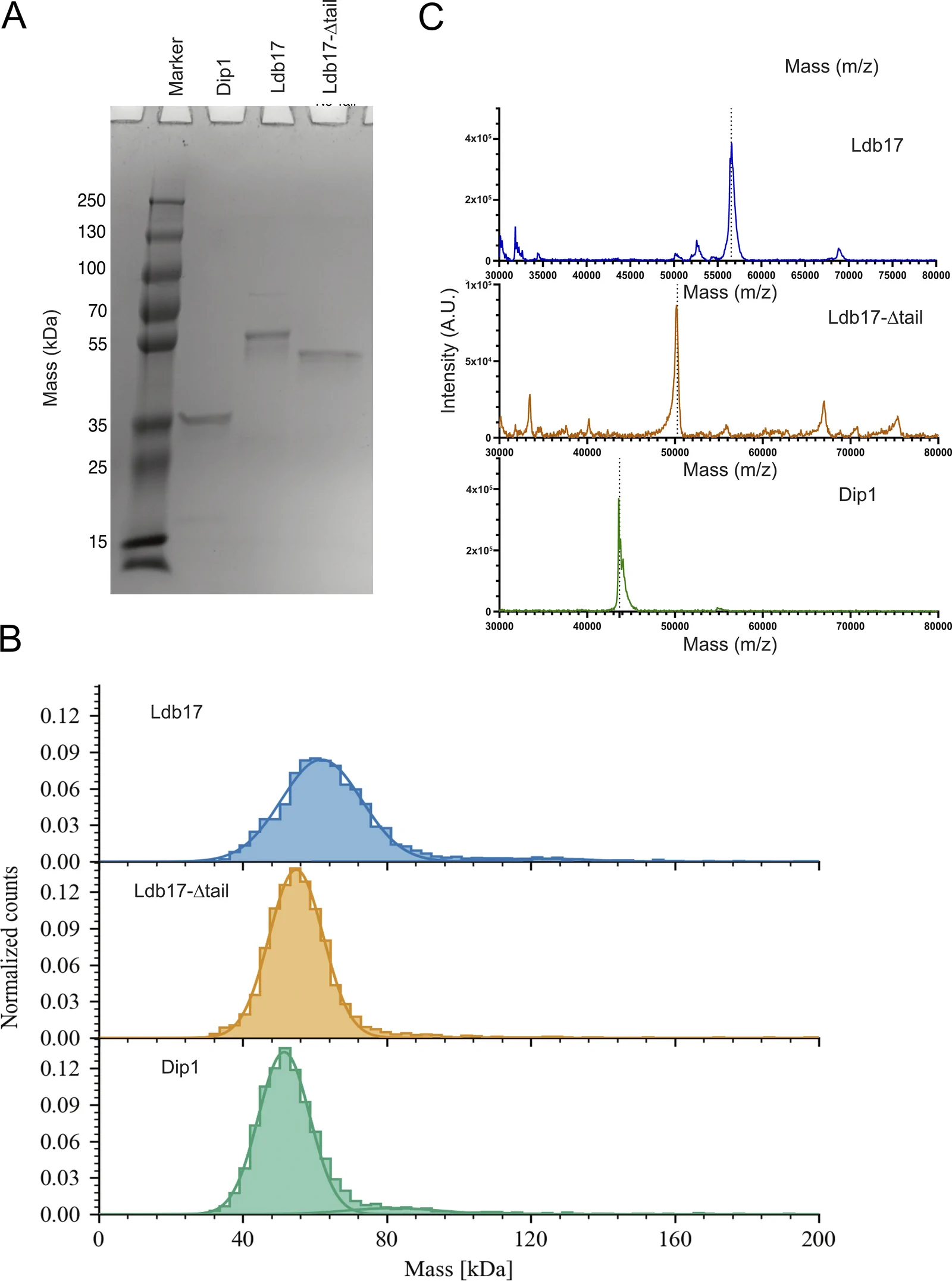
**Mass and oligomerization analysis of Dip1, Ldb17, and Ldb17-Δtail. (A)** SDS-PAGE of Dip1, Ldb17, and Ldb17-Δtail following purification. **(Band C)** mass photometry profiles and (C) MALDI spectra of Ldb17 (blue), Ldb17-Δtail (orange), and Dip1 (green). In B, the dotted vertical bars represent the theoretical masses of the proteins. Source data are available for this figure: SourceData FS1.

Based on the similarity of the deletion phenotypes, we hypothesized that *S. cerevisiae* Ldb17 functions similar to *S. pombe* Dip1 by directly activating the Arp2/3 complex to generate the first actin filaments at the endocytic site (Wagner et al., 2013). To test this idea, we expressed and purified both Ldb17 and Dip1 to compare their Arp2/3 complex activation activity by a pyrene actin assay. Both Dip1 and Ldb17 were monomeric and monodisperse (Fig. S1). The addition of Ldb17 or Dip1 with actin alone resulted in no measurable effect on actin assembly (Fig. 2 F). Dip1 resulted in strong activation of Arp2/3 complex–mediated actin polymerization (Fig. 2 F), which is consistent with earlier results that used Dip1 with *S. pombe* Arp2/3 complex proteins (Balzer et al., 2020; Wagner et al., 2013). In contrast, Ldb17 had little to no effect on actin polymerization rate when added to actin and the Arp2/3 complex (Fig. 2 F). This contrasting result suggests that despite conserved localizations and deletion phenotypes, there is a striking difference in Arp2/3 complex activation abilities between Ldb17 and Dip1. We speculated that Ldb17 may require an additional factor for its activation in contrast to Dip1.

### Conserved Arp2/3 complex–binding surface of Ldb17 is required *in vivo*

**Figure 3.**
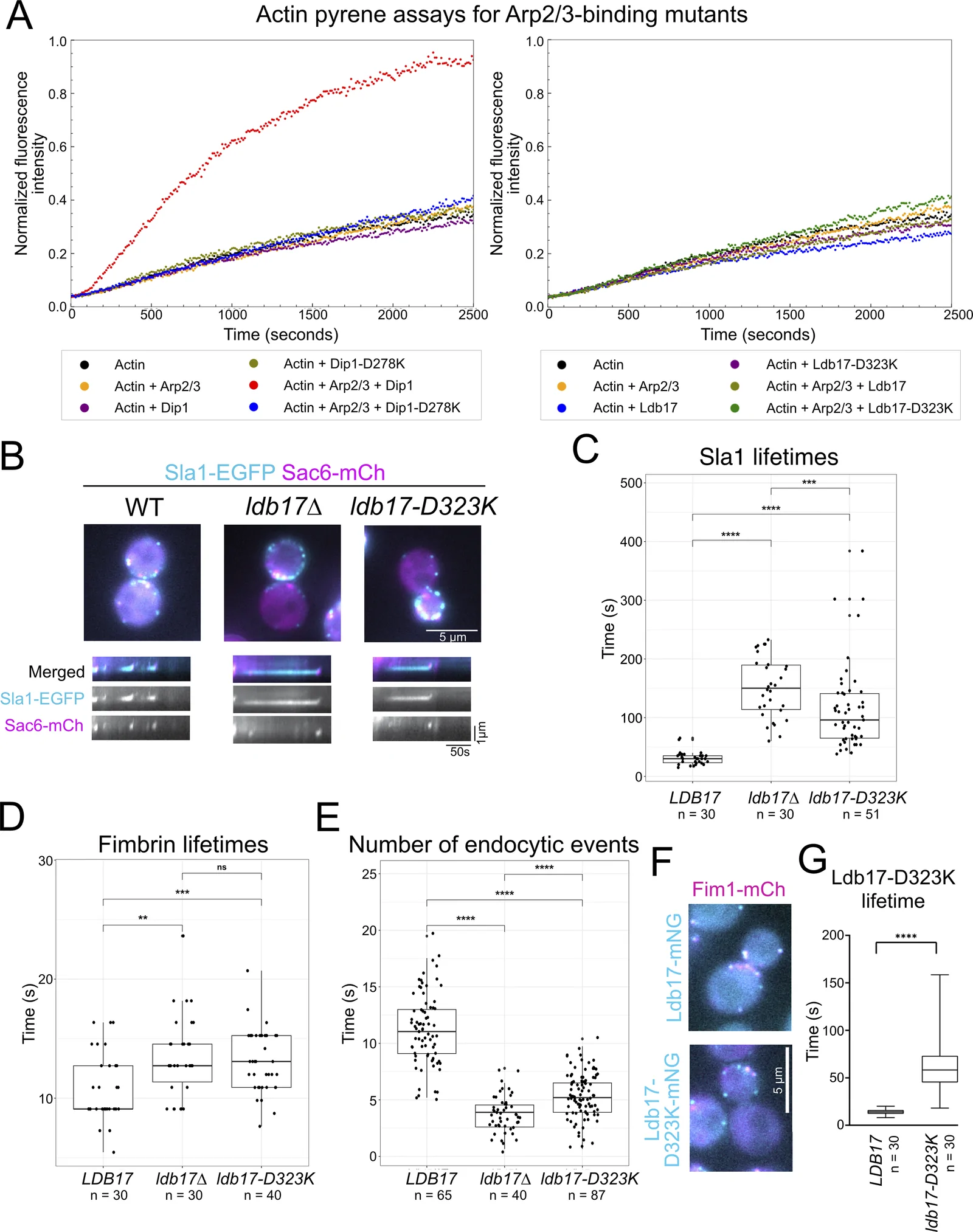
**Effects of WDS protein Arp2/3-binding mutants. (A)** Time course of actin polymerization monitored by pyrene fluorescence. The protein concentrations are 2 µM for actin, 0.1 µM for Arp2/3 complex, and 1 µM for all the other proteins. **(B)** Equatorial epifluorescence images of *S. cerevisiae* for the coat marker Sla1-EGFP (cyan) and the actin marker Sac6-mCh (magenta) in WT and *ldb17* mutants. Associated kymographs are displayed below. **(C)** Boxplot of coat lifetime using Sla1-EGFP in the indicated cells. *n* is the number of patches taken from 5 to 10 cells. *** = P ≤ 0.001 and **** = P ≤ 0.0001. **(D)** Boxplot of Sac6-mCh lifetimes in the indicated cells. *n* is the number of patches taken from 5 to 10 cells. ** = P ≤ 0.01 and *** = P ≤ 0.001. **(E)** Number of endocytic events per cell per minute in the indicated cells. *n* is the number of events measured from 5 to 10 cells. **** = P ≤ 0.0001. **(F)** Equatorial epifluorescence images for mNG-tagged Ldb17 and Ldb17-D323K and with Sac6 in each species. **(G)** Boxplot of Ldb17 lifetimes for WT and *ldb17-D323K* cells. *n* is the number of patches taken from 5 to 10 cells. **** = P ≤ 0.0001.

The *in vitro* assay suggested that Ldb17 may not function via the Arp2/3 complex. To test this, we generated point mutants in the conserved region, which was demonstrated to bind to the Arp2/3 complex (Shaaban et al., 2020). A charge inversion mutation of the conserved Dip1 residue D278K was shown to strongly inhibit Arp2/3 complex activation (Liu et al., 2022). We generated this Dip1 mutation and the homologous Ldb17 mutation, Ldb17-D323K. Pyrene assays revealed that Dip1-D278K abolished Arp2/3 complex activation relative to WT Dip1 as previously reported (Fig. 3 A). As expected, no effect of Ldb17-D323K was observed in the pyrene assay relative to the inactive WT Ldb17 (Fig. 3 A). We next tested the functionality of the *ldb17-D323K* mutant *in vivo*. Sla1-GFP and Sac6-mCh localized to patches in the *ldb17-D323K* mutant cells similar to WT and *ldb17Δ* cells (Fig. 3 B). However, Sla1 in *ldb17-D323K* cells had extended lifetimes similar to *ldb17Δ* cells (Fig. 3 C). Patch lifetimes and endocytic event quantifications revealed that the *ldb17-D323K* phenotype was similar to, although less severe than, that of the null mutant (Fig. 3, C–E). To test whether the mutant Ldb17 was localized normally in the cell, we analyzed Ldb17-D323K-mNG Sac6-mCh cells. Ldb17-D323K-mNG localized to patches comparable to WT Ldb17 (Fig. 3 F). Ldb17-D323K-mNG patches had an extended, variable lifetime (Fig. 3 G), which suggests that the point mutant Ldb17 localizes normally but is functionally impaired at endocytic sites leading to delayed initiation of actin assembly. Together, these data suggest that despite Ldb17 lacking an apparent biochemical activity *in vitro*, the Arp2/3 complex binding of Ldb17 is likely central to its endocytic function.

### Sequence comparison suggests evolution in the regulation of WDS proteins

**Figure 4.**
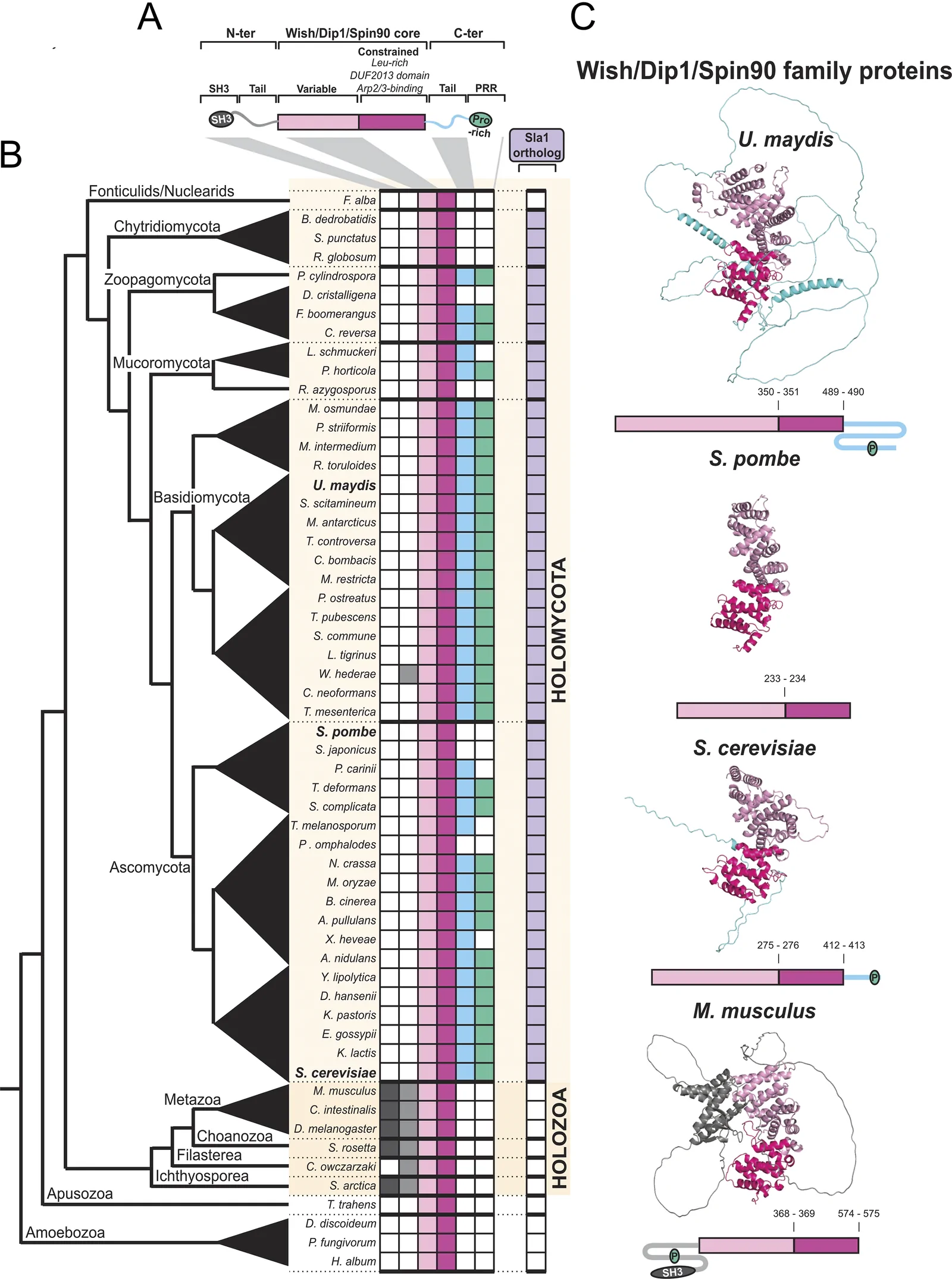
**WDS protein family. (A)** Top schematic shows generalized WDS ortholog organization. For each species, a dot indicates the presence of N-terminal tail, N-terminal SH3 domain, variable core, constrained core, C-terminal PRR, or C-terminal tail. **(B)** Selected WDS orthologs in Amorphea. Species and accession numbers are indicated and are organized based on the relative position on the tree of life (left). Boxes indicate the presence of regions depicted in A. The rightmost column indicates the presence of a Sla1 ortholog in the species. **(C)** AlphaFold structures of WDS orthologs of indicated species. WDS core regions variable are shown in light pink and constrained in magenta, and C-terminal tails are in cyan. All ortholog cores were aligned and oriented in the same direction in PyMOL.

**Figure S2.**
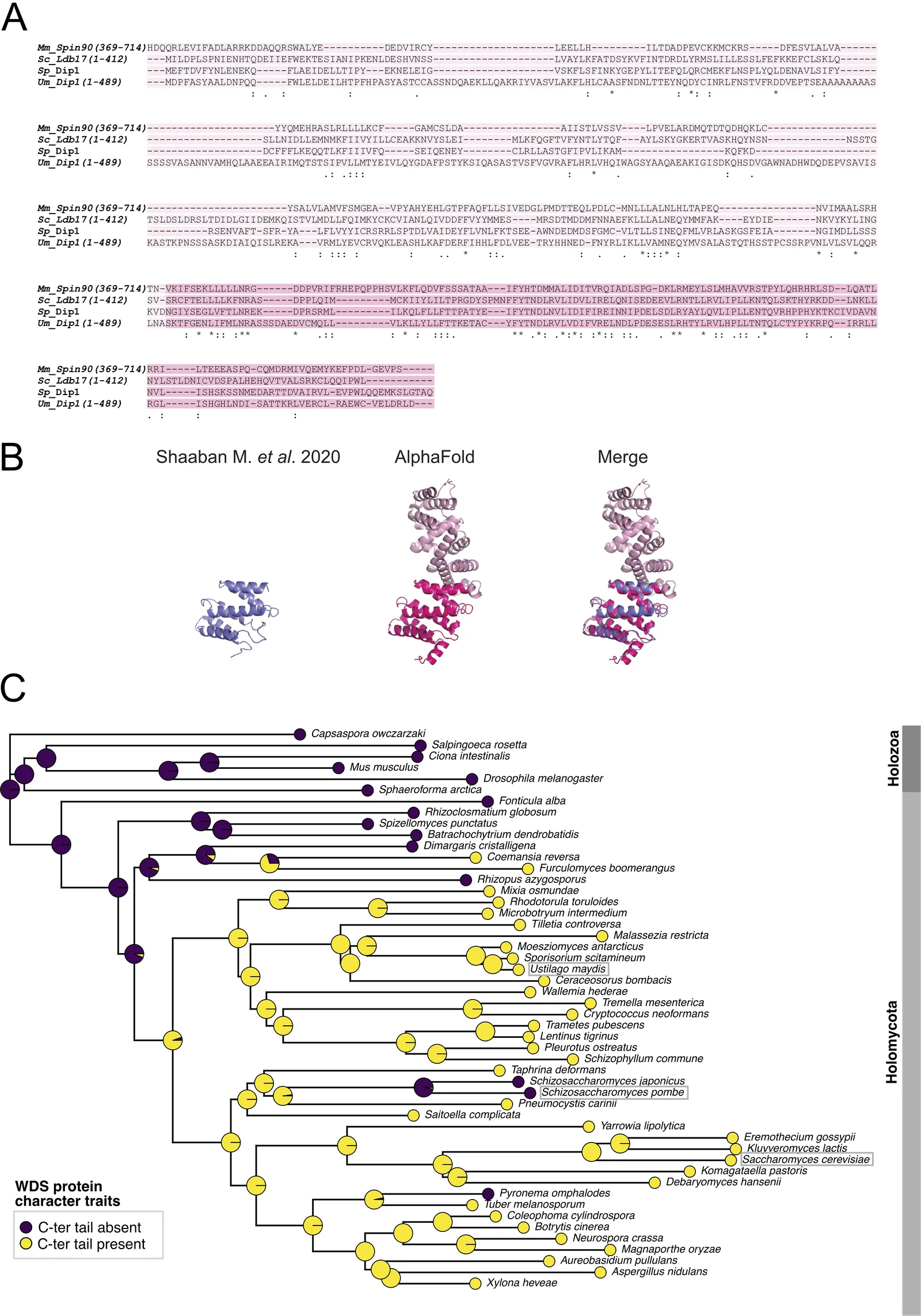
**Alignments and evolution of WDS proteins. (A) **MUSCLE alignment of indicated WDS ortholog regions. Structured WDS core beginning and end were identified using AlphaFold with a variable region show in light pink and a constrained region in magenta. **(B)***S. pombe* Dip1 structure (https://www.ncbi.nlm.nih.gov/pmc/articles/PMC8717925/) and AlphaFold prediction for *S. pombe* Dip1. The variable region of the WDS core is in light pink and the constrained region in magenta. Both structures were aligned and oriented with PyMOL. **(C)** Stochastic character mapping of the presence or absence of a C-terminal tail in WDS homologs in Opisthokonta.

The biochemical difference between Ldb17 and Dip1 indicates that these proteins may differ in the way their activities are regulated. To better understand the molecular basis of this difference and its evolution, we compared sequences of WDS protein homologs from a range of different organisms. Homology searches revealed that WDS family proteins are present in the supergroup Amorphea, which includes animals, fungi, and Amoebozoa. We identified sequences of WDS orthologs from several species across these clades (Supplemental text), aligned the sequences, and compared their features. WDS proteins share a homologous central core region, which has been shown to be composed of alpha-helical repeats (Fig. 4 A) (Luan et al., 2018; Shaaban et al., 2020). In Dip1 and SPIN90, the C-terminal portion of this core region was demonstrated to bind and activate the Arp2/3 complex (Luan et al., 2018; Shaaban et al., 2020). This C-terminal portion has the highest degree of sequence conservation among the orthologs (Fig. S2 A) (Liu et al., 2022). In comparison, the N-terminal half of the core region diverges more in sequence (Fig. 4 A and Fig. S2 A). The function of the N-terminal part of the core is not as well characterized, but it may interact with regulatory proteins (Luan et al., 2018). However, additional sequence features were found in different groups. In Holozoa, the WDS orthologs, like Spin90, have an N-terminal extension that often harbors an SH3 domain (Fig. 4 B; Cao et al., 2020; Kim et al., 2006b; Lim et al., 2003). SH3 domains are a common feature of actin and endocytic proteins and are established PRR interaction domains (Hummel and Kaksonen, 2023; Tonikian et al., 2009). In contrast to Holozoa, a common feature of orthologs in the Holomycota is a C-terminal tail region that includes a PRR that is often flanked by an arginine or lysine charge–rich patch (Fig. 4 B and Supplemental text). The C-terminal tail was, however, missing in several fungal species. To understand the evolutionary history of the C-terminal tail in fungi, we performed an ancestral state reconstruction. This analysis showed that the C-terminal tail was likely an ancient feature that emerged early in Dikarya (Fig. S2 C). Notably, the *S. pombe* ortholog represents a lineage where the ancestral C-terminal tail was lost (Fig. S2 C). This indicates that the *S. pombe* Dip1 has undergone an evolutionary simplification in its structure and possibly in its interactions and regulation.

The structures of the C-terminal core regions of *S. pombe* Dip1 and the whole core for mammalian Spin90 were experimentally solved (Liu et al., 2022; Luan et al., 2018). We generated AlphaFold v2.0 structure predictions for the orthologs in *S. cerevisiae*, *S. pombe*, *U. maydis*, and *Mus musculus* (Fig. 4 C). The structure prediction of *S. pombe* Dip1 conformed well to the published experimental structure (Fig. S2 B). The AlphaFold predictions suggested a structured WDS core for each ortholog (Fig. 4 C and Fig. S2). The predictions also suggested that the extensions, whether N- or C-terminal, are largely unstructured apart from a few potential helical regions and the SH3 domain in *M. musculus*. This analysis suggests that the ancestral form of WDS proteins was likely composed of only the structured core. In opisthokonts, the WDS proteins gained N- or C-terminal regions, which may regulate activity or recruitment of the protein, likely involving PRR-SH3 interactions. The Dip1 protein of *S. pombe* represents a reversion to pre-opisthokont-like core-only state. These changes may contribute to the functional differences between Dip1 and Ldb17.

### Ldb17 function at the endocytic site is linked to Sla1

**Figure 5.**
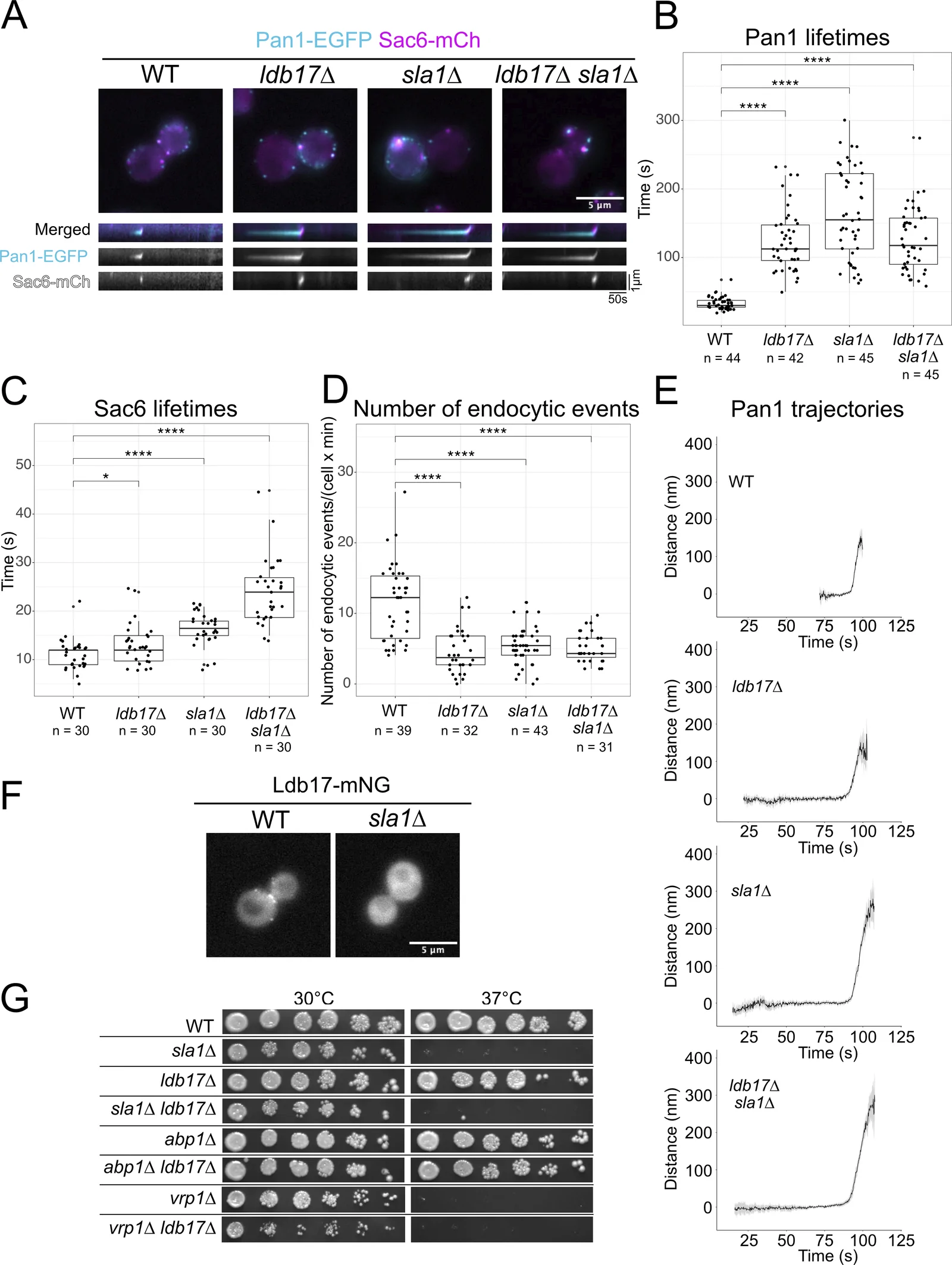
**Ldb17 function at endocytic sites. (A)** *S. cerevisiae* equatorial epifluorescence images for the coat marker Pan1-EGFP, the actin marker Sac6-mCh in WT, *ldb17Δ*, *sla1Δ*, and *ldb17Δ sla1Δ* cell backgrounds, and associated kymographs. **(B)** Boxplot of Pan1-EGFP lifetime in WT, *ldb17Δ*, *sla1Δ*, and *ldb17Δ sla1Δ* cell backgrounds. Pan1 lifetime difference is significantly different between *ldb17Δ* and *sla1Δ* and between *sla1Δ* and *ldb17Δ sla1Δ* at a two stars level. Pan1 lifetime difference between *ldb17Δ* and *ldb17Δ sla1Δ* is not significant. *n* is the number of patches taken from 5 to 10 cells. **** = P ≤ 0.0001. **(C)** Boxplot of Sac6-mCh lifetime in WT, *ldb17Δ*, *sla1Δ*, and *ldb17Δ sla1Δ* cell backgrounds. *n* is the number of patches taken from 5 to 10 cells. * = P ≤ 0.05 and **** = P ≤ 0.0001. **(D)** Boxplot of the number of endocytic events per cell per minute in WT, *ldb17Δ*, *sla1Δ*, and *ldb17Δ sla1Δ* cell backgrounds. *n* is the number of events measured from 5 to 10 cells. **** = P ≤ 0.0001. **(E)** Trajectory plots of Pan1-EGFP in WT, *ldb17Δ*, *sla1Δ*, and *ldb17Δ sla1Δ* cells. Error is the 95% confidence interval. The trajectory plots were aligned to maximize overlap of average inward movements. **(F)** Equatorial epifluorescence images of Ldb17-mNG in WT and *sla1Δ*. **(G)** Yeast growth assay of eight strains conducted over 2 days at 30 and 37°C.

We next focused on C-terminally tailed Ldb17 to better understand its function at the endocytic site. The *ldb17Δ* resulted in a strongly extended and variable lifetime of the late coat phase followed by relatively normal actin patch assembly and coat internalization (Fig. 2). This phenotype is remarkably similar to the phenotype reported for the *sla1Δ* mutant (Kaksonen et al., 2005). Furthermore, Sla1 and Ldb17 have been reported to directly interact via Sla1’s SH3 domains (Burston et al., 2009). To investigate a possible functional relationship between Ldb17 and Sla1, we analyzed the assembly dynamics of another late coat protein, Pan1, and Sac6 in *ldb17Δ*, *sla1Δ*, and *ldb17Δ sla1Δ* mutants. As expected, Pan1-EGFP behaved similar to Sla1-EGFP in *ldb17Δ* and WT cells (Fig. 2 A, Fig. 5 A, and Video 2). In *sla1Δ* cells, Pan1-EGFP patch lifetimes were extended and highly variable in duration. The Pan1-EGFP patches disappeared with the arrival of Sac6 (Fig. 5 A and Video 2), as previously described (Kaksonen et al., 2005). When both Ldb17 and Sla1 were deleted, the Pan1-EGFP phenotype appeared equivalent to single deletions (Fig. 5 A and Video 2). We quantified Pan1 and Sac6 patch lifetimes and the frequency of endocytic events. These data showed that Pan1 patch lifetime in all three mutants was significantly longer and more variable than in WT (Fig. 5 B). These results suggest that both Ldb17 and Sla1 regulate the initiation of actin assembly. Sac6 patch lifetimes were extended in all three mutants relative to WT, but the double mutant showed an exacerbated phenotype (Fig. 5 C). These data suggest an additive effect on actin patch lifetime, which may not be directly related to the initiation of actin assembly. The number of endocytic events per cell per minute was significantly reduced in all three mutants compared with the WT (Fig. 5 D), which revealed that all three mutants decrease the endocytic efficiency. Actin patch lifetime increases in the mutants may be related to changes in G-actin/F-actin ratios due to the reduced number of actin patches in the cells (Burke et al., 2014). A smaller number of actin patches may lead to larger and more long-lived patches such that total cellular F-actin in patches remains unchanged. We then quantified Pan1 patch internalization trajectories in each strain. In WT and *ldb17Δ* cells, Pan1 internalized ∼150 nm and the internalization trajectories were indistinguishable (Fig. 5 E). In *sla1Δ* and *ldb17Δ sla1Δ* mutants, the internalization trajectories differed from WT. These trajectories internalized a distance of ∼300 nm (Fig. 5 E). These data suggest that Sla1 likely also has functions that are not shared with Ldb17, which are related to internalization of the coat module, but not timing of the initiation. For example, Sla1 may regulate actin assembly via Las17 (Rodal et al., 2003).

We tested the Sla1 dependency of Ldb17 recruitment to patches. In *sla1Δ* cells, Ldb17-mNG patches were not detected (Fig. 5 F). In contrast, Sla1 patch recruitment occurred in *ldb17Δ* cells (Fig. 2 A). In agreement with earlier observations (Burston et al., 2009), these results suggest that Sla1 acts upstream of Ldb17 protein recruitment, consistent with the recruitment time of each protein to the endocytic site (Ldb17 arrives after Sla1). In conclusion, the *sla1Δ* phenotype, particularly the actin initiation delay, is likely due to the failed recruitment of Ldb17 to the patch.

To further understand Sla1-Ldb17 functions, we analyzed genetic interactions. Mutant *sla1Δ* cells have been reported to show synthetic genetic phenotypes with *abp1Δ* and *vrp1Δ* (Costanzo et al., 2010; Holtzman et al., 1993). In contrast, the *ldb17Δ* mutant was reported to suppress *vrp1Δ* phenotypes in systematic studies (Costanzo et al., 2016). To investigate these interactions, we performed yeast growth assays of different mutant combinations at 30 and 37°C. As previously reported (Holtzman et al., 1993), *sla1Δ* mutants had a growth defect at 37°C (Fig. 5 G), while *ldb17Δ* had no detectable growth defect relative to WT at any temperature tested (Fig. 5 G). The *sla1Δ* mutant growth phenotype was epistatic relative to *ldb17Δ* (Fig. 5 G). The *ldb17Δ abp1Δ* mutant grew similar to WT. In contrast to the systematic data, the phenotypic suppression of *vrp1Δ* with *ldb17Δ* was not reproduced in our conditions, but we observed that *ldb17Δ vrp1Δ* cells had a more severe growth defect than *vrp1Δ* cells (Fig. 5 G). This genetic exacerbation of *vrp1Δ* phenotypes is similar for both *ldb17Δ* and *sla1Δ* mutants. Together, these results suggest that Sla1 has both Ldb17-dependent and Ldb17-independent functions.

### The assembly dynamics of Las17 are altered in *ldb17Δ* cells

**Figure 6.**
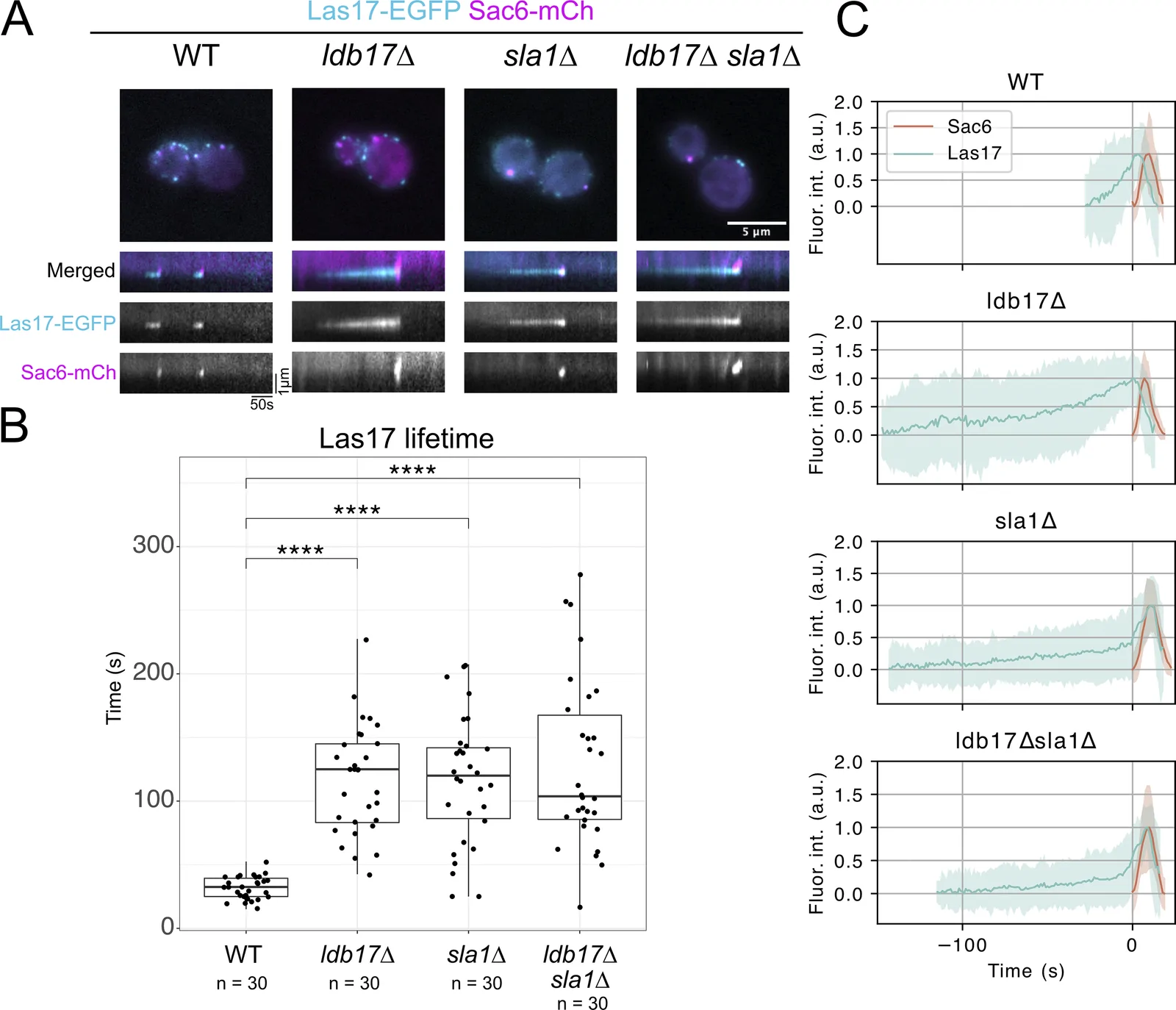
**WASP dynamics in *ldb17* mutants. (A)** *S. cerevisiae* equatorial epifluorescence images for the WASP protein Las17-EGFP, the actin marker Sac6-mCh in WT, *ldb17Δ*, *sla1Δ*, and *ldb17Δ sla1Δ* cell backgrounds, and associated kymographs. **(B)** Boxplot of Las17-EGFP lifetime in WT, *ldb17Δ*, *sla1Δ*, and *ldb17Δ sla1Δ* cell backgrounds. The comparison of the lifetime in the three mutants showed no significant difference. *n* is the number of patches taken from 5 to 10 cells. **** = P ≤ 0.0001. **(C)** Fluorescence intensity profiles of Las17-EGFP aligned with respect to the corresponding Sac6-mCh peaks in WT, *ldb17Δ*, *sla1Δ*, and *ldb17Δ sla1Δ* cell backgrounds.

In *S. cerevisiae*, Las17 (WASP) assembles at endocytic sites about 20 s prior to actin assembly starts. This Las17 pre-assembly is partially mediated by Sla1 (Sun et al., 2017). Sla1 has also been shown to inhibit Las17 nucleation–promoting activity *in vitro* (Rodal et al., 2003). This link is thought to play a role in regulating the timing of actin assembly and WASP concentration at the endocytic site (Sun et al., 2017). We investigated Las17 assembly dynamics at endocytic sites in relation to Ldb17 function. We analyzed Las17-EGFP in *ldb17Δ*, *sla1Δ*, and *ldb17Δ sla1Δ* cells that co-expressed Sac6-mCh. In WT cells, Las17-EGFP pre-assembles at endocytic sites and disappears after actin assembly (Fig. 6 A and Video 2; Kaksonen et al., 2003). In *ldb17Δ* cells, *sla1Δ* cells, and *ldb17Δ sla1Δ* cells, the time that Las17 resided at the membrane prior to actin assembly was dramatically increased compared with the WT cells (Fig. 6 A). Quantification revealed that in WT cells, Las17 started to pre-assemble ∼20 s before the start of actin assembly. In all three mutants, the pre-assembly period was extended and highly variable with an average of over 100 s (Fig. 6B). These observations are consistent with the extended lifetimes of Pan1 and Sla1 (Fig. 2 C and Fig. 5 B) and indicate that the normal timing of actin assembly is impaired in *ldb17Δ* and *sla1Δ* mutants. We next quantitatively analyzed the fluorescence intensity of Las17-EGFP and Sac6-mCh over time for WT and the mutants. In WT cells, Las17 intensity steadily ascends and peaks with actin assembly, before rapidly disappearing (Fig. 6 C). In *ldb17Δ*, *sla1Δ*, and *ldb17Δ sla1Δ* cells, the ascending phase occurred throughout the extended pre-assembly phase (Fig. 6 C). In *ldb17Δ* cells, like WT, the ascending phase was continuous and reached its maximum with actin polymerization. In contrast, *sla1Δ* and *ldb17Δ sla1Δ* cells had a steady ascending phase, which was followed by a rapid intensity spike when actin assembly appeared (Fig. 6 C). These results suggest that Sla1 has at least two activities on Las17. One activity regulates the timing of actin assembly, likely via Ldb17, and a second activity contributes to the pre-assembly of Las17 prior to actin assembly.

### Ldb17 regulation via SH3 proteins Bzz1 and Sla1

**Figure 7.**
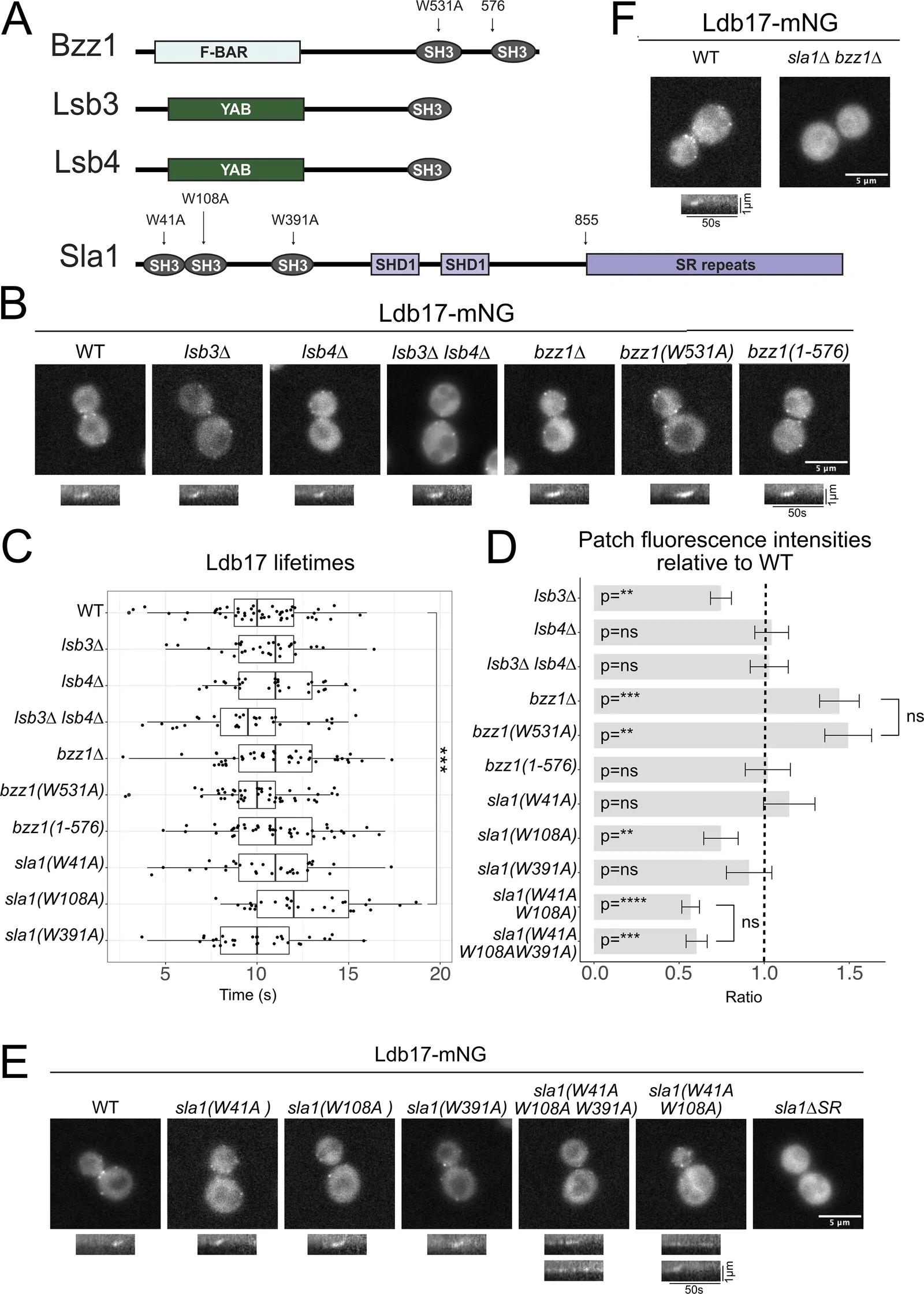
**Ldb17 recruitment and regulation. (A)** Structure of Bzz1, Lsb3, Lsb4, and Sla1. **(B)** Equatorial epifluorescence images of Ldb17-mNG in WT, *lsb3Δ*, *lsb4Δ*, *lsb3Δ lsb4Δ*, *bzz1Δ*, *bzz1(W531A)*, *bzz1(1–576)*, and associated kymograph. **(C)** Boxplot of Ldb17-mNG lifetime in WT (*n* = 45), *lsb3Δ* (*n* = 30), *lsb4Δ* (*n* = 30), *lsb3Δ lsb4Δ* (*n* = 30), *bzz1Δ* (*n* = 45), *bzz1(W531A)* (*n* = 45), *bzz1(1–576)* (*n* = 45), *sla1(W41A)* (*n* = 30), *sla1(W108A)* (*n* = 30), and *sla1(W391A)* (*n* = 30). All the compared lifetimes are not significantly different except the indicated one. *n* is the number of patches taken from 5 to 10 cells. **(D)** Bar plot of Ldb17-mNG patch fluorescence intensity in WT, *lsb3Δ*, *lsb4Δ, lsb3Δ lsb4Δ*, *bzz1Δ*, *bzz1(W531A)*, *bzz1(1–576)*, *sla1(W41A)*, *sla1(W108A)*, *sla1(W391A)*, *sla1(W41AW108A)*, and *sla1(W41AW108AW391A)* mutants (*n* = 30 for all). *n* is the number of patches taken from 5 to 10 cells. The P values on the bar plot represent a U test for the comparison between the mutants and their WT. The P values on the right of the bar plot represent a Z test between mutants. **(E)** Equatorial epifluorescence images of Ldb17-mNG in *sla1(W41A)*, *sla1(W108A)*, *sla1(W391A)*, *sla1(W41A W108A)*, *sla1(W41A W108A W391A)*, and *sla1(1–855)* mutants. Associated kymographs are displayed below. **(F)** Equatorial epifluorescence images of Ldb17-mNG in WT and *sla1Δ bzz1* and associated kymographs. For all plots ns = P > 0.05; ** = P ≤ 0.01; *** = P ≤ 0.001; and **** = P ≤ 0.0001.

We next sought to better understand Ldb17 regulation and recruitment to endocytic sites. The PRR in the unstructured tail of Ldb17 (Fig. 4) suggests a possible link with SH3 domain proteins, which could function as regulators or recruiters for Ldb17. We selected four candidate proteins for Ldb17 interactions: Lsb3 and Lsb4 (genome-duplicated paralogs), Bzz1, and Sla1 (Fig. 7 A), which have been reported to interact with WDS proteins and arrive at endocytic sites before Ldb17 (Burston et al., 2009; Kim et al., 2006a; Tonikian et al., 2009).

We designed mutants (point mutants, truncations, or gene deletions) for these candidates to target specific SH3 domains (Fig. 7 A) and observed the consequences on Ldb17-mNG assembly dynamics. When Lsb3 and Lsb4 were deleted, either separately or in combination, this had no qualitative effect on Ldb17 recruitment to patches (Fig. 7 B). No major differences were detected in Ldb17 patch lifetimes (Fig. 7 C). Ldb17 patch intensities in WT and *lsb4Δ* mutants were similar, but a mild decrease was detected for *lsb3Δ* mutants (Fig. 7 D). Bzz1 deletion resulted in Ldb17-mNG recruited to patches and internalized as WT with a similar lifetime (Fig. 7, B and C). However, in *bzz1Δ* cells Ldb17 patches were brighter than WT (Fig. 7 D), which is consistent with earlier observations (Burston et al., 2009). Bzz1 contains two SH3 domains (Fig. 7 A), and to identify which SH3 is behind this intensity effect, we used a point mutation in the first SH3 domain and a C-terminal truncation of the second one (Fig. 7 A), which were previously generated (Hummel and Kaksonen, 2023). Only the mutation of the first SH3 domain resulted in brighter Ldb17 patches that matched the *bzz1Δ* null phenotype (Fig. 7, B–D). These results indicate that specifically the first SH3 domain of Bzz1 has a function that antagonizes Ldb17 recruitment to endocytic sites.

For Sla1, which contains three SH3 domains (Fig. 7 A), we used SH3 point mutations in the canonical PRR-binding pocket, based on previous mutants (Sun et al., 2017). These SH3 domain mutants were analyzed individually or in combinations to assess Ldb17 patch dynamics. When each SH3 domain was mutated individually, *sla1(W41A)*, *sla1(W108A)*, or *sla1(W391A)*, we found that Ldb17 was recruited to the membrane and internalized similar to WT in each single SH3 mutant (Fig. 7 E). Ldb17 intensities were unaffected in *sla1(W41A)* and *sla1(W391A)* mutants but slightly dimmer in *sla1(W108A)* (Fig. 7 D). Furthermore, Ldb17 patch lifetimes of *sla1(W41A)* and *sla1(W391A)* cells did not differ from WT, but a significant increase in Ldb17 lifetime was detected in *sla1(W108A)* (Fig. 7 C). In combination, we found that mutating the first and second SH3 (W41A and W108A) or all three SH3 domains (W41A, W108A, and W391A) resulted in novel Ldb17 patch behavior. In these mutants, Ldb17 patches were dimmer than the WT (Fig. 7 D). The Ldb17 patches persisted longer than the WT at the plasma membrane, and the fluorescence signal fluctuated rapidly (Fig. 7 E). Due to this flickering behavior, we could not determine the average lifetime of the patches. Ldb17 patches were rarely internalized in *sla1(W41A and W108A)* and never internalized in *sla1(W41A, W108A*, *and W391A)* (Fig. 7 E). Together, these results suggest that all three SH3 domains of Sla1 contribute to Ldb17 regulation although the first two SH3 domains are most essential, with the second SH3 domain having the predominant role. The Sla1 SH3 domains appear to be unessential for general Ldb17 recruitment. However, specific Ldb17 regulatory roles exist for the SH3 domains of Sla1 and the first SH3 domain of Bzz1. Ldb17 recruitment requires Sla1 (Fig. 5 F); therefore, we analyzed Ldb17 recruitment in a C-terminal deletion mutant that lacks the (Sla1 LxxQxTG repeat) SR region (Sun et al., 2017). In *sla1-ΔSR* mutants, Ldb17 patches were undetectable, similar to the null mutants (Fig. 7 E). Together, these results suggest that the SR repeats of Sla1 are critical for recruiting Ldb17 to patches, while the SH3 domains have roles in regulating Ldb17 at endocytic sites.

### Ldb17 and Dip1 differ in WASP synergy *in vitro*

**Figure 8.**
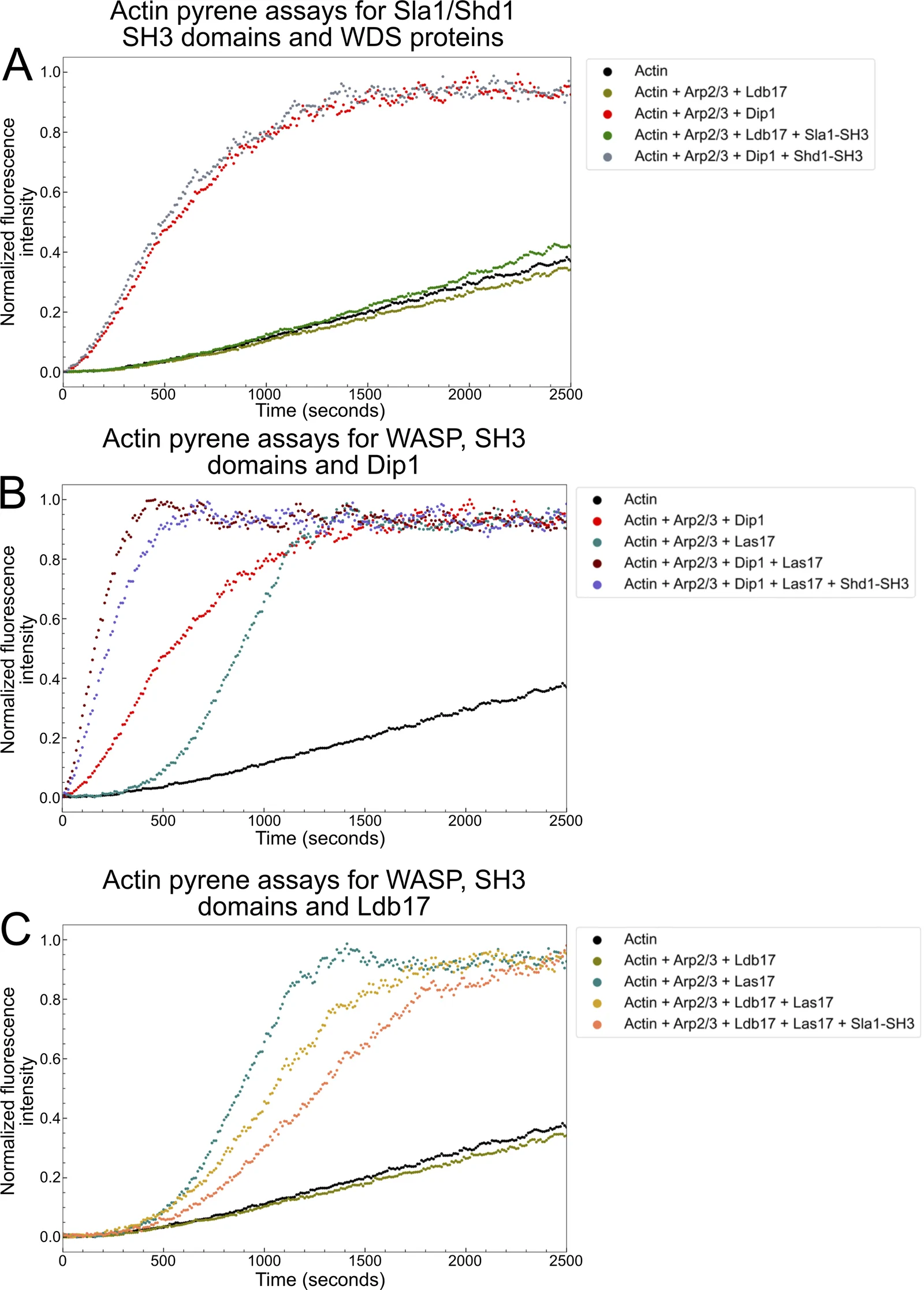
**Ldb17 and Dip1 Arp2/3 complex activation with WASP and Sla1 SH3 domains. (A–C)** Time course of actin polymerization monitored by pyrene fluorescence. The protein concentrations are 2 µM for actin, 0.1 µM for Arp2/3 complex, and 1 µM for all the other proteins. Controls are replotted from Fig. 2 F. Pyrene assays were performed together for fair comparison across conditions.

Given the Sla1 SH3 results and potential PRR-SH3 interactions, we reasoned that the Ldb17 proline-rich tail interactions with Sla1 SH3 domains may be sufficient to activate the Arp2/3 complex activation activity of Ldb17. Therefore, we purified the N-terminal fragment of Sla1 (1–415) and Shd1 (1–569), which contains the SH3 domains, and performed fluorometric pyrene actin polymerization assays to measure the Arp2/3 complex activation. The addition of the SH3-Shd1 fragment to Dip1 had no detectable effect on the Arp2/3 complex activation activity of Dip1 (Fig. 8 A). Similarly, the SH3-Sla1 fragment did not affect Arp2/3 complex activation of Ldb17 (Fig. 8 A). These data suggest that the interaction between an SH3 domain of Sla1 and the proline-rich region of Ldb17 is not sufficient to activate Ldb17. The Sla1 SH3 domains also interact with the PRRs of Las17 (Rodal et al., 2003; Sun et al., 2017). In *S. pombe*, Dip1 is proposed to synergize with the WASP homolog Wsp1, which suggests a more complex relationship may exist between WASP and WDS proteins. We therefore tested the potential combinatorial effects of Las17, the WDS proteins, and the Sla1 orthologs. The addition of Las17 and Dip1 together resulted in an increase in actin nucleation, which was greater than with either Las17 or Dip1 alone (Fig. 8 B) and comparable to previous observations that suggested an activator synergy (Balzer et al., 2020). However, the addition of the SH3 fragment of Shd1 to the two Arp2/3 complex activators reduced the synergistic effects (Fig. 8 B), possibly due to negative regulation of WASP by Sla1 (Rodal et al., 2003). Ldb17 showed no ability to synergize with WASP, and, in contrast, reduced the Arp2/3 complex activation ability of WASP (Fig. 8 C). This may suggest that Dip1 and Ldb17 prefer to bind to different Arp2/3 complex conformations. The addition of Sla1 SH3 domains to the dual activator condition reduced actin nucleation similar to the *S. pombe* proteins (Fig. 8 C). These results suggest that *S. cerevisiae* Ldb17 is subject to an additional layer of regulation compared with *S. pombe* Dip1. The SH3 domains of Sla1 are insufficient to activate Ldb17 *in vitro, which* suggests that an additional activator is needed *in vivo* or that the SH3 domains may instead regulate an aspect of Ldb17 function other than the Arp2/3 activation.

### The Ldb17 tail negatively regulates activity

**Figure 9.**
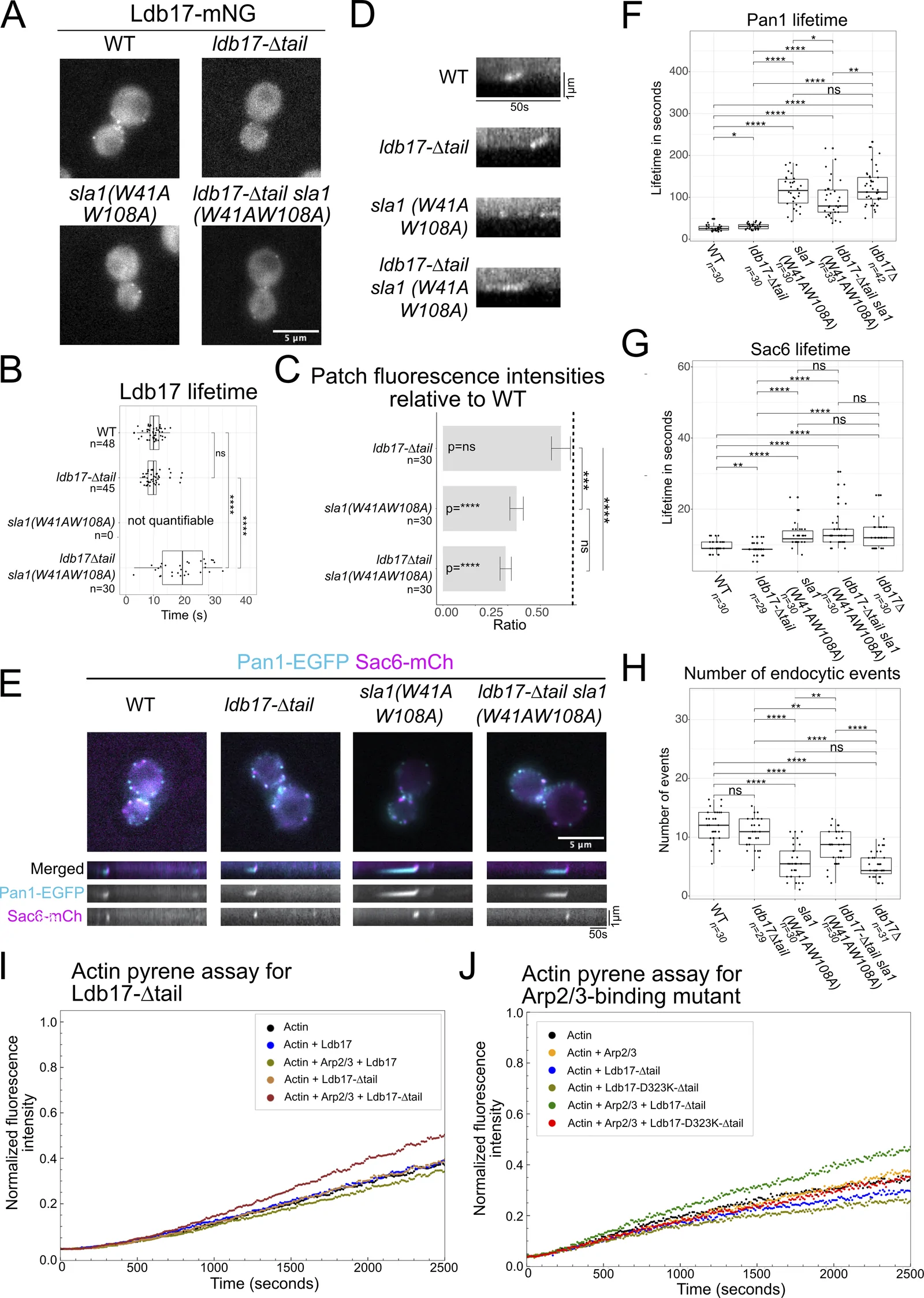
**Ldb17 tail regulation. (A)** Equatorial epifluorescence images of Ldb17-mNG in WT, *ldb17Δtail*, *sla1(W41A W108A)*, and *ldb17Δtail sla1(W41A W108A)*. **(B)** Boxplot of Ldb17-mNG lifetime in WT, *ldb17Δtail*, *sla1(W41A W108A)*, and *ldb17Δtail sla1(W41A W108A)* cells. *n* is the number of patches taken from 5 to 10 cells. **(C)** Bar plot of Ldb17-mNG patch fluorescence intensity in *ldb17Δtail*, *sla1(W41A W108A)*, and *ldb17Δ*tail *sla1(W41A W108A)* cells. The P values on the bar plot represent a U test for the comparison between the mutants and their WT. The P values on the right of the bar plot represent a Z test between mutants. *n* is the number of patches taken from 5 to 10 cells. **(D)** Kymographs of indicated Ldb17-mNG patches. **(E)***S. cerevisiae* equatorial epifluorescence images for the coat marker Pan1-EGFP, the actin marker Sac6-mCh in WT, *ldb17Δtail*, *sla1(W41A W108A)*, and *ldb17Δtail sla1(W41A W108A)* cells, and associated kymographs. **(F)** Boxplot of Pan1-EGFP lifetime in WT, *ldb17Δtail*, *sla1(W41A W108A)*, *ldb17Δtail sla1(W41A W108A)* and *ldb17Δ *cells. *n* is the number of patches taken from 5 to 10 cells. **(G)** Boxplot of Sac6-mCh lifetime in WT, *ldb17Δtail*, *sla1(W41A W108A)*, *ldb17Δtail sla1(W41A W108A)* and *ldb17Δ *cells. *n* is the number of patches taken from 5 to 10 cells. **(H)** Boxplot of the number of endocytic events per cell per minute in WT, *ldb17Δtail*, *sla1(W41A W108A)*, *ldb17Δtail sla1(W41A W108A) and ldb17Δ* cells. *n* is the number of events measured from 5 to 10 cells. **(I and J)** Time course of actin polymerization monitored by pyrene fluorescence. The protein concentrations are 2 µM for actin, 0.1 µM for Arp2/3 complex, and 1 µM for all the other proteins. Controls are replotted from Fig. 2 F, Fig. 3 A, and Fig. 9. Pyrene assays were performed together for fair comparison between conditions. For all plots ns = P > 0.05; * = P ≤ 0.05; ** = P ≤ 0.01; *** = P ≤ 0.001; and **** = P ≤ 0.0001.

We then wanted to investigate the function of the C-terminal tail of Ldb17 *in vivo* and its potential interaction with the SH3 domains of Sla1. We generated an Ldb17 mutant with a truncated C terminus (a.a. 1–432; ldb17-Δtail). We then focused on the first two SH3 domains of Sla1 that have a potent effect on Ldb17 dynamics (Fig. 7 E). We imaged the Ldb17-Δtail protein tagged with mNG in WT and the sla1(W41A and W108A) mutant, which predominantly affected Ldb17 patch dynamics (Fig. 7 E). We found that like the full-length protein, Ldb17-Δtail was recruited to patches in both conditions (Fig. 9 A). Quantification revealed modest reduction in Ldb17 fluorescence intensity and shortening of actin patch lifetime in *ldb17-Δtail* mutants relative to WT (Fig. 9, C and G). Ldb17-mNG in the *sla1(W41A and W108A)* mutant was dim and flickering as before, which prevented the determination of the beginning and end of a patch lifetime (Fig. 7 C and Fig. 9 D). In contrast, Ldb17-Δtail-mNG in the *sla1(W41A and W108A)* mutants appeared to form stable, dim patches that internalized (Fig. 9 D, kymographs), but their lifetime was extended relative to WT cells (Fig. 9, B and D). These results are consistent with a negative regulatory role of the Ldb17 tail. The loss of the inhibitory function would not impede Ldb17 function. The Sla1 SH3 domain mutant fails to correctly regulate WT Ldb17, resulting in aberrant patch dynamics. In the double mutant, both the regulator and the regulation are removed and activity is restored. Therefore, the Sla1 SH3 domains likely act via the Ldb17 tail, but the rescue is only partial, so a more complex regulation likely exists.

Inhibitory roles of the Ldb17 tail were further analyzed with Pan1 and Sac6 behavior in different mutants. As previously reported (Sun et al., 2017), we found that Pan1 lifetimes were extended in the *sla1(W41A and W108A)* mutant, like *sla1Δ* mutants (Fig. 2; and Fig. 9, E and F). Pan1 and Sac6 lifetimes in WT and *ldb17-Δtail* were similar (Fig. 9, E–G). In contrast, the *ldb17-Δtail* in combination with *sla1(W41A and W108A)* resulted in a mild suppression of the Pan1 lifetime phenotype (Fig. 9, E and F). A mild suppression was not detectable in the fimbrin lifetimes (Fig. 9 G), but the number of endocytic events was partially rescued in the double mutant (Fig. 9 H). Together, these results further indicate that the Ldb17 tail has an inhibitory role and that the Ldb17-Δtail protein retains functionality. The partial rescue of the *sla1(W41A and W108A)* phenotype by the tailless protein supports a significant role of the Sla1 SH3 domains to relieve that inhibition. Since the rescue remains only partial, other mechanisms likely contribute to the *sla1(W41A and W108A)* mutant phenotype, possibly through an interaction with Las17 (Sun et al., 2017), or Bzz1 SH3 domain competition (Hummel and Kaksonen, 2023).

We next tested whether removal of the tail could change the Arp2/3 complex activation of Ldb17 *in vitro*. Ldb17-Δtail was monomeric and monodisperse like the full-length protein (Fig. S1). A small but measurable increase in Arp2/3 complex activation was detected, relative to a full-length protein in a pyrene assay (Fig. 9 I), which further indicates an autoinhibition by the C-terminal tail. The increase was dwarfed by Dip1 activity (Fig. 2 F and Fig. 9 I). We then purified the Arp2/3 complex–binding mutant Ldb17-D323K-Δtail and tested whether it affected Arp2/3 complex activation. The Arp2/3 complex activation was lost in the Ldb17-D323K-Δtail (Fig. 9 J), which demonstrates that Ldb17 Arp2/3 complex activation is similar to Dip1 mechanistically; however, the tail modulates this activity. The mild *in vitro* activity further suggests that the tail may not be the only factor required to fully activate Ldb17, which aligns with our *in vivo* results. In addition, the Arp2/3 complex activation differences may also reflect a divergence in the actin initiation requirements of *S. cerevisiae* (pre-assembly) and *S. pombe* (co-assembly) endocytosis mechanisms.

## Discussion

The WDS proteins are conserved regulators of actin assembly of Opisthokonta, with the most detailed understanding of WDS protein function coming from *S. pombe* Dip1 studies (Basu and Chang, 2011; Shaaban et al., 2020; Balzer et al., 2018; Balzer et al., 2020; Wagner et al., 2013). These studies showed that in contrast to WASP, Dip1 binds to and activates the Arp2/3 complex to nucleate an actin filament without the need for a preexisting “mother filament” (Balzer et al., 2020). Recruitment of Dip1 to the endocytic site could thereby form the first actin filaments, which would then allow WASP to further activate Arp2/3 complexes and start the branched actin network assembly. Much work on the regulation of Arp2/3 complex–mediated actin assembly during endocytosis is done in *S. cerevisiae* (Sun et al., 2006; Sun et al., 2017; Rodal et al., 2003). A concept of a temporally regulated actin switch was proposed for *S. cerevisiae* endocytosis (Sun et al., 2017), but the exact molecular mechanism has remained unknown. An endocytic role was demonstrated for *S. cerevisiae* Ldb17 over 15 years ago (Burston et al., 2009), but since then, the protein was largely overlooked in *S. cerevisiae* endocytosis literature, perhaps due to the low intrinsic Arp2/3 complex activation activity of Ldb17.

Here, we show that like *S. pombe*, the WDS homologs in two other fungal species (*S. cerevisiae* and *U. maydis*) localize to endocytic sites just prior to actin assembly, and that the deletion phenotype of *S. cerevisiae* Ldb17 mirrors the Dip1 deletion phenotype in *S. pombe*. These findings show a conserved WDS protein role in triggering endocytic actin assembly within fungi. Despite this functional conservation, WDS protein regulation is surprisingly different between *S. cerevisiae* and *S. pombe*. The Arp2/3 complex activation activity of Ldb17 is partially controlled by a mechanism that involves SH3-PRR interactions, whereas Dip1 is biochemically constitutively active. This variation of molecular mechanisms, while the cellular function is maintained, offers a basis for investigating the regulatory evolution in clathrin-mediated endocytosis.

When the actin on-switch mechanism is inactivated by mutations in WDS protein or Sla1, the timing of endocytic actin assembly appears random and cytoplasmic actin filaments may stochastically provide mother filaments for Arp2/3 complex activation (Basu and Chang, 2011; Chen and Pollard, 2013). Similar actin nucleation timing defects have been reported for several endocytic mutants associated with *S. cerevisiae* Sla1 and Las17 functions (*sla1Δ*, Sla1 SH3 mutants, Sla1 clathrin-binding mutants, Las17-ΔVCA mutants, Las17 phosphorylation mutants, and Pan1 mutants) (Chi et al., 2012; Feliciano and Di Pietro, 2012; Kaksonen et al., 2005; Sun et al., 2006; Sun et al., 2017; Tolsma et al., 2018; Tyler et al., 2021). It has been challenging to interpret these observations in a context of the biochemical functions of Sla1 and Las17 alone. Understanding the Ldb17 function in controlling actin assembly allows reinterpretation of these observations.

**Figure 10.**
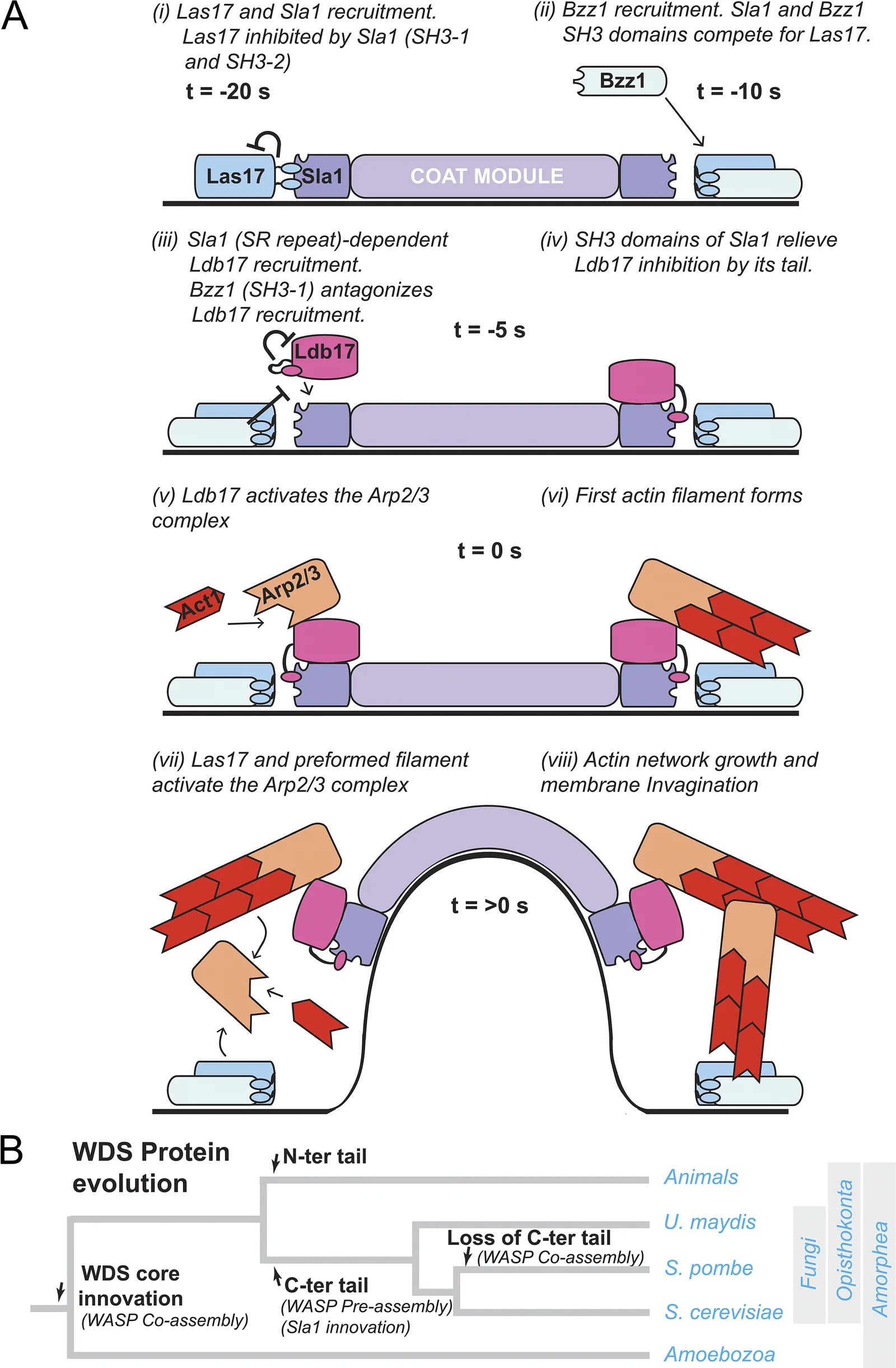
**Model of Ldb17 function at budding yeast endocytic sites. (A)** Schematic representation of Ldb17 recruitment and function during *S. cerevisiae* endocytosis. **(B)** Phylogenetic tree indicating clathrin-mediated endocytic divergences.

We propose the following working model for the functions of *S. cerevisiae* Ldb17 in the regulation of endocytic actin assembly (Fig. 10 A). First, Pan1 and End3 start assembling at endocytic sites quickly followed by Las17 and Sla1 (Kaksonen et al., 2005; Sun et al., 2015). This co-arrival of proteins marks the transition from the early to the late phase of endocytosis (Carroll et al., 2012). The first two SH3 domains of Sla1 inhibit Las17 *in vitro* (Rodal et al., 2003). Subsequently, Bzz1 arrives at endocytic sites and its two SH3 domains compete with Sla1 for binding to a PRR of Las17 (Feliciano and Di Pietro, 2012; Hummel and Kaksonen, 2023; Soulard et al., 2005; Sun et al., 2006). Ldb17 has a unique recruitment timing among endocytic proteins just before the start of actin assembly (Burston et al., 2009; Sun et al., 2006). The recruitment of Ldb17 is dependent on the Sla1 C-terminal SR repeats, while the activity of Ldb17 is mediated by the SH3 domains of Sla1, likely via an interaction between the SH3 domains and the PRR in the Ldb17 tail. Furthermore, we found that the first SH3 domain of Bzz1 antagonizes Ldb17 assembly, possibly controlling its amount at the endocytic sites. The activation of the Arp2/3 complex by Ldb17 creates the first actin filament in close proximity to pre-assembled Las17. The transition of Sla1 SH3 domain interaction from the Las17 PRR to the Ldb17 PRR could simultaneously activate both Las17 and Ldb17. The new actin filament along with another Arp2/3 complex and Las17 initiates the assembly of the branched actin network at the endocytic site to power membrane invagination (Fig. 10 A). The inward movement of Ldb17 with Sla1 away from Las17 could spatially separate their activities during internalization. The regulation of Ldb17 activity is likely more complex than depicted in this model, as the loss of the Ldb17 tail only partially rescued *sla1* mutants and removal of the tail had mild activity relative to Dip1 on Arp2/3 complex–mediated actin assembly (Fig. 9). Furthermore, our data show that Sla1 also has Ldb17-independent activities, as the *sla1Δ* cells have unique phenotypes such as the increased coat internalization (Fig. 5) and a partial co-assembly of Las17 with actin (Fig. 6). Further research is needed for deciphering these mechanisms.

In *S. pombe*, entry into the late phase of the endocytic pathway differs from the one depicted for *S. cerevisiae* in steps i-iv of Fig. 10 A. In *S. cerevisiae*, Pan1, Sla1, and WASP arrive essentially together, and then, the WASP module proteins assemble in a stepwise progression (Vrp1 with Bzz1, then Myo3/5, Bbc1, and Aim21) (Kaksonen et al., 2005; Sun et al., 2006; Tonikian et al., 2009). In contrast, *S. pombe* Sla1 arrives after Pan1 (Picco et al., 2025; Sirotkin et al., 2010). Sla1 is followed by the concurrent assembly of Dip1, Wsp1, Bzz1, and other WASP module-associated proteins (Sirotkin et al., 2010; MacQuarrie et al., 2019). Approximately 1 s after the arrival of these proteins, Arp2/3 complex–dependent actin assembly begins. After this point, the endocytic progression in *S. pombe* is likely very similar to that of *S. cerevisiae* (Fig. 10 A, steps v-viii). *S. pombe* Dip1 lacks a C-terminal PRR and likely cannot interact with SH3 domains. Other differences between the Ldb17 and Dip1 structured cores may also contribute to regulatory changes (Fig. 4). The differences we identified demonstrate how regulatory variation between species can inform the mechanisms of clathrin-mediated endocytosis.

Several dynamic and mechanistic traits of clathrin-mediated endocytosis have diverged within fungi (Picco et al., 2025; Sun et al., 2019). The WDS proteins represent another intriguing divergence. The C-terminal unstructured tail with a PRR appears in fungal WDS proteins. The appearance of the WDS tail correlates with the appearance of Sla1 early in fungal evolution (Fig. 4). Deletion of the Ldb17 tail leads to subtle phenotypic changes and does not impede the actin on-switch function. This suggests that the Ldb17 tail serves a fine-tuning regulatory role but is likely not an integral part of the on-switch mechanism. The mild phenotype of the tail loss may have facilitated this mutation, which appeared independently in a few fungal groups including *Schizosaccharomyces* (Fig. 4). Both *U. maydis* and *S. cerevisiae* pre-assemble WASP prior to actin assembly at endocytic sites and have a C-terminal tail in their WDS proteins, while *S. pombe* co-assembles WASP together with actin and has a tailless Dip1 (Fig. 10 B). An intriguing possibility is that the WASP assembly mode and WDS protein tail status are causally linked. The loss of the regulatory tail could have facilitated the evolution of the less elaborate, simultaneous assembly of WASP and its regulators together with actin. Alternatively, the loss of the WDS tail became possible with the changes in WASP assembly. These changes may allow *S. pombe* to generate large actin patches and longer coat movement relative to other species (MacQuarrie et al., 2019; Sun et al., 2019; Picco et al., 2025). Interestingly, the holozoan Spin90 has an analogous unstructured tail N-terminally attached to the conserved WDS core, which has both PRRs and an SH3 domain. This suggests that the PRR-SH3–based regulation may have been invented independently in animals and fungi. Shared features between endocytic pathways like WASP co-assembly (mammalian cells and *S. pombe*) and SH3-PRR regulations (mammalian cells and *S. cerevisiae*) offer opportunities to dissect how clathrin-mediated endocytosis evolves mechanistically but still preserves process functionality.

## Materials and methods

### Strains and plasmids

All strains in this study are listed in Table S1. The strains were generated either by transformation using homologous recombination or by mating, sporulation, and subsequent tetrad dissection. General yeast growth, transformation, and mating manipulations were done according to Dunham et al. (2015); Hagan et al. (2016). For the *S. cerevisiae* transformation, constructs were made by PCR amplification of a DNA fragment containing a selection cassette (Janke et al., 2004), and then confirmed by colony PCR and sequencing. Internal point mutations of Sla1 were generated by first constructing the desired mutant gene in a pMK0045 plasmid using PCR mutagenesis. The mutated Sla1 sequence was then amplified by PCR using long overlapping primers. For *U. maydis* and *S. pombe* transformations, plasmid constructs were built with up to 1,000-bp flanking regions, linearized and integrated into the desired locus, and verified by PCR as described above (Bösch et al., 2016). For Ldb17, the D323K mutation was introduced by PCR mutagenesis into plasmid pMK0411 (pCoofy6-Ldb17). The mutated ldb17 sequence was then amplified by PCR using overlapping primers and transformed into the *ldb17*Δ strain. After URA deletion on a 5-FOA plate, this strain was further tagged with mNG. All plasmids used in this study are listed in Table S2.

### Media, buffers, and growth conditions

All the media were prepared following the standard protocols (Treco and Lundblad, 1993). All medium formulations are listed in Table S3. *S. cerevisiae* strains were grown at 24 or 30°C on 2% agar plates supplemented with YPD. *S. pombe* and *U. maydis* strains were grown at 24°C on 2% agar plates supplemented with YES and YEPS-Light, respectively.

### Protein purification

#### Actin purification and labeling

Actin was isolated from rabbit muscle acetone powder (Pel-Freez Biologicals) (Spudich and Watt, 1971), gel-filtered on a Superdex 200 column (#GE28-9893-35; Sigma-Aldrich), and stored as monomers at 4°C in G-buffer (5 mM Tris-HCl, pH 8.0; 0.2 mM ATP; 0.1 mM CaCl_2_; 0.5 mM DTT). Actin monomers were labeled at Cys-374 with pyrene-iodoacetamide (Kouyama and Mihashi, 1981). Pyrene-labeled actin monomers were mixed with unlabeled actin monomers to achieve 5% labeling.

#### Arp2/3 complex purification

The *S. cerevisiae* Arp2/3 complex was purified from commercially purchased baker’s yeast (Kastalia, LESAFFRE France) as described in Antkowiak et al. (2019).

#### Las17 purification

*S. cerevisiae* Las17 (375-Cter), containing the PRR and C-terminal WCA domain, was expressed and purified from Rosetta 2(DE3)pLysS cells as described in Boiero Sanders et al. (2022).

#### Dip1, Ldb17, and Ldb17-Δtail purification

The genes of Dip1 or Ldb17 and its variant were amplified from the *S. cerevisiae* genome or assembled from the exon fragments of the *S. pombe* genome and cloned into a pCoofy6 vector (a gift from Sabine Suppmann [Fraunhofer ITMP-IIP, Penzberg, Germany], plasmid #43990; Addgene) using a ligation-independent cloning approach (Scholz et al., 2013). Proteins were expressed in Rosetta 2(DE3)pLysS cells in Autoinduction LB media (#AIMLB0210; Formedium) overnight at 20°C. Cells were lysed by sonication in lysis/wash buffer (20 mM Tris-HCl, pH 8.0; 140 mM NaCl; 1 mM DTT; 40 mM imidazole; 1 mM PMSF; cOmplete EDTA-free protease inhibitor tablets [Roche]) 6 times for 20 s on ice. Lysates were clarified by centrifugation, and soluble fractions were filtered through a 0.45-µm filter and loaded on a 5-ml HisTrap HP column (#17524802; Cytiva) using an ÄKTA pure system (Cytiva). The affinity column was washed with the lysis/wash buffer and eluted with an elution buffer (20 mM Tris-HCl, pH 8.0; 140 mM NaCl; 1 mM DTT; 250 mM imidazole). Peaked fractions were pooled, and 30 µg/ml of Senp2 protease was added. The solution was dialyzed overnight at 4°C against dialysis/gel filtration buffer (20 mM Tris-HCl, pH 8.0; 100 mM NaCl; 1 mM DTT), filtered through a 0.22-µm filter, and concentrated to 500 μl–2 ml in an Amicon Ultra concentrator. The sample was loaded onto a Superdex 200 Increase 10/300 GL column (#28990944; Cytiva), previously equilibrated with the dialysis/gel filtration buffer. Peak fractions were pooled together, concentrated in an Amicon Ultra concentrator, flash-frozen in liquid nitrogen, and stored at −80°C.

#### Mass analysis by SDS-PAGE

SDS-PAGE analyses were performed classically, using 16 μl of protein solution at 1 µM mixed with 4 μl of Laemmli buffer. After heating at 95°C for 5 min, the solution was loaded onto a precast 10% polyacrylamide gel (4561035; Bio-Rad). The run was performed at 120 mA for 30–60 min. The PageRuler ladder was ordered from Thermo Fisher Scientific (product code 26616).

#### Protein mass determination by MALDI-TOF mass spectrometry

Analysis of protein mass was performed by MALDI-TOF mass spectrometry at the “Mass spectrometry core facility” of the University of Geneva. The samples, containing 1 µM of proteins, were purified and desalted using C4 ZipTip (Merck Millipore, France). The columns were first conditioned by aspirating and dispensing three times 15 μl of 100% acetonitrile containing 0.1% trifluoroacetic acid (TFA). Next, 20 μl of the protein solution was loaded onto the column, and salts were removed by washing with 0.1% TFA using similar pipetting steps. The bound protein was then eluted by aspirating 2 μl of the MALDI matrix solution consisting of 20 mg/ml sinapinic acid in 50% acetonitrile with 0.1% TFA. The eluted sample was spotted on a Bruker MTP target plate, dried, and analyzed using a MALDI-TOF neofleX mass spectrometer (Bruker, USA) in linear positive mode, scanning the mass range from 30 to 200 kDa. Baseline and smoothing algorithms were applied to the data using FlexAnalysis software (Bruker, USA), and curves were then plotted using GraphPad Prism software.

#### Mass photometry

Mass photometry (MP) measurements were performed as described by Wu and Piszczek (2021). Each mass photogram was recorded for 60 s on glass coverslips using the “TwoMP” device and “Acquire MP” software (Refeyn). Briefly, protein solutions at 0.2 µM were equilibrated at room temperature for 10 min prior to measurement. For each measurement, a 19 μl drop of buffer at the desired pH was deposited on the glass coverslips. After focusing the microscope, 1 μl of protein was added on the top of the drop. Homogenization of the drop was performed by three cycles of 10 μl back-and-forth pipetting within the droplet. Following mixing, photogram acquisition was directly initiated. Data analysis and figure design were performed using “Dynamic MP” software with a bandwidth of 5.7 kDa. Mass calibration was conducted using Refeyn “MassFerence P1” calibrant solution, yielding an R^2^ value of 0.999. Values are given in Table S4.

#### Shd1 and Sla1 SH3 domain purification

Sla1 and Shd1 SH3 domain DNA sequences with an N-terminal SUMO3 tag were synthesized at GenScript and integrated into a pET28a(+) vector for recombinant protein expression. A protocol similar to that used for the purification of Ldb17 variants was followed for the affinity purification, with the exception that the lysis/wash buffer contained 20 mM Hepes, pH 7.5; 150 mM NaCl; 1 mM DTT; 30 mM imidazole; 1 mM PMSF; and cOmplete EDTA-free protease inhibitor tablets from Roche, that the elution buffer contained 20 mM Hepes, pH 7.5; 150 mM NaCl; 1 mM DTT; and 300 mM imidazole, and that the dialysis/gel filtration buffer contained 20 mM Hepes, pH 7.5; 150 mM NaCl; and 1 mM DTT. The purified proteins were dialyzed overnight against dialysis buffer 2 (20 mM Hepes, pH 7.5; 75 mM NaCl; and 1 mM DTT) and loaded onto a HiTrap Q HP anion exchange column (#17115401; Cytiva). The proteins were eluted with the ion exchange elution buffer (20 mM Hepes, pH 7.5; 500 mM NaCl; and 1 mM DTT), and peak fractions were flash-frozen in liquid nitrogen and stored at −80°C.

### Pyrene actin polymerization assays

Pyrene-labeled Ca-ATP-actin monomers were incubated for 5 min on ice in the presence of 0.2 mM EGTA and an 11-fold molar excess of MgCl_2_ over actin. Actin polymerization was induced by the addition of 1/10 volume of 10× KMEI (1× contains 50 mM KCl; 1 mM MgCl2; 1 mM EGTA; and 10 mM imidazole–HCl, pH 7.0). Changes in pyrene fluorescence over time were recorded on a Safas Xenius XC spectrofluorimeter with an excitation at 365 nm and a detection at 407 nm. All experiments were reproduced at least three times. The curves presented in Fig. 2 F, Fig. 8 A, and Fig. 9 I come from the same experimental dataset. The curves presented in Fig. 3 A and Fig. 9 J come from the same experimental dataset.

### Phylogenetic, sequence alignment analysis

Orthologies were mined with PSI-BLAST and blastp at https://www.ncbi.nlm.nih.gov/. WDS core and tail regions were defined using MUSCLE and COBALT alignment tools. Proline-rich regions were identified by examining sequences upstream and downstream of the WDS core regions. Structures were visualized in PyMOL.

The stochastic character mapping analysis in the presence or absence of a C-terminal tail in WDS homologs was performed with the simmap function in R package phytools v2.4.4 (Revell, 2024). We used the all-rates-different model and simulated 10000 character maps on a phylogenetic tree obtained from Li et al. (2021).

#### Microscopy

#### Sample preparation

For *S. cerevisiae,* strains were grown overnight at 24°C in 5 ml synthetic complete medium without L-tryptophan (SC-TRP) culture in a shaker. Then, they were diluted to OD_600_ = 0.2 and grown until OD_600_ reached between 0.4 and 0.8. Ahead of time, coverslips were prepared by placing 50 μl of concanavalin IV (ConA, Sigma-Aldrich, 4 mg/ml in H2O) on the coverslip for 20 min, washing three times with 40 μl SC-TRP, and dropping 40 μl of cell culture on the ConA-coated coverslip for 20 min. Then, the cell drop was washed three times and a final 40 μl drop of SC-TRP was put on the surface. For drug-treated cell imaging, the cells were washed and then incubated with 40 μl SC-TRP containing 0.2 mM LatA (Enzo), or CK-666 (Sigma-Aldrich) or DMSO (Sigma-Aldrich) solution for 15 min. The cells were imaged within 30 min after drug treatment.

In S. *pombe* and *U. maydis,* strains were grown overnight at 24°C in 10 ml EMM in a shaker. The day after, they were diluted to OD_600_ = 0.02 in 10 ml EMM for *S. pombe* and 0.01 for *U. maydis* and put back overnight at 24°C while shaking. The next day, strains were imaged at OD_600_ = 0.4–0.8. Then, 200 μl of heated agarose with 2% EMM is pressed between two glass microscope slides separated by two thin spacers about 0.5 mm thick. While drying, 200 μl of cell culture is spun 1 min at 900 rcf and resuspended in 5–7 μl of the culture. Then, a 1 μl drop is put on the EMM 2% agarose, and a coverslip is added and sealed by pouring Valap on the edges. For drug-treated cell imaging, the 5–7 μl pelleted cells from a log phase culture were incubated with 40 μl EMM containing 0.2 mM LatA, or CK-666 or DMSO solution for 15 min before being pipetted on the 2% EMM agarose pad that contained the corresponding drug concentration to avoid diffusion. The cells were imaged within 30 min after this incubation period.

#### Data acquisition

Wide-field images were acquired with an Olympus IX81 wide-field microscope equipped with a 100×/NA1.45 objective and an ORCA-ER CCD camera (Hamamatsu). The illumination source used was an X-CITE 120 PC (EXFO) metal halide lamp. The excitation and emission light when imaging EGFP-, mNG-, and mCh-tagged proteins were filtered through the U-MGFPHQ and U-MRFPHQ filter sets (Olympus). The microscope was controlled using Micromanager (Edelstein et al., 2014).

TIRF images were acquired on an Olympus IX83 wide-field microscope equipped with a 150×/NA1.45 objective and an ImageEM X2 EM-CCD camera (Hamamatsu) and controlled by VisiView software (Visitron Systems). 488- and 561-nm lasers were used for the excitation of EGFP-, mNG-, or mCh-tagged proteins. Excitation and emission were filtered using a TRF89902 405/488/561/647-nm quad-band filter set (Chroma). Laser angles were controlled by iLas2 (Roper Scientific).

#### Data analysis

General image analysis was performed using the Fiji distribution of ImageJ (Scholz et al., 2013; Rueden et al., 2017). All display images were corrected for background fluorescence using the rolling ball algorithm of ImageJ, and movies were corrected for photobleaching using a custom ImageJ macro. For particle detection and tracking, the Particle Tracker (Sbalzarini and Koumoutsakos, 2005) of the MosaicSuite was used. Average trajectories were obtained as described previously (Picco et al., 2015). Plots and statistical analyses were computed using R. The statistical test used is the Wilcoxon–Mann–Whitney test for the pairwise comparison: ns, P >0.05; *, P ≤0.05; **, P ≤0.01; ***, P ≤0.001; and ****, P ≤0.0001. Statistical tests and values are given in Table S5.

Patch lifetimes: Lifetimes were measured from dividing yeasts and computed for each strain from at least three different fields of view. The number of frames from the appearance to the disappearance of a patch was counted and multiplied by the time interval between frames measured in the metadata.

Number of endocytic events: We counted the number of sites showing the colocalization of a coat marker (Sla1) with an actin marker (fimbrin) followed by their internalization. This sequence of events illustrates a successful endocytic event per cell per minute, which is the same as the rate of initiation of actin patches.

Tracking and fluorescence intensity profile: A method based on the averaging of subpixel centroid positions was used (Picco and Kaksonen, 2017) (https://apicco.github.io/trajectory_alignment/). For each protein tracked, the centroid trajectory is recorded for multiple endocytic events and at least 35 trajectories are averaged. This method also provides the fluorescence intensity over time. Fluorescent intensity profiles of Las17-EGFP are aligned using the alignment of the peak of Sac6-mCh using a python script (Picco et al., 2025).

Patch fluorescence intensity: Cell cytoplasm was subtracted from the original image using the Fiji median filter. Fluorescence intensity of patches from at least three fields of view was recorded using the circle tool of Fiji with a 10-pixel radius. Fluorescence intensity from the background around the patch was also recorded. After averaging the patch fluorescence intensity value and the background fluorescence intensity value, the background value was used to normalize the patch value.

The plots were rendered using the ggplot package in R or Python 3.11.

No animals or human subjects were used in this study.

### Online supplemental material

Fig. S1 demonstrates protein purification quality of WDS proteins. Fig. S2 shows sequence, structure, and evolutionary comparisons of WDS proteins. Videos 1 and 2 provide real-time videos corresponding to the still images and kymograph representations shown in the main figures. Supplemental text lists FASTA sequences used for evolutionary comparison. Table S1 shows strains used in the study. Table S2 shows plasmids used in the study. Table S3 shows medium compositions. Table S4 shows mass photometry values. Table S5 contains statistics values for all comparison possibilities of data shown in graphs in the main figures of the text. Data S1 shows the text file that lists WDS protein accession numbers and sequences, with features highlighted in indicated colors.

## Supplementary Material

Table S1 shows yeast strains.

Table S2 shows plasmids.

Table S3 shows yeast media and buffers.

Table S4 shows protein molecular masses

Table S5 shows statistics table.

Data S1 shows text file that lists WDS protein accession numbers and sequences, with features highlighted in indicated colors.

SourceData FS1 is the source file for Fig. S1.

## Data Availability

The data are included in the article and the online supplemental material.
