# Supplementary material for: Evolutionarily diverged on-switch for actin assembly in fungal endocytosis
Source: J Cell Biol. 2026 Oct 28;226(1):e202506135. doi: 10.1083/jcb.202506135 (PMC13715591; doi:10.1083/jcb.202506135)
Supplement: Supplementary file 1 [file jcb_202506135_sourcedatafs1.pdf]

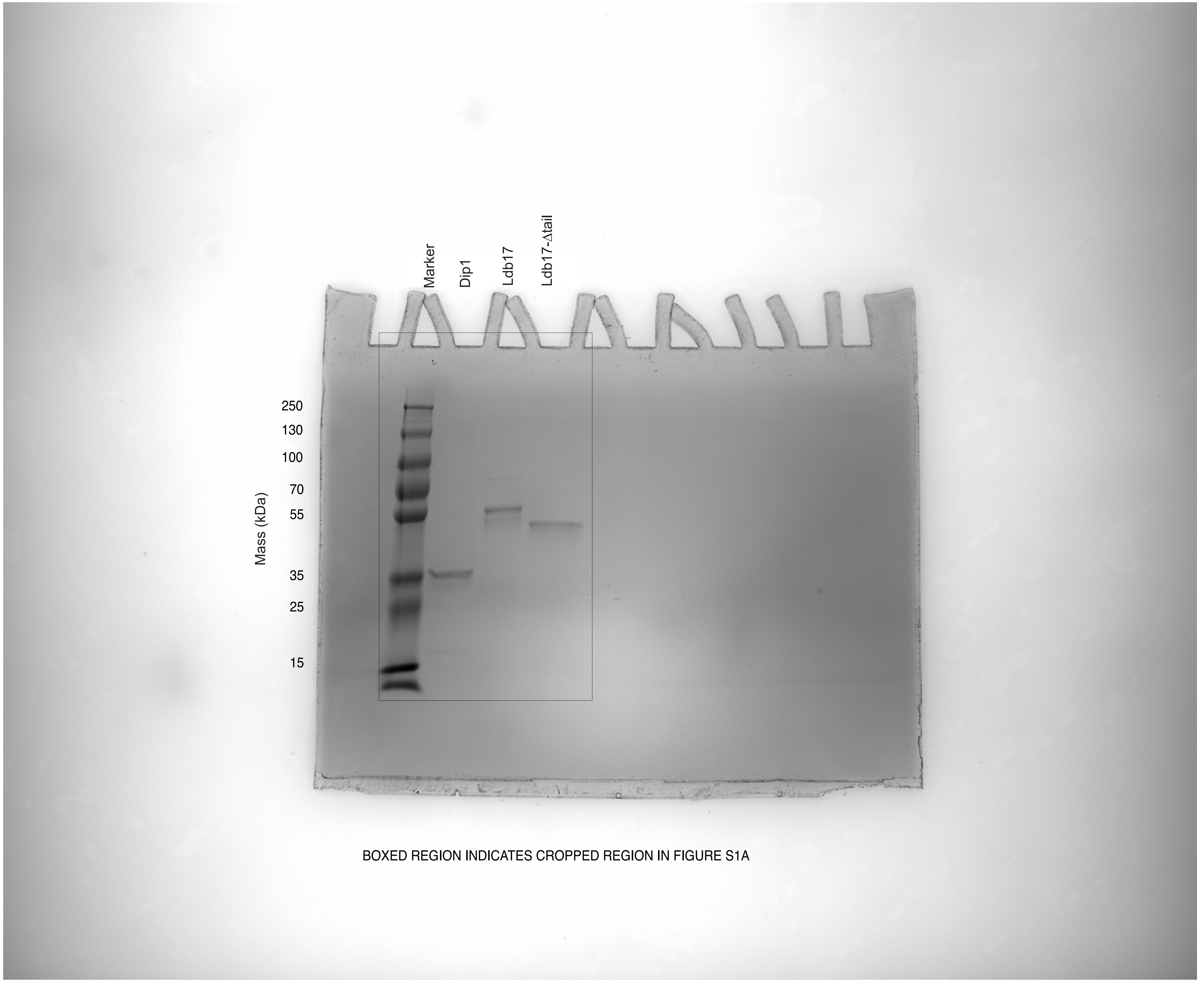

BOXED REGION INDICATES CROPPED REGION IN FIGURE S1A
