# Supplementary material for: Evolutionarily diverged on-switch for actin assembly in fungal endocytosis
Source: J Cell Biol. 2026 Oct 28;226(1):e202506135. doi: 10.1083/jcb.202506135 (PMC13715591; doi:10.1083/jcb.202506135)
Supplement: Supplementary file 2 [file jcb_202506135_datas1.docx]

**SUPPLEMENTAL TEXT**

WDS protein sequences:

- Proline rich regions in red
- Flanking positively charged residues in blue
- SH3 domains in green

>sp|Q12342.1|LDB17_YEAST RecName: Full=Protein LDB17; AltName: Full=Low dye-binding protein 17

MILDPLSPNIENHTQDEIIEFWEKTESIANIPKENLDESHVNSSLVAYLKFATDSYKVFINTDRDLYRMS

LILLESSLFEFKKEFCLSKLQSLLNIDLLEMNMKFIIVYILLCEAKKNVYSLEIMLKFQGFTVFYNTLYT

QFAYLSKYGKERTVASKHQYNSNNSSTGTSLDSLDRSLTDIDLGIIDEMKQISTVLMDLLFQIMKYCKCV

IANLQIVDDFFVYYMMESMRSDTMDDMFNNAEFKLLLALNEQYMMFAKEYDIENKVYKYLINGSVSRCFT

ELLLLKFNRASDPPLQIMMCKIIYLILTPRGDYSPMNFFYTNDLRVLIDVLIRELQNISEDEEVLRNTLL

RVLIPLLKNTQLSKTHYRKDDLNKLLNYLSTLDNICVDSPALHEHQVTVALSRKCLQQIPWLETPSTPSD

GGSSVSSNNTSRNSSIVALGTPDNQNILARKGHLYSNRELDVSAESLT**KRK**A**K**A**PPPPPPPPP**SRKCGTP

K

>XP_457077.2 DEHA2B02508p [Debaryomyces hansenii CBS767]

MYDSFASYPHNYYTKSDDETEIPPTTFPGENLLTKDHLLEQLQPILIISDPSECNNNLSLFMKLIVENIY

DQNEETTHNYQKLSSYALKLLTSNLFIKNYELCLGKILGLLAALSRDTLCSSEGIDEDVKFEIESLREFI

CIILLLLLKLTNSPGEKSERSKILETVDRNELYEILSDLGIIPIIANVITNHIVTTDKSKSPFLLLKFGG

DIIFEYLYNCELLSDVEFNSLTSETNLIPTIIKHLLTNDDFDNYDTDADDWEGENKLFIYEEFKLLLLIN

EQYLMKSYSSKESKNKVFDGLMMGDAHLSSENSQPSNKQINGFINLLIYYINREESQIIKILILKFLYLI

FTTSYTAKLFYLNDLKILLDIIIRELNNLDYSGNANGILIVTYLKVLYPLLMFSQLSELPEGYKNEGILE

ILRNLVLNSESGNRKTTEISEATDENQADIIAKLSLRCMSIPWLRKPQLSKRKNQDNPNLINNVYGKLSA

ASSSSSLNSKSSFVGKRSELYESSDISSESLGKAFTRIASVRTSVRSDYHKHALVHNENDQVGPNGSKSS

YVENNHNIFLDNDFKSLSINTTEDFANSPWIPISDESNLLDLPKEYLKDEPVQPLKRPESASSSIKSDSS

LAQKASKKKAPPPPLPPKSFSPKPNHIHFEKRSQSQT**PPPPPPPPPPRRRR**LLHLCEH

>NP_984171.2 ADR075Wp [Eremothecium gossypii ATCC 10895]

MALKPPYSPESGGASRSRTQTETVHFWEYLESLLDAADCTSEAQVNSALVAYVKTASERYGEYLVEDRDF

YRVALLLLRAPLYERNKSFCISKMLSLLSIDLLEMSMKFVISYILLCECKADSSSLDHVLDYQGFTVIYN

KLYEHFAYLHRYSDDEETTFEANITELDEEINEELRKISTVLLDLLFQILKYSKCELANLQRVDDFFVYY

MMVSLRSDTVEDMYNNAKFRLLLALNEQYMISNHKAGLENKVYAYLMNHTASKQFVELLLLQFNRTVDKS

LQIMMCKIIYLILTSRDEIAMDYFYLNDLHVVTDVLIRNLTNISEDEEVLRNTFLRILDLILRKTEWTQS

HYREQELLELLEYLCSVDNLCSSGNVKSEHMSTTKLAFKCRQAIRQLKVEQKKSRDAAAAPAPRPKTPEP

RFERTDSHMSVPMDKITRAIGRSPFAS**PPPPPP**S**RR**V

>QEU59741.1 Ldb17 [Kluyveromyces lactis]

MATSMTIQDVTLVEFWEKLEELLAEVNVRNESEVNSALVSFVKITADHYSSLIKCDQDLYRIGLMLVESP

MFQQTTDFCLRKLLSLLSIDLLELNLKLIIAYIILYKCKVDLNALDIILENQGFLVLYNNIYSNFAYLNK

YGDEYTGVPYQANEYHDKAQEDPKKDTNLEIIEDIKHLTTILIDIMYQFFKYSKCQISNIQMIDDFFIYY

LVSSIRSDVVDDILNNVKFKLLLALNEQYMIFQYEYDIENKAYEFLLNHNMSKDFIGLLLLKFNRETDTS

LLIMICKVIYIILTKDLKVAKDFFYLNDLHVFVDVLLRNLNNISDNEEILRNTYLRVLTPLLNNTELATT

RYHRSEISATLKFFSDVDNFCSIEKATPLQKTTARLAAKCLHEVKWLEQENQFSFDQNDSEESRRSSISQ

KSTSNTPLEPVMSNAPHLYSNLQNSYSADSINHKSNQQT**PPPPPP**S**RK**V**R**H**R**QGSDRKLAIAKV

>XP_018187492.1 hypothetical protein L228DRAFT_262045 [Xylona heveae TC161]

MEFEVSYSLDNEQQFWDELDEIVSAHCQSHALIDNALRSYLSFTTNYKDEYLQSEYDIARCSYKLLRSNL

FQTHKEYVRRQIVYGLLQEDDANMLHVITSFLLFDGQQDEKTFEMMSQEGAFPRLLELAKNANLNEDTGL

HRMLLELLYEMSRIQRLRPDELMLVDDHFISYLFQIIEELANDVNDPYHGPVIKVLLVLNEQYMLSAHDP

QASDIHGVLLTNRVIKVLSLRGGCFKTFGENLILLLNRESETSLQLLILKLLYLLFTTRTTYEYFYTNDL

YVLVDVIIRNILDLPEESSALRHTYLRVLHPLLAHTQLRDPPHYKRQEILRLLSILTTRRSAHFAPIDDT

TARLVGRVASVSWLANETRDSGEVARRLLRIHEGPAGESSLSVAEVASQQEKPGVLTPSRDPTAKGRAAS

KEEEIIIASEHGIVHSTEAVSAGKSPFEVEGEISA

>XP_019027561.1 uncharacterized protein SAICODRAFT_68774 [Saitoella complicata NRRL Y-17804]

MDVLYLSIDDEQQFFAELDDIVNSPIHSRDQVDNTVTSFVRFAAQFREQYLQTTHAVCRVSFKLMDSSLY

EPNASHIRKLLIRLALLPECASSMLYMILALLFLDGRTHPKTYRAMNKHGMFGKLIETVKHGEDAQLQRM

GLELLYEMCRVQKVGRRELESIDVEFLVYLFSFVEDMSDEHDIHSYAVLKVILVLNEQYMVASLPSQKDQ

VSAFDIASPAPSKPGTPLPPLSNKVMELLSVRGSEFKAFGENIIFLLNRERETCLQLLVLKFLYLTFTTP

ETYEYLYTNDLRVLVDVFIRELYDLPDEAQSLRHTYLRVLYPLLSHTQLRHAPHYKADQLRKLLLCLNGT

ARSAHFQAVDATTIRLVSRCAKVSWLEMPQEVKVQEKELDELSKKMERLGASLGRKESGSATSVAAVAVV

QKPPPGKVTP**RK**M**PPPPPP**G**RR**H**R**NGQCTPTIAVE

>XP_502138.1 YALI0C22462p [Yarrowia lipolytica CLIB122]

METLLESLMSSVSDEPLLAPDISIPPEEDGDMSDSDFWTKLLDIVSVKTHSYPVVESALKDYLLFATKHL

SDFLVSDNDLFRAGYKLTTSPIFTLHKAFTRRKLIAMLTLAEEYTDLAAVVSLILLIDSDSHPATLEMMQ

EENACHALTRLLLTFQKTSALRLRRVLLELTFEMCKIQRISTSDLENISDKFIDSLLRTVVNADRDDTSL

YDASVMKVLLCLNEQYMIAAYDEDEESGIYSSKVLDLISLDIDGYVEFGAKLVLLLNRAIDTCLQIMGLK

LLYLLFTNEPTYEYFYTNDLLVLIDVFIRELHDLPNDEEQLINTYLRVLHPLMVNSQIRHEPYKIEGLTD

VLEMLSGHKGSSTFLPVSETTLRLAVRCLGVPWLEYTFPSPMSTVPPSPSSSIGSFNEEESPSKIRVPSI

GMTDFSGTRRLSQSSLGTNSAMSSTTSLISSLKLAPPPPPSRASSKGPPVPPPSRGKSPMPQISRV**K**S**P**A

**PPPPP**S**R**NSPAPKKDVKESRDKKDKKEKPRLPPSRKKLLDMSIRNTSVGDLSVAERE

>XP_002838491.1 uncharacterized protein GSTUM_00006270001 [Tuber melanosporum]

MDFDVAYSLENAQQFWDELDDIVSVHCETQNLIDQALKSYLAFTSSFTDEYLVSEYDWAKCTYKLLDSPL

FTKHRNYVRRRMIGRLRKEILTPRLHLVALILLYDGRGAQKTFEMMLEEGLFVRLMDLVQKTRDEDRMLW

RSLIELLYEMSRIQRLRREDLEAIDDGFISYLFGLVEEDPEDPNDPYHYPVIRILLVLNEQYMVSAHAPP

SNPADPPGAPTNKVIKILCFHGSTYKTFGENIILLLNRESETCLQLLILKLLYLLFTTSATNEYFYTNDL

RVLVDVMIRNLLDLPDESEPLRHTYLRVLYPLLEHTQLRHPPHYKRDEVLKLLECLSGVRSNHFQPVDQT

TIRLVSRCANVPWLRSAPAENVQPENPAHRALGMSLTNHGQSSVTLLEVASKSEKPGVITPSRKLGTGGA

NIDGSSGGNGVEIAGEA

>XP_001550569.1 Bcldb17 [Botrytis cinerea B05.10]

MDGFEVAYTLDNEQQFWDEIDDIVSSTCASHQLIDNALRSYLKFTTNFKDEYLQSEFEVAKCLHKLMQSE

LFAANKDYVRTQIVYSLMQEDVPATLHVLTSFLLFDGRNNETTFEMMNKEGCFPRLLELVKKGNREDPTL

HRLLLELLYEMSRMQRLSFEDLGQVDDEFIICLFEIIEELSDDVNDPYHYPVIRVLLVLNEQYMVASTST

TDPSHSLTNRVVKILSHKGSSYMTFGENIILLLNRETETSLQLLILKLLYLLFTTKATYEYFYTNDLRVL

LDVIIRNLLDLPNEFVSLRHTYLRVLYPLLAHTQLQQPPHYKKHEVVKVLSILGGSGNAHWEPADETTIR

LVERVAKVPWLRDDDISEGEVARKLLGISLSPSHTGSSISVSDVAAVREKPGVQTPSRKAESENAAHDHN

TPVVVPSDEIP**KKKR**AL**PP**L**PP**AV**R**AITAQ**KK**V**PP**MT**PPRRR**T**K**L**K**AMTTQ**PP**EATGVE

>XP_959600.2 hypothetical protein NCU02418 [Neurospora crassa OR74A]

MADLEVTEPVENEQQFWHALNSILSAPCPTPESLDDVLRSWLELVATGRDRYLDDEDDIATCSQQLRLSS

IFRQNADYVRTQIIYSLLQEDEYGPLHVISNFLLLDGRAEEETFRRMVNEGCFLRLLELIKSCGAHDGRL

HRLLLELMYEMSRIEKLPHQDLAQVEDEFVIYLFQLIEAFSDDVDDPYHYAVIRVLLVMNEQYMVASASG

AVDPTAPVTHITNRVVKVLSLHGPSFRTFGENIILLLNRATETSLQLIILKLLYLLFTTKATYEYFYTND

LRVLLDVIIRNLLDLPVEMNTLRHTYLRVLAPLLSHTQLSQPPHYKRDQILSLLEILRGSGSAHFLPPEP

TTLRLVERVANIPWLVDDEGVLSSVPMKSLTQAQNGSTVSVIANVHEKPGIETPSRKSNLARELRHEQED

AEDAVRSIIAVGPASDTPETTTSNLSVSSTTEGHKAAGRRKPSPPPARRLRAQKSLPIVPKHRHGVPVSP

QPPHTPTRMIENSTFAQGDKHGVAAASSSSPTAASAAEAVSGDG**K**VSGPVA**KK**Q**PP**KL**PPPRR**G**K**L**R**AVN

SLSTLHPVTESLPQTGSTPVA

>XP_661336.1 hypothetical protein [Aspergillus nidulans FGSC A4]

MELEVSVDNEQQFWTSMIVLPVFSALVVSVLRTDLFRIRAGIQEIVSAPCSTEDLIDDALRAYRALFCKY

YGPSHVLINFRTPPEQYLQSEFEVTTCSLTLVSSAIFVAHKDYVRRQMIYALLQEDDPVTLHMIASFLLF

DGRHHEIPLYMMNEEGAFPRLLELLQVQSLRMDDESAANLHRLLMDLLYELSRIQKIKIEDLILVDDDFV

RGLFDIIEHVSYDANDPYHYPVIRVLLVLNEQFMISAHNPDGEQSPLTPLTNKVIKVLSMHGNSYKTFGE

NIILLINREAETSLQLLTLKLLYLIFTTPSTYEYFFTNDLHVLVDILIRNLLDLPEEASALRHTYLRVLY

PLIAHTQLRLPPHYKRDELKRMLSLLVRGQLTGDAAADSEKIMHFDEVDETTRRLVLRCAAVDWLRDEPE

PHESSRPSQREDSSILSFPSIDSALLSDADIGTSLESSQSIGSNSTRESSLEDLSSPPGEDRDRSASADE

TPRKHNQIERLGMHLTPASSSSLSVDEIASQREKPGVITPSRHDGPVPRTQVSPTKHKVKPLPPKTRRSR

GRRATVDETASPISKTSISEERGNSSPSPFTTTPIITTSDQFSPDIERRDSSSTIHSQTHAL**P**V**PP**VQHQ

Q**PPP**TTN**K**TT**RR**SASN**PPP**AV**PPPRR**STHHHLHFSSHCTPVASLSPSRLPSQQGSITPTATGTQAQLPNQ

GQNQSHKQAQKPEPPRTRRSGRKHTFTDSSDTSLSSHSQGVNSISSCASNGSGSSGVATTFLHPDYQYQY

SHNNSSAPAPGFAAAAVGIVEREDGKTAEPESGANGHQLISVEEALQKVSLH

>TIA24060.1 hypothetical protein D6C80_00418 [Aureobasidium pullulans]

MTVRFCWAIEASEKMQPADHDDDQSGAFWQRVQTVVSTQCTTHEQIDDCLRSFLEASTDGFLDTEYDIAR

CCYRLIDSPLFLQNKDYVRRQFVYCLLQEEHAPVLHIVTAVLLYDGRSDEAVFEMMQTEGAFARLLDLVQ

GNKDDNTGLHRLLLELLCDMSRIQRLSLDDLATVDDAFVIYLFELIEELSNDANDPYHYPTIRVLLILNE

QYMCYAVSPESHPTLTNRIIKVLSNHGPAYRTFGENLILLLNRESALGPQLLILKLLYLLFTTPPTFEYF

YTNDLHVLVDVIIRNLLDLDPGSDDEDHPDVSKDGGGQRALRHTYLRVLCPLLKNTQLAHENAHYKREEL

RKLFCLLVNRSSFHFSPVDETVVRLVSRCRQVAWIRDESDHEDPVSPPTDAHVAKRLLGMNLAEAGESSL

SVLDVSA**K**VE**K**E**KP**MV**P**A**PRRRKR**LQQQQSKETVLLRVSPNNAQNGERSPFDDDNNEE

>ANZ74406.1 BA75_01238T0 [Komagataella pastoris]

MLDVPDQLNQDQFWNLITEILCTLQNEYVNGMPEEVVNSLLVQCITLAIHCENKKARFYEKSSKFLIDCD

WFINNLEFCTRKLLSLLQYTNDNNTRIIQLLQKNDDLTYKDCLVQNIKLVEHIGWILYYLHDQEALSKFQ

WFEILSSTLLNFYIIKSHISLDYELEFSNYLLISIDYCSSLDVEQLHSLPDRLLGNLFSSLLLVSRNEDY

ENFLQFKLILALNEQFMIQSHELVYDNDVQSSRCENKVFLFMIQDDNFSNFVECLMLHFNREDDHLLQIL

ILKVFYLIFTTSMTCKWIYLNDLKVLVDIFIRELLNLSITNEAPLINTYLRVLYPMLCFTQLSEVCYKLS

NIKEALQNLISNESCSKTMKRLTERCLDLKLFSEESDDQESIVSNSTTSSFRQ**PPP**I**P**LA**RK**FQ**K**SIYIT

DNNSSFDLASSKVSRTPQ

>XP_003716356.1 uncharacterized protein MGG_03559 [Pyricularia oryzae 70-15]

MADADAGQSGSVPVEGEEQFWQEMENILSAKCSSFESIDDVLRSWLYLVASCRELYLSTEDDVAACSQRL

RDSDVFGCNSEYVRTQIIYSLLQEDEAGPLHVIANFLLLDGRSDEVMLRRMIDDGCFPRLVELINGTRDN

DRRLHRLLLELVYEMSRIERISSEDLMQVTDEFVQYMFQVIESLSDDVDDPYHYPIIRVLLVLNEQYMVA

STMRSNEPPLTNRVIKVLSMRGPDFRTFGENIILLLNRETETSLQLLILKLLYLLFTTKATYEYFYTNDL

RVLLDVIIRNLLDLPNESSALRHTYLRVLYPLLAHTQLSNPPHYKREELIRVVRILGGAENAHFAPADDT

TLRLVDRVSKVKWLGLEPKELSPVGSGESEVARKLIGISLSESQIGSSVSVSDVAAVMEKPGVQTPSRRA

DHALGAKKPLMASDANTTKPDEETAKAVTKPKRVRPEVPKHRHGKLLVPAQPVTSSLVNSLCESADESGT

DSCTSTTTKTHSN**KK**V**PPK**A**PPPRR**TT**R**L**K**QAASAANLRGPETTSVS

>KAI5817470.1 hypothetical protein BZA77DRAFT_34321 [Pyronema omphalodes]

MNHGIPYVTGPVDPRMQAHLYGGYVQPVMNQGIQQYYDAYGNIQYYQQQYYPQPAYVYPQQVYDQQVYEQ

QVYEQQVYEQTPQQLEPEPELDWREVLRRTVDYKCETVQTFDTHFKAYLREFSLLFTEKKINEDDLDQCA

LILQRSPAFSAIQTRPGDQYKQASNEAQIQNHLVERLSKAADDLQLLLMLKVLRVCGKTDEQTAGKMRTM

FKAMAVSEVAFKCVTERIMNQPYDSVMRLTCLRLCHEMIRPIIVLKKLLTTYVTMEFLHHLLSVIVEYSW

DEDESEYGELDPYHNAFRIITSLSEQYLVQGCPLEENPIHKNLQIDYMQPKRSFSKFGEKIFYILNRERD

HRSVCQILMLLHSLIGSPIKEDQVLILYRNDLMVLIGILLRYLNDNDEEEGYVRVHEYSIRLLEDLKNNT

DLCDSDSVRMDGNGGQNQRDQIYYTLKRIIDHSSGATLRLAKRCWQVWFEEKDTLLSPYGNPQHATSATS

FGNVDKNW

>CCG82560.1 Putative uncharacterized protein [Taphrina deformans PYCC 5710]

MDHDISYEVEDEAQFWQEVDDIIQPTSTTPSAIQNVVNNFISFVSQFGADYLTTDADVEICCEKLLDSDF

HMDNAELVREAMIAYQPEQDKIAKCYVNLYLLLLDGRKNTRTFKLMKRAHMVPVIANWAKQHQVDSIRFT

RLALTTLYEMCRMQKLSEADMHCFDEEFIWFLLRSVEISRDDDDPYSYNCIKVIVSLNEQFMVAGLPQAQ

QPFPRTSSNTSLSVASIETTGPHQAQNSIVKVLSDHGSRFKAFGENLIRMLNRERDTALQLLILKQLFLL

FTNAATYEYFYTNDLHVLIDVFIRELYNLPTEDEALRHTYLRVLHPLLIHTQLRHAPFYKATEMVNLLTS

LNQECSHFGPVGTTTVRLLARCLTVPWLLPPRKRKLKIPRPGFTAERKKSAKNLGMSLDQSETASIASFH

SVNSIA**K**V**RPP**TT**P**RI**KPP**V**K**D**K**GNASARLEQSHLAPVTEGVTTASNGGQGKSAMVDSVELSAQATEKHD

DLMREIGKLEISA

>XP_019027561.1 uncharacterized protein SAICODRAFT_68774 [Saitoella complicata NRRL Y-17804]

MDVLYLSIDDEQQFFAELDDIVNSPIHSRDQVDNTVTSFVRFAAQFREQYLQTTHAVCRVSFKLMDSSLY

EPNASHIRKLLIRLALLPECASSMLYMILALLFLDGRTHPKTYRAMNKHGMFGKLIETVKHGEDAQLQRM

GLELLYEMCRVQKVGRRELESIDVEFLVYLFSFVEDMSDEHDIHSYAVLKVILVLNEQYMVASLPSQKDQ

VSAFDIASPAPSKPGTPLPPLSNKVMELLSVRGSEFKAFGENIIFLLNRERETCLQLLVLKFLYLTFTTP

ETYEYLYTNDLRVLVDVFIRELYDLPDEAQSLRHTYLRVLYPLLSHTQLRHAPHYKADQLRKLLLCLNGT

ARSAHFQAVDATTIRLVSRCAKVSWLEMPQEVKVQEKELDELSKKMERLGASLGRKESGSATSVAAVAVV

QKPPPGKVTP**RK**M**PPPPPP**G**RR**H**R**NGQCTPTIAVE

>XP_018225623.1 hypothetical protein T552_02180 [Pneumocystis carinii B80]

MEVYNTNNIRNTIRDVIVNKYLKKESDYEACCWKFLNSDMFISNKDYTRYQLIIMINQEDKLKYFYILYW

LLFFDARTYPQTYKLIKDKGSFQSLINTTWKRFQEFPELHKLGLKLLCEICKVHHLTSDELFFIHEEFLC

FIFELIEKNTCYEDPYTLSAIKLILVINEQYIIKSLPSHLQNSSSDASRNIENESPVNLVMNILKKKGDI

YKTFGENIIFILNREKDVWLQFLILRQLYLLFTTKETLEYFYTNDLKVLVDVFIQKLYNLPENNKKLYHA

YLRVFYYLLKNTQLKNPPYYKQDQLQKLFKTLKDVIEYQHNKSANSITEKLLLRCIKISWIRNDELENNI

LFSTNWSNKSDSSFIEVSTNNDCIFSNSNRV

>XP_002173439.1 cytoskeletal protein [Schizosaccharomyces japonicus yFS275]

MEISDVLYQIDDKKQFLSEVDEIVNQRVSSKEQLNRLVDNLIRFICAYGVSFLTSEEHLLDCCLQIRNSA

TFKDNVDVFRSALLATLFASENIFGSDFVLYFFLVDASKDPACLHFLADMGFFPLLIDHIVRRIKLNKTN

EESSCPYGLCLLVRLCRTQRISMPDLKSVSDFFMEALFLEVDLAKENEIEDHFILCILAILSLNEQFMLA

GLRTNEDPGSVNGHANKVMAALMNPNIDTRSYGEGLVFTLNRENDVRVQLLILKQLFLIFTTPETYEYFY

TNDLHVLMDVCIRELNNLSPESSALQYAYLQVLYPMLVNSQVRDPPHYKTVDLVHTLQAIVEPNPMSSRT

IPPATEKAVEQVLSIPWLQQAN

>NP_001342760.1 WISH/DIP/SPIN90 ortholog Dip1 [Schizosaccharomyces pombe]

MEFTDVFYNLENEKQFLAEIDELLTIPYEKNELEIGVSKFLSFINKYGEPYLITEFQLQRCMEKFLNSPL

YQLDENAVLSIFYDCFFFLKEQQTLKFIIIVFQSEIQENEYCLRLLASTGFIPVLIKAMKQFKDRSENVA

FTSFRYALFLVYYICRSRRLSPTDLVAIDEYFLVNLFKTSEEAWNDEDMDSFGMCVLTLLSINEQFMLVR

LASKGSFEIANGIMDLLSSSKVDNGIYSEGLVFTLNREKDPRSRMLILKQLFLLFTTPATYEIFYTNDLN

VLIDIFIREINNIPDELSDLRYAYLQVLIPLLENTQVRHPPHYKTKCIVDAVNNVLISHSKSSNMEDART

TDVAIRVLEVPWLQQEMKSLGTAQ

>KAE8204400.1 hypothetical protein CF328_g1110 [Tilletia controversa]

MDPFAGYAAIQSQQHFWQELDEILDEPSLHAGQARSGHGSIASPARIGIHSALQRFLALIDACYDRYLDH

EYNRTYTVKRLLDSALFSEANEEHASECVVDQLRTATSLRKVLICYDIILLYGQRNPAIFRLIQSRIKDI

LFRLVHQIWAGFYATQAEERLTADEAASKGSSKVGQSLHFEYARRAASQGAATCDALPPQPGWTGADWDP

RTSQAPRVPLDAAAIKSAEVLVGKPSSASIMRQLQIDLRSKAVRMMYEVCRVQKLNDVEMRPLDLKFVDH

LFDLVERTREHDDDTFNYQLIKLIAALNEQFMVAGFAGGESTTTKNTVLEVFKTRLNVSYTFGENLIFML

NRASSDAEDICMQLLVLKLLYLLFTTKDTAHYFYTNDLKVLVDVFIRELSDLPDESESLRHTYLRVLHPL

LTNTQLCTVPYKRPQIRRLLLALVSQEHIRDVSATTKRLVDRCLRAEWCVALDELTQGT**P**QAGL**PP**TIIA

QSSV**P**VR**P**TSAAQRT**P**HAGL**P**T**PP**IAQSSV**P**VR**P**TSAGHLRANNISLQ**P**MSAQTLA**P**SAV**R**T**PPP**IN**R**AT

**K**GTRSPAGTNSPKAQQTSFDRQIRLPATDHY

>XP_036635736.1 uncharacterized protein PC9H_000229 [Pleurotus ostreatus]

MTLTNTSYAPAISLEDEFGIVYLVENAQQFWSELEDLLNFPKDEIPTLSLLDSTLKRYLALCAAYHEQYL

QSPLQLEHACQLLLDSELFAFHSERMCEILVEDTRSSTDPHAQFIFFNVLLAFGRRRADFFRSHKRWQPL

VPLLMDHVLVEIDPDTEDTYTGSQQTPMVVPIEAKLRSLSVRLLYEVCRVQKLSVQDLRIFDDAFIDYLF

DLVEQTRDMSDDSFNYSVIKLIVSLNEQFMVASLSAQSSELVDPNADPAQPSTIKNRVLRVLMRRLNSTK

TFGENMIFMLNRAERTPEGLCMQLLVLKILYLLFTTHGTSEYFYTNDLCVLVDVFLRELVDLDEESESLR

HTYLRVLHPLLTKTQLRFTPYKRPQIMRCLESLISNTSFRDINSTTKRLVERCLGGEWCVQLKSQSPPPQ

SPSTSSLFSDPGASDGRSSGSESSTPQGHAHSSSGAGYHSSSGPASKTLKSSKSVEHMHSKSSPALKSPL

SSTPSYSIVRPGLENLRRPSNASSISLHGVASAVVSSAAKASAVHPSRRPGTVDSVMPEHAHHVSHNHPS

QHDTAEDGSRSDSGAASHANGSGLPATSTV**K**Q**RR**TA**PPPP**A**PPKRRKPP**AV**P**VG**R**TNGGAVITAIKSSAP

TSPLAR

>OJT03655.1 Protein dip1 [Trametes pubescens]

MDHIRLDVDPDLDDGFYGAGTESNSGAEHAIEVPIEAKLRSLSVRLLYEVCRVQKMSIPDLRIFDNDFID

YLYDLVEQTRSMQDESFNYSVIKLLVALNEQFMVASLQPHTPLSQSGLAQQQADSRSPEHANRVLRVLML

RASTSPTFGENLIFMLNRANRTAEDLCMQLLVLKLLYLLFTTKGMAEYFYTNDLCVLVDVFLREIGDIDE

ENESLRHTYLRVLHPLLTKTQLRNMPYKRPQIVRTLENLVENESIRDIDPTTKRLVERCLSGEWCVQFRK

SATARTAEQRKFSMWDADIQRGDSPGLDAVSSAPAPQSAGPLSGAGGMKPQLDRMGSLRGKNGKASRSAE

HLPLPAASSASSSAQKSRPSQPSHPHSRADEPPYKVSARAVSVHAVRHGSGDSGGSLPRVAAATTHAAAK

HRFRAGSFDVEGTTTSSFSALSLQTDPLAVYHLLDDAVPYVDEPAGGSPPEIRVLSPTSPGQPGPGLLSP

TDRAFAASAPSSASSSSTSLVSPSASSPQ**RR**AA**PPPP**V**KKRRKPP**AVPVGRGASPLSALAASSQPNLAVL

SAGARKASGL

>XP_011387282.1 uncharacterized protein UMAG_01405 [Ustilago maydis 521]

MDPFASYAALDNPQQFWLELDEILHTPFHPASYASTCCASSNDQAEKLLQAKRIYVASVLAKFLHLCAAS

FNDNLTTEYNQDYCINRLFNSTVFRDDVEPTSEAAAAAAAASSSSSVASANNVAMHQLAAEEAIRIMQTS

TSIPVLLMTYEIVLQYGDAFPSTYKSIQASASTVSFVGVRAFLHRLVHQIWAGSYAAQAEAKIGISDKQH

SDVGAWNADHWDQDEPVSAVISKASTKPNSSSASKDIAIQISLREKAVRMLYEVCRVQKLEASHLKAFDE

RFIHHLFDLVEETRYHHNEDFNYRLIKLLVAMNEQYMVSALASTQTHSSTPCSSRPVNLVLSVLQQRLNA

SKTFGENLIFMLNRASSSDAEDVCMQLLVLKLLYLLFTTKETACYFYTNDLRVLVDIFVRELNDLPDESE

SLRHTYLRVLHPLLTNTQLCTYPYKRPQIRRLLRGLISHGHLNDISATTKRLVERCLRAEWCVELDRLDG

TESVAKLEPIGGGEAHVKLMHVGLTSEGLPMLDTRIKERSSEVQATADSLAVTTEDQVANPISITTTMSP

ADATSTDVASVSASFASSAPPTASLNVAASLRTRDKRVASLSHAPCEAAPASALLRPNSAASFSRTTSTS

KQHNSTSGSGSGNGNGNGNGNRRAVSAQSAPCTPPRMDSPFLAVGRRPTGRLYEQSRSLMGVNADADADT

DDALSSSSSWHAHMLEEPGSESGKHQAGQAGQAGQAGGRVSEPDANAMHSAHAHVQSGLNSDLTEQLGGV

HYAGGSVDGGMGAPSSASVAGGAGRRQRRKPPSPPQARRVLIQSHSAELDVARSRPASAAECAHTTTSHV

PASLGAAKAGA**RRRPPPPPP**VASSGSAVATASTGHTRANVEHEHRRTDDMDNAQSSYIAERLGRGLRIR

>CDR88119.1 related to LDB17-protein involved in the regulation of endocytosis [Sporisorium scitamineum]

MDPFAAYEALDNPQQFWAELDDILDTPFQPPSSSHHDSILQAKRIYVASVLAKFLHLCAASFNDNLTSEY

NQDYCINRLFSSTAFRDDVDLTAGESQEDIAMHQLAAEEAIRIMQTSTSIPALLMTYEIVLQYGDAFPST

YKSIQASASTVSFVGVRAFLHRLVHQIWAGSYAAQAEAKIGISDQAQQHSDVVGWNGARWDQDEHSSDQP

QTDTTSASYTSRGLTSSSASKDIAVQISLREKAVRMLYEVCRVQKLEPTHLKAFDERFIHHLFDLVEETR

HHHNEDFNYRLIKLLVAMNEQYMVSTLVSSAPAHSSATPGQINSAPPSASRPVNFVLSVLQQRLNASKTF

GENLIFMLNRASSSDAEDVCMQLLVLKLLYLLFTTKETACYFYTNDLRVLVDIFIRELHDLPDESESLRH

TYLRVLHPLLTNTQLCTYPYKRPQIRRLLKGLIAHGHLNDISSTTKRLVERCLRAEWCVELDRLDGTERV

AKVEPIGGGEAHVKVMHAGVTTEGLHMLAAKIKENSDSVQATANSLAITTEDEAVSPISISTTLSPTSYS

TESTASSDLPQSGSLSVPATLRTRDKRVASLSHPRTTSEEAASTLLRPTSAASLSRGTVKQYSNSSSSSN

NSKHSVNGTTTTTTSKRAVSAQSAPCTPPRVGSPLLHVASSLRQTSRRTELSSPVVGDGGDTDDALSTSS

SWHAHMLDDPPAASGKHLRRGGEGSEAVLTGGQEAARVGSPLSSKSTMQILDARVGHVADGSDSTITGVG

VGTSSSGE**PPKRRKPP**Q**PP**AQARRAMQLSSSEPLSRSASPYAVSSTDSSPAHEDMPPGVQGGSVGRSAG**R**

**RRPPPPPP**TAAAAVATSPAGNPEEQDELANAQSFYIAERLRRGLRIR

>RPD60621.1 hypothetical protein L227DRAFT_586071 [Lentinus tigrinus ALCF2SS1-6]

MPAPPMDVFGIVYIIENAQQFWSELEEILHLPPDVTLSKLDSALRGFISFCAAYHEQYLQSPLQLDHACD

MLLASELFQFHSERMCELMVGDAQTTTDPHYETILYSVMLAHGRRKASFLRSQKRWQPLLPLLMDHVRLD

VDPEVDSGFYGTGPGSSSGSRSIAVPIEAKLRSLSVQLLYEVCRVQKMSLQELRLFDDSFIDYLFDLVEQ

TRDMQDETFNYSVIKLIVALNEQFMVASLHPNTPQPNPNGFAAQQVDPRMPESSNRVLRVLISRVGSSPT

FGENLIFMLNRAERTPEDLCMQLLVLKLLYLLFTTKGMAEFFYTNDLCVLVDVFLREICNIDEENESLRH

TYLRVLHPLLTKTQLRNMPYKRPQIVQTLESLIENETIRDVDPTTKRLVTRCMSGDWCVQFRKANPATPT

NRKFTIYEEEIRRGDSPGLDAVSNAGAPHSAGPLGPQQAQLGRMDSVRGKHAKASRSAEHLPLAPSGASA

PKHRAVSGHHSAHRSSDAHARKDSISRVRQLSGDSSTSLPRVAEASTAVSAKHRYRAGSFNVDATTTSFS

ALSTNPRSNGETIHILDASSPLTEEPPEIRVLSPTSPGQLTSSPLDRSLSSSASSSTSSLDTI**P**K**P**AS**P**S

G**RR**AA**PPPP**VKT**RRKPP**AVPSRKGQLSPSGGSPLSAIAASSQPNLPALSAGKASAY

>CEH14403.1 Coeffector of mDia Rho GTPase, regulates actin polymerization and cell adhesion turnover [Ceraceosorus bombacis]

MEPFVASYDVTRLSTPQLFWSELDEILYADLEGPVASSSKTQYPARISKSKVPKVISGFLRLADACYGRY

LEQTYNLDHAIARLFDSPAFSDHHEYAAELVVNLVRNARTIPSLLIGYEVILQYGQSEPIIFKHLQGSAR

DLLPRLKHQIWAGHYAAQADQSLASGLTTNHGVMQDHEAGWSGASWDKSDSDRYETEYTLPNARLNNAND

SATSWRRWQLELRKKAVRLLYEVCRVQKLDHRDMRAIDAKLVDHLFDLVEDTRLHADEEFNYLLIKVIVA

LNEQFMVSALALATEAIRDEKPSKDTPQRSGNLIISILKERFNHSKTFGENLIFMLNRASSSDAEDLCMQ

LLVLKLLYLLFTMQETAHYFYTNDLKVLVDIFIRELSDLPDESESLRHTYLRVLHPLLTCTQLLSHPYKR

TEIRRLLLGLISHAHLRDITPTTSRLVARCLQAEWCVELDALDPSNAAAIKQLVGRHDSSSSMTEELADA

GVAIASVQSIAGGPASQVLVETTIEDGEPVIAPDASASTSTGADAMAIAVAVEDGGIHSSLVGSAGRDFE

ESSLFTSADVKANESGRERVSSISQHIEVTPWNGTATSTPVPSSPQTTFTPDFASGVVASGSETDAAMQS

SWHAQALPSISLLSEHSAPRASSRGPGPPRPPKSRGASSQATAAERTASSTDLPVNVQGGTGIATHEDDL

KPNAPEPSSDNALSAGSVDGLPDTADGLVYSFPLVRVQTSEPSTEAKQTQSEMLPFRPPSLHVQHASGEG

DVDEDSTHRTPTLSDLSSRDRMHSSSNAASGSSTPQRRKPPAPPRGTNGVAYLGVGMGRPRRGSMPNSPI

QSRSELAPSDYLQPSDPASQTFAPSSSDAAEARQPDSAPANSLPRSFMRERDHIPRASSFADLSARAREQ

**PPP**ADMDEAAEGARSSSSNGLYPSGKV**RRRPPPPP**SSASNDNSPNLMAVSGSYPLAKTSETTASSHL**KPK**

**PPP**INRAT**K**GTAS**PRP**AT**P**LVDDKSRDDSRHDSDRNMSTGQLSGRLQQLRCAH

>XP_014656675.1 conserved hypothetical protein [Moesziomyces antarcticus]

MDPFASYAALDNPTTFWAELDDILDTPFHPPPPPPSHDTKSSYDAALLDAKRIYVASVLAKFLHLCAASF

SDNLASEHTQDFCINRLFASTAFRDDVVPETGAIPMHQLAAEEAIRIMQTSTSIPVLLMSYEIVLLYGDA

FPATYKSIQASASTVSFVGVRAFLHRLVHQIWAGSYAAQAEAKIGISQHAATGWNGAHWDEDYQPAPPAA

AASTAPPSGSASKDIAIQISLRQKAVRMLYEVCRVQKLEPGHLKAFDERFIHHLFDLVEETRHHHDEDFN

YRLIKLLVAMNEQYMVSTLASAPASGAALAGGADQINSAPPTTRPVNLVLSVLQQRLNASKTFGENLIFM

LNRASSSDAEDVCMQLLVLKLLYLLFTTRETACYFYTNDLRVLVDIFIRELHDLPDESESLRHTYLRVLH

PLLTNTQLCTYAYKRPQIRRLLNGLLAHAHLRDISTTTRRLVERCLRAEWCVELDRLDGTSVAMVEPVGG

GDVHAKKMHAGVTSEGLPMLATKIENNTQVRATADSLAVTTEDEVASPVSITTTYSPDAAPASADRPRTS

GSLSVPPPLRTRDKRVASLSSPRPCDDGAALLRTNSAAPASSSSGNAGRRAVSAQSAPCTPPRTDSPSLG

ARQNSRLSELSTDDDALSTSSSWHAHMTEETHTHEASDSTAPKRRKPPQPPSARRLQLSSSEVYSAPSSR

ADSPCAASPASEDAGEPLAP**K**AGA**RRRPPPPPP**TAAAAAAAAAVVVPTVTADDELEAAQSFYIAERLRRG

LRIR

>XP_050199582.1 uncharacterized protein SCHCODRAFT_02314501 [Schizophyllum commune H4-8]

MSVPTIAMEDDLGIVYVVENAQQFWSELQDVLHFPKDMIPTLSQLDLALKRSLALCAAYHEQYLQTPMQI

EHACDLILDSELFTFHSERMCDILMSDIQSTTDPHMQLIFYFLLLHYGRRTRHATGTRAEFFRNQKKWQP

VIPLLMDHIQVDIEEDAFDIDEDADFDQSSASGSSAVGGVPVPIEAKLRSLSVKLLYEVCRVQRLSLSDL

RIFPSAFIDFLFDLVEQTRDMRNDAFNYSIIKLIVALNEQFMVASLQASHSPSPAGSPAPDQEHHRKSNH

RLSNDPEGKNQNRVLSVLMRRLGTSKTFGENVIFMLNRSKRTPEDLTTQLLLLKILYLLFTTKGTEDFFY

TNDLFVLVDVFLRELGDLMDVDETDATHGSVLESLRHTYLRVLHPLLTKTELREVPYKRAQLAYLLESIV

NSDGRGATKEVGPTTRRLAERCLGGEWASGLRTQRKKAASDFGPHAAVSPTTELTRPTPPRLTSKSVENL

SSTMAGITIAPPPPLPTPSSAFFLSSASSQSVPARSDRSHAHRYQQAAPHSSISIPSGLKGIERATLSPM

SISSASSVADSDASASTVERPH**RR**AA**P**A**PPKKRRKPP**AI**P**ANAGLERTSSGAYVQPIKPGLKSAH

>XP_014567837.1 hypothetical protein L969DRAFT_49264 [Mixia osmundae IAM 14324]

MEFGATYFLDTAEQFYSELEDMLTVPRDTHLGELDYALRHYLSFCSHYYHDYLQSDESLERAADVLADSS

LVQGHRDRVVDGLIDFVIKTRSATGLHIALVSLHLLAAQAPHIYRMVHQARYPPSNPSRPTQQIGPNTPL

TTNLCDILLRYGHAYVDDAYPDAKGATSPDERDRSANDKICIEAAGLLYEACRVHKLSIIELGIFDESFI

KGVFQLIESTRNYGETLSYTLIRLIIALNEQYMVALAASKPNGDAKRSKKETSLDADANLVLRIMRRQLG

SSKTFGENLIFILNRLDHSSESLCVALLILKILYLLFTTRGTHDYFYTNDLCVLVDVFIRELYDLPDDQE

SLRHTYLRVLYPLVTNTQLRSYPYKRAEIRVALLSLVCHANIRDVNATTKRLVDRNLKAEWCLDLEGRTV

NGGTMKRDYPAESAREIEAQPYDQPSLVNGLQSLELNTTSSRTSTTSLTAIGAATSESSSVSGSPWLNSP

QEMSPEAHFLKPVGAPHHRPASSLDHHRKILGSSIERSRSAPHSPDPSSHSSAVNTKPVFHK**RRP**A**PP**A**P**

NVR**K**HAPIPSAIEHVTAPARQDSLNVNPFV

>XP_047813111.1 hypothetical protein Pst134EA_000736 [Puccinia striiformis f. sp. tritici]

MWAISELFILSSLSASHQSQAGLVGFDYLLDRTRIRTDSRKYRPVGVLVMASETDQQFWPVFNELLSVDP

TGNLAELDGALIQSLEMIGSDLAGYLSDEESLHHAVSTLLHSPLFLNHSQRVTNSIILILIDPSTPLSLI

FVACLVLLLNGFENAIFFRTLRTINLSALSETNSSSPISHPSPSAELSQLIPVLLAIGTRVGLVPSSFID

TLSNHSHLNLYPPDSSPDLSSTGSANERYAERKLGPLLSGLLYELCRVQKLEDATLELFSEQLIDNLFQL

VELTRDQEDETFNYNLIKLIIALNEQFMLAGLNPQSQQQIHNAPSHHHHRRKGPRTSDVESNIIIRTMKH

RLDESKTFGENLIFILNRASHSTSEGLCVSLLILKILYLLFTTPGTHEYFYTNDLCVLVDIFIRELSDLP

DESNDLRHTYLRVLHPLLTQTQLRSYHYKREEVRHVLLSHIKYAHLQDINATTKRLVERNLHSDWCLDLE

RTARSLNRPGLAGLSCERSLSVETLGSQLSGGGSMAEVITSNTDSIPITQPSSSTVLLVTHHQLPPSSSP

AKLQAQTPAIHNPSYTTPQGLRSSVVLNNLSHAFVQVTIADSSDCYEETQAIHPIASSSYPPEMINRPAS

IISARSISPNSDGSLRPSSSCSVSRSPARRPAPKPPTSKRKGPLGTHLSPDPNSEGPSTCGSEIPIVDEP

EPETSRRASIPSPDSLLNPSTSSQKHRSHPIDLTKHIRRAAPTPPQLSSPSSYPDETPPIQIISTNAA**RR**

**RK**A**P**L**PPK**EAFSKLRSSKSSSHL**P**LT**P**TLL**P**SAS**P**SSSSSHTAWKPSHTRVVSLSSDQ**P**I**PP**ANH**P**SYRD

**KR**DNN**P**FGE

>XP_016274079.1 hypothetical protein RHTO_07830 [Rhodotorula toruloides NP11]

MEAQQEWHFSTAEQFWQDSSLQELDYCLRNYIAFASSFMDQFLAEPGALDHALLALLSSPLFTDHADRMT

DNIVSSIVHPDASSATLFISLMLVLHLGEGANPLTTAGVWANPQKPGAGVTVGSSKVFRRMRKRWNEVVP

VLMKWVCDAGVVEERHTGELSEPEKRDDQQVGMPAEGWEERVGTAATAVLYEMCRVQKLTLDELSDFTFD

FISHLFSLVERTRDAEDETFNYSLIKLIIALNEQFMVCTVPAAPKGSGQLPPPILPTVVGKTKRERGPNT

VLEVLKEKEHESKTFGENIIFILNRADGTPDSLCVSLLILKILYLLFTTSGTQEYFYTNDLCVLVDVFIR

ELYNLGEESEGLKHTYLRVLHPLLTNTQLRHYPYKRQELRRVLECLVAPSYYRDCDPTTRRLVERNLRGS

WCQGLRSAEAVAAGRTLGSDSATGSTLSVDAVARADDEAVRKHKRKHSHRKSRRQTSLDETVSSGSYGNA

GVTIADGWTVLQPLAERAVTGQAPEVVRSSYATYTSTSPERESRFPSTSPALDYELPPSIPSISAGHDIL

DPVARRLMAGSPRSSTQPTSGSLAPPLVHPRPRSTSLSATSYRSSTHGLGLSES**PPPP**L**PP**S**P**AMSSASL

SASVGDLPSACATP**RRRRPPPPP**TDGGS**RPR**T**P**ASQTLLPQSTGGSDGSGPPSPHTTTITSG**RRRPPPPP**

TQAGRASPRVHGGFEEDTRRRLEGAAIS

>SCV73775.1 BQ2448_6205 [Microbotryum intermedium]

MSSLSELDSSLRNYIALVTKYIDEYLASPGLFDNAIFCLLEAPFFSSHLDRMAANVISTISPEASYSSLF

ISLMIILHLGLTDPLAASTMILRSDPTRSGTARVFRAMRARWQGVVPILLKAVWDAGVVEHDHAMSKPYG

AEAETSLGSHQRWEVRLGTAATAVLYEICRVQKLEAFELAIFSYDFIDHLFALVERTRDVEDETFNYTLI

KLIIALNEQFMVASLSSQVQSNGGSVALPPPILPQIVGDRKPERGPNLVLEALKARTSESKTFGENIIFI

LNRSNGTPDSLCVSLLILKLLYLLFTTAGMQEYFYTNDLCVLVDVFIRELCDLDEESEGLKHTYLRVLHP

LLSNTQLRHHPYKRPQLLRVLLSLVTNSSYREVDPTTRRLVERNLRGSWCDGLKRDGDRPKSDASHNPHG

RAQRIAHSHRLDDARGGLLIANSSSISVDAVAQAQDAGGTSRNRLAGHRKPNESTTTPLQSTELVADAPR

SWQAHPSALLALPETLQPTSQDHLRSLPTARSDSHGSLSSH**RRRK**A**PPPP**SERSNAATFETCMS**P**IT**P**TC

Q**RR**AA**P**G**PP**VKFGT**PP**VL**P**VG**P**A**PP**NQKH

>XP_012048938.1 hypothetical protein CNAG_01419 [Cryptococcus neoformans var. grubii H99]

MPHRDPQARARDRHHHHHHRHQPSTSRSALDTGIGILAEGSMHVHVKVHDVEQFWREIELVVTIPESPTL

DQLDNTLRMFVNFCAAYHDKYLPSQSDIQQAVTMLLDSELFSYYDERMVGIMMSDAQENTNPHDLYILYY

IILYYGKRHPSLFRSHRKWRKLLPTLGEVVGLDVEETFVLGLPPIETRIRLPATCLMYEVARVQKLTSSE

LAQFDDQFIDHLFDLVETTWDQQDERLNYAVIRLIVALNEQFMVATLPSKHHTRVHALVDKAGDRNHLGI

PTIPSSNGDGDGGHTRHHHRAKSGNTTQSGGYTHDHQEEAKKNNRVLVVLMRRLGSSKTFGENMIFMLNR

AENTPDDLCMQLLILKILYLLFTTPGTQEYFFTNDLKVLLDVFIRELVDLPEECEALRHTYLRVLYPLLN

HTQLRSDPYKRPQIKLVLRSLVANSHIREIDPTTVRLVERCLEEPRKLERSLSAENMQKALHHSSASTIS

LSALSSALPPSKSQLGTSIYTSRDPVRQSSLNDVSSSLTDVKSRPHSSASIASTCSTSTETGA**R**AT**PP**T**P**

T**RRRKPP**A**PPR**HAH**PR**AGACAVARKDSTTTNSSWTSFDSDAEASMSDSTSTGASVLGSSFGAGGRVMSPT

SPINGTDDLVREGGKGVPPPIIEVHPVGREQPMGWITFST

>TIA87727.1 hypothetical protein E3P99_03004 [Wallemia hederae]

MVENGKYSQLEAQPLTDAEEADDLVTDDEDDLPSESSPLLHDTKPVSRPRKPSTWAVAVWASSLVICLFT

LLFVLLAKCDTDALAGRTTAIPLSDSALRNSVAFDLPSDLEFEQNQPVVLKTTVPFGLDFDQALGVDGLG

AGGIVDPNGGLLQRWRLAIGRMLSRSVSPLDVIVNHPITVEARSSKQTIQLFNVTLINSVSVPITAGLQN

SNSHIAHPQGWLKDVNLKASITPVDGVDFDLEGLQSFFEHVWVAEKGMMNVLVTVPQVEVHIWNKSILHT

HFQKNDIRLQESLAIPDLAPWPKPGTPLEETYMNMLKLLNITQLDIGTHNSTDKKEQIEVDASASLPLPQ

LDILKSIESVVIPYEIPVEAELPLRKEEGGGSLALATVTSKPIEIADDELRFGGKASTGLQWKALKHSPY

IKTALSRFLSNFLNNTPTPFTIRGGVANENAPKWLKKVLSRFGYNFDLPGVPPMPDIVRNVHMDNLSVHR

QSGFPWPNPFPGQPGGPGLVLVSGTLIADIALPAQLRSLPIDVNSILPNLLLFNGSPPPLNSTRDQVYPR

NAFAIFHPDEAFDARTELADDPEDSDAPQLTRLYAQVRDAPIKLIEGRDSVFRSFMGKMLFGKAMVGVDG

DATVNGGLKGVKDLDSIDIKDIRVNGEFSAADGINQDYLFGNHNYGDLPLKPDHVNRPLWVSPEDGHIIL

EGFSPIAEQAQDFLVAISEPVSRTSLMEEYKITPYSLYAAVSVGLETNDIIEVLNRLSKVPVPASIISFI

KECTMSYGKVKLVLKHNRYFVESAHADTLQMLLRDAVIKNARVDSNANLETSSAPTKGNLIIPGTKEAHT

GGNTNEQNQPTEQAEQNEQNDGNKDGNKDAGDDYFTALIDIEKQDEMEDDDEVHSFEIKPDEIETVKRRC

NELEYPMLEEYDFHNDTRNPNLDIDLKPVTVIRPYQEKSLSKMFGNGRARSGIIVLPCGAGKTLVGITAA

CTIKKSILVLCTSSVSVMQWKQQFMQWSNVNDSQISVFTADQKEKFGGESGIVVSTYSMVANTRNRSHES

QKMMDFLTSREWGFILLDEVHVVPAAMFRRVVTTIKAHSKLGLTATLVREDDKIDDLNFLIGPKLYEANW

MDLAAKGHIANVQCAEVWCPMVPCFYREYLREKSRKRMLLYSMNPHKFQACQFLIDFHEDRGDKIIVFSD

CVYSLEQYARKLKKPYIHGGTSQVERMRVLQRFQFDSNVNTIFLSKVGDTSIDLPEATCLIQISSHFGSR

RQEAQRLGRILRAKRRNDEGFNAFFYSLVSTDTQEMFYSTKRQGFLVDQGYAFKVITHLFGIERLPDLVY

RSEKEQIELLESVLLANESAADIGSDIKASEDDLAGTIHGVTKRMTGSTSALAGGQAMSYIEKNKSANKQ

IAKDSRHALFKKRDKDAADRAKKKRQSIEMKVVRSIIAQGEVLGSGSYSSVKLAIHIDTGDKYAVKVINK

KMMEGREDLIRNEIAVLKRASTGHSNIVRLVDYFETMNNLYLVMDLCTGGELFDKICQRGHFFEKDAKDI

VYVIADSLRYLHSVGIVHRDLKPENLLFRDDKSDASDLCVADFGLSKIIESGRFTQLYQICGTVGYMAPE

VFKRTGHGFPVDIWAIGVITFFLLSGTTPFDRNNNQKEEINAILDGNYSFEPVEYWSKVSDTAKDFVRGC

MNTDPTKRLTAQQLLEHPWLKGLGEKDEDLLPNVRRAFDAKRSFRAAVRSVQWINRLKTMPVDTSDEERE

EYRQQVFALQQEAEKEELVPGLTTLYTYPFILYLEYNCCLRPMTFDDEDFIVWEISDVKDFWAELSEYGH

APDDATVLELDGRLKMFIKLSTYLIDTGQLDTALAMMIESDLFRFHYMRMQTVLIDLIIHTTDPHQLFVA

YSLLLSYGQRRIDYFKCPTNWRKLMPLSLDVIRHVEDGYFGQDDDGTGLVPIEVRLRYPVICFLYEISRA

QKLHQQDLLAFDSAFLDHLFDLVEWTRDESDECFNYTLVKLLTSLNEQYMIRNIETSSSSSHSQHLQISN

NLVLNVLTRRLGSSKTFGENLIFILNRASDSKEDVCVKLLVLKILFLLFTTVGTQDYFYTNDLKVLVDVF

IRELSDLPDEQEGLRHTYLRVLYPLLNETAVKDCQYKTLEIHATLTASIASKVHDVSATTSRLVRRCLSS

ECFDGIESEDESVSAFQPANEPTTPNMVALANDFTHPTPHLRATQSASDLLRTWSPNASTTNLPTISSDL

DVLRQSFNNVDIQSPISAPASTTEMHRTIADFAPSPVSSQSSLSTT**RRK**A**PPPPPP**S**R**LN**KP**A**KRP**SLS

>XP_007005340.1 uncharacterized protein TREMEDRAFT_39577 [Tremella mesenterica DSM 1558]

MDVHIRVHTADRFWREIEAILVVPDPPTLIQLDNTLRMFVTFCAAYHAKYLPTTQDMQNAMSLLLDSELF

TYHYERMVGIMMADAQENTNPHDLYIIYHLILYYGQRHPSLFKSHRKWRKLLPTLGEVVGLDHDEASNLS

TYHSHTYVLGLPPIESRLRLPATNLMYEVCRVQKLDAQELAQFDDTFIDHLFDLVEVTRDQQDERLNYAV

IKLLIALNEQFMVATLPPKPHKATLSAPLVSTATLPEPEDYSRPTQSTSEEAPTSGLLDTGAQVRHHHRA

HSAEPGPYTHDHSEEAKKGNRVLVVLMRRLGSSKTFGENIIFILNRAENTPEGLSMQLLILKILYLLFTT

PGTQEYFYTNDLRVLLDVFIRELVDLPAECEALRHTYLRVLYPLLNHTQLRNDPYKRAQIKLVLKSLISN

GHIQEVGSTTLRLVERCLEEPKHLERSISAKDLRQDSTSSTFSLDSVTAALPHPLPSKPSLLNTSVYTSR

DPVRQSSLVDVSESLANGQRPNSALSSRSDVLSDATS**PPRRRKPP**A**PPRKK**FSVASSTSTDGESRMGTPM

MSHGMLTSDQTGETDKSEGENGRVYGTVPIIQVHPAQPSCPIPGWITFG

>XP_027485042.1 uncharacterized protein MRET_2546 [Malassezia restricta]

MTSPPLPTFAQDAAYISNVLERVDTQYVMTHLYDVASQRVFGRQCVDMVCGSAKPTHLDLRAEPSAGMAR

VLDVLHARTQTLSPAELLIVYVIWIALGEYNINAYRWMLRRDTMHVYIHRLVQNIWAGHYAAQTSASLVH

DTDVAFEPALPLPPDHHRLHVDARSDTPSDSRRTRHVLICVERHAMHILYECLCSVRLSSHDMRGITRPF

VDHVLSVMETHESHEQSAICMSVMLALHEQCMMSTNAASLLSHIQHRLHTSKPFGENVVYLLNRTPSTTF

DGCRFHILVLKLLGAIFTLRETASYFYVNDLKVLVDIFLRQLGDLPDAYDVLRQAYLCVLHALLTQTQLW

SVEYKRAHIVRLLTNLVRTASLHDMSERTLTLAQLCLDAEWCVGTDAGNTPLVACLHGEGGIRGASSSSH

DHDVIQYMWLLACLQPTAASQACMLGVDADTIPLLWSEYELEDEQEACMPLDFDVRTLESWSRQSSGDAP

PHIGNRRRAPPAPPARMPRLAHTLSSASMPELHAHSSSVDPPSPSAASGTVTPPLRRQAPAIPARETPPT

GLRAYVQRYWRGKHDDTRHP**RRR**HVTSHDDFDTPAP**RRR**A**PPPPP**S

>KAF9156563.1 hypothetical protein BG015_004180 [Linnemannia schmuckeri]

MDSATYHFDSPAQFFEELKDILERKDAYDSPAFQVNAYLSLMVHYQDKFLDESGQDLVYCCYNLFDSDVF

RRNTEVITCCIIDRAIESSDDEDMWITYHVLLHAGNESPIIYRWMFRNEFFSKLKDQIQHREGTRMQLIA

ITLMYEMCRIQTLKESDLALADETFLNYLLDLVEHTRTDDAEQLNYGSIKLLLVFNEQYMLHTSSRPYDT

KAGTTFAYSGNPLLTVLTDRLGRSATFGENLIFMLNRAEETALQMLILKVLYLLFTSPSLYEFFYTNDLH

VLVDVVIRELWDLPEEEESLRHAYLRVMGPLLTNTQLKRATYKRAEIVRLLRELGGGDLDSTLRKQLQEQ

QLREQLLEQQRALDAKYTYRDRESSSSHFARWTSDRSGCSSPILSSEVEEKKRGVGRSKLSMVECSSDSL

KVPGQDEAAETLRQHLEHQQELVSSPTDENPLQIEVPTVEYEANRQAYNNISRSSQRAASPTTQRLVERV

LREWLDNEMKNGAGTTGHGLSVRGVTDGHSTEAADGRIVIPVAQ

>RCH86163.1 hypothetical protein CU097_002653 [Rhizopus azygosporus]

MDDDAIEYYFDDIESFYSELDEILHRPYSTEQDVQRIITQYIRFIVRYSKDFLKGSTEIDQVAYKLIDSQ

ICLDYSTVVLSHIVHSHALVDQDYIPYAILLIAGRDEPRWMKYILMESIQAKRNRLFCKLIQEIKLGIWD

TNLDISSILFGLCFELCKLNTMEKEDLDLVDSPLIHHLLDLVQETRGDAQESFNYDVIHLIMVFNEQYMM

SGSNENLILDVLSQRIGTSDIFSANLIFMLNRSNDICIQMLILKLLYAIFTRQTLYEYFYTNDLYVLVDI

ILRELCGLGDTKEGQTLLDAYLRVLEPLLENTQLNRRPYKKEEIYRVLQQLIHPGMQRKAHSTTKRLVKR

IIEDWYDCCSSPATSSLGPSTPADDIPEDVLAKQVV

>KAF9309367.1 hypothetical protein BG003_009845 [Podila horticola]

MAFITYQFDSPAQFYAELKDVLEEGGFQADPEAQVDSYITLITQHQDEFLDNSGQDLAQCCYRLLDSDVF

QQNTLAVTNSIIERAIQSPVDQDMWVTYHILLYAGKEFPKVYNWMLRSEFFAKLKYQILQQEGSSLQPLA

VNLMYEMCRVQSLKPCDIALLDEPFLHYLLDLVERTRTDADENLNYGAIKLLLTFNEQFMLNMSNRADYT

PSGAKTTYSGNSLLTVLTDRPGASCTFGENLIFILNRAEETALQMLILKLLYLLFTSPTLYEFFYTNDLH

VLVDVVIRELWNLPEEEESLRHAYLRVMGPLLTNTQLKQATYKRAEIVRVLRELGGGDLDTTLRLQLHEE

QMREQQLEQHRAASAKLTYRDRESSSSPAIRWANNSACPSPVLAANSVKNNIRLPQSKLSTSSTSSLEVP

AGVYPNGTRLSILDSPEDERTVDFSSAV**PPP**IE**RR**DSQPRAASPTTQRLVERVLREWLDNEMKNGAGTTG

VGLSVRDVTDGHVSSNQRIEIRVAQ

>ORY48256.1 hypothetical protein BCR33DRAFT_714657 [Rhizoclosmatium globosum]

MASPKHLEALLQLMTDVNALLDQALNAESAIQAAVDALMANHTIISTGHEMDVLCEHIVRHSIIDSASDE

ALAELLFLLKGLLKDLNENLELGRVVLTLIYYLGIVYGANVIWIPAHPCCFGLLLASNSLSRCQLLFQVC

LLQEMSISELNEFTPELIVVLFDSVERTSHSDELEDYNYALIKLLLVINDQFMMKNHARNVIENQIIACL

SPRLFLGKSLGGNIIFMFNRADNSRLQRLIIRFLYIVFKTPETATFFYTNDLRVILDVVLRESRAVADED

EEIQQGYLNLLPALLHNSQLQNYKLDDVKTLLGELRRQDSGNQQSNSPGKDVPLPTTPLSAFFGNPSVKP

STRRVAERVFFECREILSK

>XP_016612574.1 uncharacterized protein SPPG_08825 [Spizellomyces punctatus DAOM BR117]

MNGEEQAIYCESLVKRVDRLLEDKDTPFADVVQGVIKCLQPAVPYLSTRNIQLLCAQVLSGRLEDDPDEV

VDLLGNYENSEDDAAYIVYSLLHHLGHAWPNAFRRLDDEWLARLKTVLLKRSGERCHQMAALLMCEICET

RELSLSDLETFDASFLNNLAEMIEQTGSAENEEENYTLVRALLAIHDQFAQKNFARSVIPNRVIEVLQKR

IDQSKTLSENFIFIFNREEDRKRRIIMARFLEIVLSSTSARNLWYTNDLGIILDVVIRETNNVDAEDEML

HHSYIRLLPLLLRHPNVTWSERRKQEVEKQLVGIRDGPFVKSSTRKCAERALMDILRP

>XP_006679530.1 uncharacterized protein BATDEDRAFT_35173 [Batrachochytrium dendrobatidis JAM81]

MTESISDAVSHLETLLQDLDHYSQDASLNAVTSIVSRIMADIKFIRDEYDMRIIAEHVLDFPAFQDLSEC

LGCISRHPESENRDLLYIQLSILFHFGLSTPNVFRRLVRDDWIPVLKDILLLPDLDVKVSHVTVLLFDHL

CQVHEMSLHELDIIDTQLLNYLCELVESSRNDNESFNYAVIRLLITLNGQLMKMRGSVATHDRFLRVLEQ

RLNHSKTLTENLIFVFNRADTPELQLSITILLIGIFQYTPTRNLFYTNDLTVILDVGIREIQRVHDQNEE

LLHALVKLLPLVVVSGASSTSHKQVVNVLNKLNESAFTKLPTKRLIQRVVAELSLYDGHDP

>RKP33147.1 hypothetical protein BJ085DRAFT_27740 [Dimargaris cristalligena]

MSTPSDNEDKTSDTPLTLQEFLVVSELLLVIVRSKHEVLMVDPLAAPDSLDAELGKHRTDMIAACLALLQ

CGWQDGSLINGLLDIVDSTCYDPNDEITNAAIRLLVALHHMLIQHDRTRLGILSDNLILGELARRPESSR

TFGECFVFLLNREGEPYPANVYNIAIKFMCVELLWSILQSPPLSNYFYDNDRNVLVDILLQNIADLADDT

DQVRALCLNTLGALICYTEYRQRPHKLSQVRRLLRELLNTGRVSSDFHLLARKVLGDCTEALAEASRHDA

PSRA

>PIA16533.1 hypothetical protein COEREDRAFT_8322 [Coemansia reversa NRRL 1564]

MEAPEQPTVGALLERKTENGTWSGNEAKSLAGATELPLRRAASSRFSELLDSSVDYQAYRQGCRQQVDLQ

PQFDTLRDAEEELRSVLHDKTFVQQCPDDKIAAENVALAVDWFGSALEQQFDGDEPFENADFLRFCCMFF

ASPLYENNARLVQRHVVKRTYAELSGTCGAELWVLLALLHLIREFQTDTLELCRDGGLFPLLQRLVISDG

ERRQHVLAMSLMFEIAQAVRLSASDVACVTEELLQFLLDYVERMRYAESEVYNNTATKLVLALDEQLHVK

PGPSPPLSYGPIGNIGDENMLALLAGSAQHSHRRRCSRRPNGTVFVQPMSPPSIYQQQQQPPQLLGRSGA

MRSHHTRAASTCLPAAGAATAVDDSTDINGYYPVPAQQEKLGDEQQLSRSRSTDLRMQVLPAYERDGQLE

LATEDSISHPSVVAMLAKRARGCKTFVENLVFLLNRETDPATQRLILGMLAGILTDADTAQILYTNDLNV

LTDIVIRELSNLSDTEQQQRRVYLQVVCALLRNPAYLAARHRLPDIELCLVTLLRQLLVSSQPASSSRRG

SLSDSSTRHSLTLSDLTPPVAGSTRSGAVSPAVSAASGETCCARPDHERHCSTLALKNM**RRPPPPPPP**VA

SRTHTPLPSPMDHT**RRRRPPPPPP**SPQSQNASRRPTPVSSPLRVSFSDARNQEPTDL**R**PS**RRRPPPPPP**S

S**R**PYSQQCTPAATKTTLHI**PPPP**L**PPR**TEA**RR**H**R**PEHTGIRRQLSVKRSVSRYKRNSTAHASTQ**PPKPP**S

**RR**FDHASTSAASISPLVEGTYEEENPTDKLPPSKHSDKHPLPANAIDAGGEASGSTDEDDDDTMQLESVE

DSLEERRASRRLVADALRACHEARILASSLALIQHKRLALIVHAIGREMRFRTLTGVQRHKLTNALFVAG

VVSAIGTVTGTNLLGCPAANRNKRLDNEIQEQTSSTCDPRELDGLVERRFFGGKLEPISKPKSS

>RKP13100.1 hypothetical protein BJ684DRAFT_20393 [Piptocephalis cylindrospora]

MLFSRLTGLTILIAALTLQPDFGHAALVSGEGSSARGGLGINASRPLRCVGGLKISAKRLKTEKGEEAKR

LCETIAVLKKRHAMARALQDRWVGIATEEIDRYSARELLHPGSAAEVARRENDWHTARLHARQAGVDMSG

SGPLHLNCGLGARLFTRPLDMECRRHGRCDFHAPRVGPCEGPPAVAPFLLDQLILAGSMEAVNIFADQLQ

TSTAFRRTDSSSGRRNRRPTEANSGAAEGDPFADSPLSPSASTSNSNSSFPIPDRDIFADDYFGYTDSSN

SGVSYNRNRADDNASIDEDPFMTQEPLQADPDPLIDSAMDVRLDSSDAHPARTPPSPIHDSPSPRGSTSR

NQPGRWTRHSRTLSQLHRDQQGQAQAKLEEHLDRILHRFPSSHEFSATLVQKTIRAFIDTLLYSTTTSTP

ASAPS**P**I**P**GS**P**S**PPP**DLLYLDPEMRASYCLGLLDSPAFSLPCIPYACAYLFDLAEEECETERRALVYELL

LATSQIYRDLLSQAHDRGMCTHWFRDILEIDDFWTQRLSVALLWQVLANRDIGPNDLDMVDDATVNRLLD

LVEMYGQDGDGPERPFVHQVLLLLARIDAASPGQDGGQRQLIRILTQRRRRARAFEENIISLFIHEPSLQ

ARSDLLTLFAGLFAQPETGTFFYTNDLSILIDVLIRDLWDLGEEETHVRIQTLMVLALILERTQWVQGRY

KHQEISRTLDMLGRVRDPSTAAKAKAVELILEWL

>PVU92704.1 hypothetical protein BB559_003628 [Furculomyces boomerangus]

MKFLYTITFILTILTTCYCGIADFGPEELVKMAPNVTIRTVVYKNEPTKLLSRAKNNLMFYVVNDSPMNI

TFNKFFGELTDPKDFSRVIEKLADFNVNKVIEPSAKGFLDYKFRVRTKNMDVMGVTVSVEFTDENGGFWM

TSPLNKTVQVTLDETEAKWEKEIYTTITYFVIEISNNPIFHNPETATQQDINNGIDWYINTLATEFQDEE

PWLEEEFINSCYSLFSSKMFLYYGTQVIERLLERTISSLAKLWVEFTLLALSGEKNKHLFVISKKHQLFS

RLTQLILESEDQTTKFTAINLACGLVQNGGIGLAEHRIVDETFIEYILCYVEETSAFMNEEFTTTATKLL

LSIDDYYYKNFRKLTLFKDSHMGRTALGNIGFGNSLKNKSTDLGNLGYLDGLNVPESPLQKILINKRMGM

CKGFAENIVFLFNRLEENLEAIEMTLMFLKRVFDSNILCTMFYINDLCVMVDIIVRELEAATEPDKKICQ

LYLLVIDSILKLDEYISCKHKIYDLELLLVSMLRKLLEDNSMSSDTIDENGKPKFYAEYNANTSHKGPEL

SSREELRSMSMRTESTQIYQMIATRKKVGKVAGSEQTLESDFGYNKQLHDLEFLVKKSLANPYPQTRNLN

PQSNPPSSTHQNRAVFPKSFKAINNLELGGDTGYSKSNLKSNFSSNTSQLPKQKVLSSSIISSSQSPSVI

PQQQQKPTNFNKEKPINVKQRPKSKSASKILPKSPFGNINMQEGGYQSVEQINSYFTKHRQETDLTKTQA

NLYSNQVLSPCSPQKITLPISQVLPISQKNKHKNVSPSVKDESHTKKSLINHGKSGSHNKTKSLGNYKIN

SNADFTNDLSGISSFEKLPSLESLNTYLESIKFDQKDLTSINKTIFSKSELKNPSKLHTSLLAFSNKNSL

NSPIEHKPDMPHDSPSFSYSASPTNKSSLKYPKKSHNNLKLFAETQTTETSLNIQTNDIKNKENGTHTIE

KTIYIGSVSHVPREKSSDLTIIQDESILPTGIETNSFSNENLQEKENKIEELDKNSQQENPNKQVLLDIK

DTRKLDVLKNEDTLQLKSKTIKDLENDNEIQEKLSIEKENTTETRQEEITKDIGTKEITKDIEKKKYVDK

IKKTPNISQNIDNKEKKVISENTETKSANDGISTTVSVDEMSKEKEIDLDRTKKPIESKKDTKGMILDVA

PRKLYNSEKPGEKTPEVKRLSKLVMDLKVDTLSLEERKEPVNNETLVKTEQEKNSRKSNSEPQSAGFFSK

QWMNNDDNL**P**L**PPPP**TL**P**ISRSARDGSRSSTPTQNILNKLTSMFAIDSGSKSDRPISRSNGDSAQVSNDS

ENEAESLLKHKRQINNL**KKK**L**KK**QVDYTDEQDLIIDEQEKEIKRLLEENAKLCMEKVQVENEIEDLSKSL

FTEANELVKNEAILRNKAEKLVEELRSEVEQLKAQLAKYFNQRPIELSKDEAHENTHLSSFPSSMVMAFI

PRFHSPDFWLARVFDLFGVNKQEWKYIPGQTKFTTWSEVLMGTTIYLLIVFGGKAVMKNQKPFELKTVWR

FHNLTLTVMSLLLLILFAEQLIPNLLNHSLFWNICHRNAWNAKLELLYYINYLIKWYEFIDTFLLVLKKK

NTPFLHVYHHSMTMILCFTQLQGMTSVSWVPVTINLFVHVIMYYYYYLTALGIRVWWKKLVTTMQIVQFI

IDLGFVYFCTYIYYAHKYRLAFSFKESCNGTEFAAIFGCLLLSSYLLLFVQFFIDTYNQKRKDRKAKAKA

RSAKKHTSQPKN

>XP_009494980.1 hypothetical protein H696_02805 [Fonticula alba]

MMSAPPDSAWVQSWLASSAAALPSHACGPCASIARAHALATCCSQLPISLDSDTVAAQSATAAAAAADNS

KAEAESCGRCGAACHALPPFVSLELFLEAVLPCLTPDTSPSDTLAILALLHMVGHLEFYTEHHLWDRIAH

CFWFADPSRHRAQILVHATSHFYDSPSCWAGAREYFPLPERSPLLFDAATVCYLFDLLEADQTSEETALS

VIILLACPPFRPPLLLQMLSHPHAETDTRSPYLPLFQLLLFSYFDRNPDEEARVADTSGPNPILNLLADR

VPQRSCVFSEQLAMYYSRGTDGDIHQGGDPLFSLMVARFAGALHADPASDPAARRTCNFFYPADLRVVID

CALRELHNLPADEPTLRSLRRAHLTFLANVLAREEVYRATGDHSRARIEEYVRGVASVAGPGGPVDRRFA

ALAGRVSELLGGIDPSHIT

>XP_014156322.1 hypothetical protein SARC_05291 [Sphaeroforma arctica JP610]

MSLLEKDPHRKYPQLTPVGREEPVKLRQL**PFEKGSVLTILKKVSDDWYFGSNASGEKGLIPVKFVSLSHK**

**SQLA**LQKIKSVSSLSRLSSMPSGTLSPDPASLSRLQSQNSIPKLAPAPPIQNATVNIDEFAMKAREASGA

SYTECVAAVKSVLDTLESKLPPDMKGRIHIGDTLNFAPSSRRKSSVNVDMDEFKSLLKELYNHMRDAQNV

NWSTADDSDWLTGKLEEMQELLSDADTKICRRAISFQNYEYTDTLLTYYQMDPRESVRKTLLITYGAMGS

VDRQVFSHGLVMSLPTELARSIDYDCVPSEFLFYQLVFCTMLFSTGEALPRQCYDHWTVELLLKLLELVE

DVNDSALSSHVSEAAFNVLLAFNLHQSAGDTIKADGVHSNQIVMDAVNRTDGCQLFSERLLLLVNRNEDP

VVLPITPGDNHTDVPNSLLHMLRCLFGCRATASIFFTNDLRVLVDIILREMENSGPDSSSLQLWLDILLR

IALNTDTGTEYRIADLRICLSDLLGHTLPEKMKATATTILEVLPVE

>XP_004364249.2 hypothetical protein CAOG_03410 [Capsaspora owczarzaki ATCC 30864]

MQLVLALKDSAPAAQLAPGFVTFRKNDLMAVIGEIPAGATGSTALTVRTVLGQTGQILMQDVRVHDPSQD

AEDDDDDAAPPMLEPVARPSASNLAVAAVPVAPARSPKPQAASEAAAEEVVIHKNLIHLVRAHTGLSFEK

SRTAVEAVLFYLQNADASLAGPLTAAVKLPSDLRPNTRDQDRLSSIFAALLAAKNDSQQRNWRSHDDASD

LAENLTEMTSILMDTDPDLARAMLSQDDYEIVHGLVDYYMMETRTPLRILVLKSLAVMIKAHERVVSILL

SSLLPKELARDILAETIPEIEWLLQAVKVCTMVYSTGEQCPIANYDFWNIEFILHIVNKIENGAHLGFTE

EMVDSLIALLLSFNLHFSIDSPSLSAPHRDNIVMKALEQQSSAACLGERVLYMFNRNGRMHSLEKFLKDL

FTANFFYSNDLNVLMDIIIRQLENLEQLEERRFESLDILYRLMFVTDYSSTRYRVNDLRTCLTRLGREEA

NGDAAVAAFSILATKLRDVFCGEE

>XP_004997312.1 uncharacterized protein PTSG_01339 [Salpingoeca rosetta]

MEQQQQQQQQPQSKQQQRGPRSSVMI**ALHDFQPQQSGQLPLVQGRMYTLHMSHKDGWAHVTDTHNQSGLAPAAY**FKEAALVAAENLPCFVGQASIDVLSSILKKKNVGTYCLRERRRKRGTLCLLFKTKGKKVEELEIRT

CTKGFALAGDTVPRKTIQELLDFYLAHQVKDGWGKLKRPLTNKDLQKALEKQTGRSPRTSSPKRTAKAAA

AATVAQ**P**Q**P**QQ**PP**TTTTATATSTFSDEERQCLQAHVHHSLDFIAKLPKSFQYSDQQKQVIATLKEVEAKM

QTNQLIPADVLSFISDLDVSRVFKRASQSSKLGTTHKPATTASAGVPSRPSRPAPPPPQRKGSLATRPAP

PPPSSASAATASATAAESAALTTTPPQAPTSPHHNRKPAGAVVRQGSPLKQTVQVSPPPSPTQPVPAPVL

SVVAPVATTAPSAELPPPLPTPFADVENVLRNLKSEVIQQIRDENDISYNQSRGALLSVLDILWSHVDDA

QVLSRVGGVMAAPDSPIVGGQDEITLTELFERISGYAEDAQQRGWAIDDDTAEIHQCLHQFRRLVDDANP

TVVMRLVRQWLTPGRTGQYEIVEILSVFYQMETRVDLRLEMLLLLIALIKLDMDVIPILQLTVLPSELAR

DIMDSSTTDKDLQPKIQASLELFALIYSQGHNALSSQNAAYLQETFLPHLLSMSCGAVATQAEQAICDAA

IVALLALNKQVVKPATGSHALVKAISEHDVRAISHKLLMFMNRGEDPVAALLGVEPDCELQPAEHSVMKL

LQDVFSDASSGDRFAYEADLLLLVDIVLRELRDREITDPGLYQFLRLLHQIVSNTNGWKSMYGQVKDGVG

SLSAKEHVPYSVKPQLEKISACLEAKQ

>NP_001285000.1 uncharacterized protein Dmel_CG2258, isoform D [Drosophila melanogaster]

**MLKALYDFQAVYPKTISFDEGEYFILYQTSARQRNWWQVVSMKGNIGFVPSNYVM**KIKVEHDFLISFLNS

SIESLEKCTDHEINGIMSKDELLDRLREKKHTMERLYAESSERDGDSSLSYSHSYSDKGSHRHSHPHPTQ

SQTQLHSQHRQHS**PPP**SGGSVGSQRLDMSKKSMSSPAVGSSCGLPQNQGMIESPSMGSMQFQGSSCTIQTPQQQQPL**P**A**PP**A**P**A**P**S**P**SAASATPTAASTSKAATGGDVAQDNSITSEPSETTTTTTTTSEDVVTTYKETS

QMSTSQQHKPQNGSTASASISASASASALRSNGGNGKANGSATALHQELQQEEADSAPDKSDDASAVQSD

DGCPANGDSADNSQALDSIDSPSHRQRGLSLGRIEEGATGGGQLKVESSDVYQIVDALRRNTNLSFDLSC

EALRVVLTSLEQLYNGAINPYLEAVAVHVTGKVATPKELLGITHDAKRLQYLFAQLADCKNDTEQRTWML

YEDEEDIIQFLEELVEILINADESISCYEMSCDQYQMFINLVQYYQMETRWSIKRLLLKTFTAACHLDYI

IVDILLTSVLPLEIVEDMKTHFSNLDRFKQLVKMLTIIFSLGQPMPVNHQDYLGVHFASFLLEIVEGNNP

EVLVDMVIALILAFNQQFSEHTYNVIIEGMQNLPSAKVFTEKLLLLLNREDDPTRLLKHPNEHMNTVLRM

FIDIFSHPDTAGMFYTNDIKVLIDIVVRQLSDLDAGSTTRPCYLELCRRILRNTNYQEHQHRKHDLMKIF

TRIFCEETECSASDQQLVREIANEFPQLFKA

>XP_026695884.1 NCK-interacting protein with SH3 domain-like [Ciona intestinalis]

M**FAVAKDFVARDGEPHTMSVCKGEKVCVLEKTNEAWWLVANQNGGVGFVPTSYM**DGTVTSSNDVIIKSIDEAISKIYISSKGKLSADQESLLFKLNEHKDEVS**KKRKRK**A**PP**V**P**CRAVNQTSQPIREQEESSGPIRGQEE

SSRPIRGQEESSRPIRGQEESSRPIRGQKESSGPIRGQEESSGPIRGQKESSGPIRGQEESSRPIRGQEE

SSRPIRGQEESSGPIRGQKESSGPIRGQKESSGPIRGQEESSGPIRGQEESSGPIRGQKESSGPIRGQEI

PENLGEDLLHLVTSTTNITKRESKEIVVSLLSLIANQVPTLDGAMHNIISTLPMLAAEPDDEVAKLGLAF

ANLCEIKNDAQQRNWAVQDDENDIVEILNQILTLTSTLDTKYIPLLRHNDNNALMTIANYYQMEPKVRIR

LILLQIIGAICSISPKFISVFVTSVLPEELVREVTDHARSDKMRVAYCCVVLSLLFSTGEPVPVAYDETI

GESFVKYLMDIIEDGTVGEDGSHEDLDDVMIRVILSINLHHVSPDANPTLNVIAARGGSTRLSEKLMLLF

NRGHDPVNEGCDPVNEGHDPTSCDQPPNSVLKFFQDIFARRDTGDCFLYTNDLMVLIDIIVRNIADLSSG

SKGRTEYLDLLLKVAGTSSFIETRHRCRDLFQILNRIKNEELPHDDSFRKLNNAESQ

>NP_109654.2 NCK-interacting protein with SH3 domain [Mus musculus]

**MYRALYAFRSAEPNAMAFAAGETFLVLERSSTHWWLAARARSGETGYVPPAYL**HRLQGMEQDVLQAIDRA

IEAVHNTAMRDGGKYSLEQRGVLQKLIHHRKETLSRRGTSASSATVMTPSTSDHHLDAAVSRQPNGVCRT

GFERQHSLPSSEHLGTDGALYQV**PP**Q**PRR**AA**P**TT**PPPP**V**KRR**DREALVISGSGGRTAIPSGGSSVSSGSS

ASSTSMDTLYTGSSPSELG**P**SCS**P**T**PPP**V**PRR**GAHTTVSQPQPSPSKAPSPEPPTEEVAAETNSTPDDLE

AQDALSPETTEEKAAAETVVPRTIGAELMELVRRNTGLSHELCRVAIGVVVGHIQATVPASSPIMEQVLL

SLVEGKDLSTALPSGQVCHDQQRLEVIFADLARRKDDAQQRSWALYEDEDVIRCYLEELLHILTDADPEV

CKKMCKRSDFESVLALVAYYQMEHRASLRLLLLKCFGAMCSLDAAIISTLVSSVLPVELARDMQTDTQDH

QKLCYSALVLAMVFSMGEAVPYAHYEHLGTPFAQFLLSIVEDGLPMDTTEQLPDLCMNLLLALNLHLTAP

EQNVIMAALSRHTNVKIFSEKLLLLLNRGDDPVRIFRHEPQPPHSVLKFLQDVFSSSATAAIFYHTDMMA

LIDITVRQIADLSPGDKLRMEYLSLMHAVVRSTPYLQHRHRLSDLQATLRRILTEEEASPQCQMDRMIVQ

EMYKEFPDLGEVPS

>XP_013754362.1 hypothetical protein AMSG_09524 [Thecamonas trahens ATCC 50062]

MADGEYEYYYDEYEVTVGDEEAVKKALVAAVEGVASLEGMTLEALRAAVEAGSRVAEIKVAVEAEREAHR

KKMAALEAELVEEEKRAAALEAVAPLLKQLKPAPAAAGDGGPARAFSQAHLSKMYRMLGPAGDADHVVPV

KMLEFVTADGYANVGKLATQYVAETAVPFRTAEAKILARLVETFPETAERLVACKGLVARAMVSLTAPAA

PASMAFAEASLVGLLLHQETQLPVEVVATAFSPQVLDALLLAVVAHETDPDETLLDALLRLVLLVNVAAA

GDEGPGKLSLVVQALLRASDVKAVVEYVPRLFNGGPTGYHSMVAGEPVLKMLLDIFASPASLSLLYPNDV

HVVLNVIVRELNNLDPESPKRMYMLRILEAYFSNSDYGSGAEEPYKIAEFQTTLDSIHSEDGEASEMSRL

MVEAIYMSCEVLATWRPPAP

>XP_629323.1 hypothetical protein DDB_G0293098 [Dictyostelium discoideum AX4]

MSETDIIETINQLVSKEFLDINDINELIDQIKQLNFDTALNQISNNNYHLIKLLCNEAKKSIDSNLVTEI

IKFINVIANEDKNIVSILIEMESFNWIIPNCFIVEETLSINYLTTLNLIFSNFDNLPKKELLKLDDSFLD

SLIELALQFKDYYETNFIHSLYYTMFGYQKNIISDGYSPLILKLYSIKEAPEIGPEMISLLNRSNLEYNM

LPQCLSLINDLFTYSTEHEIPCFFFTLDIKVLIDIIIREINNLDELDPSRWQFLEVLSTIIDHSEYSSPN

FNNDSKDNNNNQNNNYNDEEIISFYKIDDIKNVLSILNEDRNSEKDPQSSILSKSILKKLNKLQQQKKR

>PRP84337.1 hypothetical protein PROFUN_07638 [Planoprotostelium fungivorum]

MAEICSQIDWNDVDSPEITNNLEMIFQSLMNGTLGEELLDDNMIFVQKLSQLLQTCIQSPQQQKRSNLVM

ALSTAVVMGDRFRGIVFDRWLSRKALESWLIPCIRLRDDPEVPFYALSLLNMILDTPIHEKRFEPLNHEL

VCYLLDLTVDIEYDLDQHNLFLEGMKSLAGIYDTLDEMAASQLRSKTRGRDVILGNARAKANAGEDNPVI

YQIHHHPSAVEFGQNFIKLLNRGMDSRTTRRTLKLMSEILRDDKLSMSFFYWNDLKVLIDILMRDFDNFD

EGAENDELRLLCLRVLGLVLKNTEYSTQEVPHRPEDIRQFLKRISESDMSESITIEAQEIFSSF

>XP_020433395.1 hypothetical protein PPL_06118 [Heterostelium album PN500]

MDTSTLVKEFNSLIRKAKESERDNIQISLNFYKQAYKLLPNAPNLSKKIESLERKLVKIQAELLKEDWVQ

IEGNSIDIKTSGGGTSNAPITSSPTIFNDSNTSDGIDDFSIKQEDEDDDDDEQNNNNNNSNNMGEEDLDD

FIKVDATSIEDIDTLIDKLKLMDIDVALKLLQQNDFFVIRSMCKHAQQSEDVNYISEIVYFLNGIGNQVE

GVFSLLVSELAWIIPNCFMVEETKAAQHLLLLAAIFTQTGPLPATELAKLDETFLDELIDLGFKYLDYYD

TKWLNIFYNTLLAYQSQIIVNDGYSPLIHKLYTMQKAPELGPELIALLNRDKFDYGLIGQSLSLVSDIFS

YSTEHKVSCFFYSSDIPIIIDILVRNIHNLNELDPLRWQYLDVLYIVLSHEHFQQTQYHVDDIITVLTDM

LDEDYAEKDPRTALVSRSILDLIQQQSYQ
