# Supplementary material for: Evolutionarily diverged on-switch for actin assembly in fungal endocytosis
Source: J Cell Biol. 2026 Oct 28;226(1):e202506135. doi: 10.1083/jcb.202506135 (PMC13715591; doi:10.1083/jcb.202506135)
Supplement: Supplementary file 3 [file jcb_202506135_tables1.docx]

**Table S1:** **Yeast strains**

| Strain number | Genotype | Source |
| --- | --- | --- |
| MKY0100 | *MATa his3-∆200 leu2-3,112 ura3-52 lys2-801* | This study |
| MKY0102 | *MATα his3-∆200 leu2-3,112 ura3-52 lys2-801* | This study |
| MKY3861 | *MATα his3-∆200 leu2-3,112 ura3-52 lys2-801 sla1∆::URA3* | This study |
| MKY4014 | *MATα his3-∆200 leu2-3,112 ura3-52 lys2-801 SLA1-EGFP::HIS3MX6 SAC6-mCherry::kanMX4* | This study |
| MKY4017 | *MATa his3-∆200 leu2-3,112 ura3-52 lys2-801 PAN1-EGFP::HIS3MX6 SAC6-mCherry::kanMX4* | This study |
| MKY4116 | *MATa his3-∆200 leu2-3,112 ura3-52 lys2-801 abp1∆::URA3* | This study |
| MKY4117 | *MATα his3-∆200 leu2-3,112 ura3-52 lys2-801 abp1∆::URA3* | This study |
| MKY4478 | *MATα his3-∆200 leu2-3,112 ura3-52 lys2-801 LDB17-mNeonGreen::HIS3MX6* | This study |
| MKY4504 | *MATα his3-∆200 leu2-3,112 ura3-52 lys2-801 LDB17-mNeonGreen::HIS3MX6 SAC6-mCherry::kanMX4* | This study |
| MKY4505 | *MATa his3-∆200 leu2-3,112 ura3-52 lys2-801 LDB17-mNeonGreen::HIS3MX6 SAC6-mCherry::kanMX4* | This study |
| MKY4508 | *MATα his3-∆200 leu2-3,112 ura3-52, lys2-801 LDB17-mNeonGreen::HIS3MX6 bzz1-(w531a)::URA3* | This study |
| MKY4509 | *MATa his3-∆200 leu2-3,112 ura3-52 lys2-801 LDB17-mNeonGreen::HIS3MX6 bzz1-(w531a)::URA3* | This study |
| MKY4510 | *MATα his3-∆200 leu2-3,112 ura3-52 lys2-801 LDB17-mNeonGreen::HIS3MX6 bzz1-(1-576)::natNT2* | This study |
| MKY4511 | *MATa his3-∆200 leu2-3,112 ura3-52 lys2-801 LDB17-mNeonGreen::HIS3MX6 bzz1-(1-576)::natNT2* | This study |
| MKY4512 | *MATα his3-∆200 leu2-3,112 ura3-52 lys2-801 LDB17-mNeonGreen::HIS3MX6 bzz1∆::natNT2* | This study |
| MKY4513 | *MATa his3-∆200 leu2-3,112 ura3-52 lys2-801 LDB17-mNeonGreen::HIS3MX6 bzz1∆::natNT2* | This study |
| MKY4520 | *MATα his3-∆200 leu2-3,112 ura3-52 lys2-801 LDB17-mNeonGreen::HIS3MX6 sla1∆::natNT2* | This study |
| MKY4521 | *MATa his3-∆200 leu2-3,112 ura3-52 lys2-801 LDB17-mNeonGreen::HIS3MX6 sla1∆::natNT2* | This study |
| MKY4522 | *MATa his3-∆200 leu2-3,112 ura3-52 lys2-801 LDB17-mNeonGreen::HIS3MX6* | This study |
| MKY4523 | *MATα his3-∆200 leu2-3,112 ura3-52 lys2-801 SLA1-EGFP::HIS3MX6 SAC6-mCherry::kanMX4 ldb17∆::natNT2* | This study |
| MKY4524 | *MATa his3-∆200 leu2-3,112 ura3-52 lys2-801 SLA1-EGFP::HIS3MX6 SAC6-mCherry::kanMX4 ldb17∆::natNT2* | This study |
| MKY4529 | *MATα his3-∆200 leu2-3,112 ura3-52 lys2-801 ldb17∆::natNT2* | This study |
| MKY4530 | *MATa his3-∆200 leu2-3,112 ura3-52 lys2-801 ldb17∆::natNT2* | This study |
| MKY4560 | *MATα his3-∆200 leu2-3,112 ura3-52 lys2-801 ldb17-(1-432)-mNeonGreen::HIS3MX6* | This study |
| MKY4561 | *MATa his3-∆200 leu2-3,112 ura3-52 lys2-801 ldb17-(1-432)-mNeonGreen::HIS3MX6* | This study |
| MKY4563 | *MATα his3-∆200 leu2-3,112 ura3-52 lys2-801 sla1-(1-855)::natNT2 LDB17-mNeonGreen::HIS3MX6* | This study |
| MKY4564 | *MATa his3-∆200 leu2-3,112 ura3-52 lys2-801 sla1-(1-855)::natNT2 LDB17-mNeonGreen::HIS3MX6* | This study |
| MKY4565 | *MATα his3-∆200 leu2-3,112 ura3-52 lys2-801 vrp1∆::hphNT1* | This study |
| MKY4566 | *MATa his3-∆200 leu2-3,112 ura3-52 lys2-801 vrp1∆::hphNT1* | This study |
| MKY4570 | *MATα his3-∆200 leu2-3,112 ura3-52 lys2-801 lsb3∆::URA3 LDB17-mNeonGreen::HIS3MX6* | This study |
| MKY4571 | *MATa his3-∆200 leu2-3,112 ura3-52 lys2-801 lsb3∆::URA3 Ldb17-mNeonGreen::HIS3MX6* | This study |
| MKY4572 | *MATα his3-∆200 leu2-3,112 ura3-52 lys2-801 lsb4∆::natNT2 LDB17-mNeonGreen::HIS3MX6* | This study |
| MKY4573 | *MATa his3-∆200 leu2-3,112 ura3-52 lys2-801 lsb4∆::natNT2 LDB17-mNeonGreen::HIS3MX6* | This study |
| MKY4592 | *MATα his3-∆200 leu2-3,112 ura3-52 lys2-801 vrp1∆::hphNT1 ldb17∆::natNT2* | This study |
| MKY4601 | *MATα his3-∆200 leu2-3,112 ura3-52 lys2-801 PAN1-EGFP::HIS3MX6 SAC6-mCherry::kanMX4 ldb17∆::natNT2* | This study |
| MKY4602 | *MATa his3-∆200 leu2-3,112 ura3-52 lys2-801 PAN1-EGFP::HIS3MX6 SAC6-mCherry::kanMX4 ldb17∆::natNT2* | This study |
| MKY4603 | *MATα his3-∆200 leu2-3,112 ura3-52 lys2-801 PAN1-EGFP::HIS3MX6 SAC6-mCherry::kanMX4 sla1∆::URA3* | This study |
| MKY4604 | *MATa his3-∆200 leu2-3,112 ura3-52 lys2-801 PAN1-EGFP::HIS3MX6 SAC6-mCherry::kanMX4 sla1∆::URA3* | This study |
| MKY4610 | *MATα his3-∆200 leu2-3,112 ura3-52 lys2-801 LDB17-mNeonGreen::HIS3MX6 PIL1-mCherry::kanMX4* | This study |
| MKY4611 | *MATa his3-∆200 leu2-3,112 ura3-52 lys2-801 LDB17-mNeonGreen::HIS3MX6 PIL1-mCherry::kanMX4* | This study |
| MKY4614 | *MATα his3-∆200 leu2-3,112 ura3-52 lys2-801 PAN1-EGFP::HIS3MX6 SAC6-mCherry::kanMX4 ldb17∆::natNT2 sla1∆::URA3* | This study |
| MKY4615 | *MATa his3-∆200 leu2-3,112 ura3-52 lys2-801 PAN1-EGFP::HIS3MX6 SAC6-mCherry::kanMX4 ldb17∆::natNT2 sla1∆::URA3* | This study |
| MKY4624 | *MATa his3-∆200 leu2-3,112 ura3-52 sla1∆::cgLEU2 sla1-W41AW108AW391A::URA3* | Sun et al, 2017 |
| MKY4626 | *MATa his3-∆200 leu2-3,112 ura3-52 sla1-W391A::URA sla1∆::natNT2* | Sun et al, 2017 |
| MKY4636 | *MATα his3-∆200 leu2-3,112 ura3-52 lys2-801 LAS17-EGFP::HIS3MX6 SAC6-mCherry::kanMX4 ldb17∆::natNT2* | This study |
| MKY4637 | *MATa his3-∆200 leu2-3,112 ura3-52 lys2-801 LAS17-EGFP::HIS3MX6 SAC6-mCherry::kanMX4 ldb17∆::natNT2* | This study |
| MKY4639 | *MATα his3-∆200 leu2-3,112 ura3-52 lys2-801 SAC6-mCherry::kanMX4 PAN1-EGFP::HIS3MX6 ldb17-(1-432)::HIS3* | This study |
| MKY4640 | *MATa his3-∆200 leu2-3,112 ura3-52 lys2-801 SAC6-mCherry::kanMX4 PAN1-EGFP::HIS3MX6 ldb17(1-432)::HIS3* | This study |
| MKY4641 | *MATa his3-∆200 leu2-3,112 ura3-52 lys2-801 LDB17-mNeonGreen::HIS3MX6 sla1∆::cgLEU2 sla1-W41AW108AW391A::URA3* | This study |
| MKY4642 | *MATα his3-∆200 leu2-3,112 ura3-52 lys2-801 LDB17-mNeonGreen::HIS3MX6 sla1-W391A::URA3 sla1∆::natNT2* | This study |
| MKY4643 | *MATa his3-∆200 leu2-3,112 ura3-52 lys2-801 LDB17-mNeonGreen::HIS3MX6 sla1-W391A::URA3 sla1∆::natNT2* | This study |
| MKY4653 | *MATα his3-∆200 leu2-3,112 ura3-52 lys2-801 LDB17-mNeonGreen::HIS3MX6 lsb3∆::URA3 lsb4∆::natNT2* | This study |
| MKY4654 | *MATa his3-∆200 leu2-3,112 ura3-52 lys2-801 LDB17-mNeonGreen::HIS3MX6 lsb3∆::URA3 lsb4∆::natNT2* | This study |
| MKY4655 | *MATα his3-∆200 leu2-3,112 ura3-52 lys2-801 ldb17∆::natNT2 sla1∆::URA3* | This study |
| MKY4657 | *MATα his3-∆200 leu2-3,112 ura3-52 lys2-801 ldb17-(1-432)-mNeonGreen::HIS3MX6 PIL1-mCherry::kanMX4* | This study |
| MKY4658 | *MATa, his3-∆200 leu2-3,112 ura3-52 lys2-801 ldb17-(1-432)-mNeonGreen::HIS3MX6 PIL1-mCherry::kanMX4* | This study |
| MKY4659 | *MATα his3-∆200 leu2-3,112 ura3-52 lys2-801 ldb17∆::natNT2 abp1∆::URA3* | This study |
| MKY4660 | *MATa his3-∆200 leu2-3,112 ura3-52 lys2-801 ldb17∆::natNT2 abp1∆::URA3* | This study |
| MKY4661 | *MATα his3-∆200 leu2-3,112 ura3-52 lys2-801 LDB17-mNeonGreen::HIS3MX6 bzz1∆::natNT2 sla1∆::natNT2* | This study |
| MKY4662 | *MATa his3-∆200 leu2-3,112 ura3-52 lys2-801 LDB17-mNeonGreen::HIS3MX6 bzz1∆::natNT2 sla1∆::natNT2* | This study |
| MKY4673 | *MATα his3-∆200 leu2-3,112 ura3-52 lys2-801 SAC6-mCherry::kanMX4 PAN1-EGFP::HIS3MX6 ldb17-(1-432)::HIS3* | This study |
| MKY4674 | *MATa his3-∆200 leu2-3,112 ura3-52 lys2-801 SAC6-mCherry::kanMX4 PAN1-EGFP::HIS3MX6 ldb17-(1-432)::HIS3* | This study |
| MKY4801 | *MATα his3-∆200 leu2-3,112 ura3-52 lys2-801 sla1∆::URA3 LAS17-EGFP::HISMX6 SAC6-mCherry::kanMX4* | This study |
| MKY4802 | *MATa his3-∆200 leu2-3,112 ura3-52 lys2-801 sla1∆::URA3 LAS17-EGFP::HISMX6 SAC6-mCherry::KANMX4* | This study |
| MKY4888 | *MATα his3-∆200 leu2-3,112 ura3-52 lys2-801 LDB17-mNeonGreen::HIS3MX6 sla1-(W41A)::natNT2* | This study |
| MKY4889 | *MATa his3-∆200 leu2-3,112 ura3-52 lys2-801 LDB17-mNeonGreen::HIS3MX6 sla1-(W41A)::natNT2* | This study |
| MKY4890 | *MATα his3-∆200 leu2-3,112 ura3-52 lys2-801 LDB17-mNeonGreen::HIS3MX6 sla1-(W108A)::natNT2* | This study |
| MKY4891 | *MATa his3-∆200 leu2-3,112 ura3-52 lys2-801 LDB17-mNeonGreen::HIS3MX6 sla1-(W108A)::natNT2* | This study |
| MKY4892 | *MATα his3-∆200 leu2-3,112 ura3-52 lys2-801 LDB17-mNeonGreen::HIS3MX6 sla1-(W41AW108A)::natNT2* | This study |
| MKY4893 | *MATa his3-∆200 leu2-3,112 ura3-52 lys2-801 LDB17-mNeonGreen::HIS3MX6 sla1-(W41AW108A)::natNT2* | This study |
| MKY4911 | *MATα his3-∆200 leu2-3,112 ura3-52 lys2-801 sla1∆::URA3 LDB17del::natNT2 LAS17-EGFP::HISMX6 SAC6-mCherry::kanMX4* | This study |
| MKY4912 | *MATa his3-∆200 leu2-3,112 ura3-52 lys2-801 sla1∆::URA3 ldb17∆::natNT2 LAS17-EGFP::HISMX6 SAC6-mCherry::kanMX4* | This study |
| MKY4988 | *his3-∆200 leu2-3,112 ura3-52 lys2-801 PAN1-EGFP::HIS3MX6 SAC6-mCherry::kanMX4 sla1-(W41AW108A)::natNT2* | This study |
| MKY4990 | *MATa his3-∆200 leu2-3,112 ura3-52 lys2-801 sla1-(W41AW108A)::natNT2 ldb17(1-432)-mNeonGreen::HIS3MX6* | This study |
| MKY4991 | *MATα his3-∆200 leu2-3,112 ura3-52 lys2-801 sla1-(W41AW108A)::natNT2 ldb17(1-432)-mNeonGreen::HIS3MX6* | This study |
| MKY5000 | *MATa his3-∆200 leu2-3,112 ura3-52 lys2-801 sla1-(W41AW108A)::natNT2 SAC6-mCherry::kanMX4 PAN1-EGFP::HIS3MX6 ldb17(1-432)-mNeonGreen::HIS3MXM6* | This study |
| MKY5848 | *MATα his3-∆200 leu2-3,112 ura3-52 lys2-801 SLA1-EGFP::HIS3MX6 SAC6-mCherry::KANMX4 ldb17-D323K::URA3* | This study |
| MKY5897 | *MATα his3-∆200 leu2-3,112 ura3-52 lys2-801*  *ldb17-D323K-mNeonGreen::HIS3 SAC6-mCherry::KANMX4* | This study |
| MKUM0028 | *a1 b1 DIP1-mNeonGreen::kanMX6 FIM1-mCherry::natMX6* | This study |
| MKYP0022 | *h- SHD1-sfGFP::kanMX6 FIM1-mCherry::natMX6* | This study |
| MKYP0096 | *h+ DIP1-mNeonGreen::kanMX6* | This study |
| MKYP0097 | *h+ DIP1-mNeonGreen::kanMX6 FIM1-mCherry::natMX6* | This study |
| MKYP0107 | *h- dip1∆::hphMX6 SHD1-sfGFP::kanMX6 FIM1-mCherry::natMX6* | This study |
| MKYP0110 | *h+ shd1∆::hphMX6 DIP1-mNeonGreen::kanMX6* | This study |
