# Supplementary material for: Evolutionarily diverged on-switch for actin assembly in fungal endocytosis
Source: J Cell Biol. 2026 Oct 28;226(1):e202506135. doi: 10.1083/jcb.202506135 (PMC13715591; doi:10.1083/jcb.202506135)
Supplement: Supplementary file 4 [file jcb_202506135_tables2.docx]

**Table S2: Plasmids**

| Number | Name | Tag | Selection | Source | Function |
| --- | --- | --- | --- | --- | --- |
| pMK0333 | pFA6a-mNeonGreen-HIS3MX6 | mNeonGreen | HIS3MX6 | This study | tagging plasmid |
| pMK0019 | pFA6a-natNT2 |  | natNT2 | Janke et al. 2004 | deletion plasmid |
| pMK0271 | pFA6-hphMX6 |  | HphMX6 | Wu et al. 2003 | deletion plasmid |
| pMK0436 | pMK0271-5dip1-Hhp-3'dip1 |  | HphMX6 | This study | deletion plasmid |
| pMK0086 | pFA6a-KiUra |  | KlURA3 | This study | deletion plasmid |
| pMK0045 | pYM-N17 (GPD-Promotor) |  | natNT2 | Janke et al. 2004 | mutagenesis plasmid |
| pMK0411 | pCoofy6-Ldb17 |  | KlURA3 | This study | mutagenesis plasmid |

Plasmids used for gene tagging and deletion.
