# Supplementary material for: Evolutionarily diverged on-switch for actin assembly in fungal endocytosis
Source: J Cell Biol. 2026 Oct 28;226(1):e202506135. doi: 10.1083/jcb.202506135 (PMC13715591; doi:10.1083/jcb.202506135)
Supplement: Supplementary file 5 [file jcb_202506135_tables3.docx]

**Table S3: Yeast media and buffers**

| **Media** | **Species** | **Components (w/v)** |
| --- | --- | --- |
| YPD | *S. cerevisiae* | 1% Yeast extract, 2% Bacto peptone, 2% Glucose, 40 mg/L uracil & adenin |
| YES | *S. pombe* | 0.5% Yeast extract, 3% Glucose, 0.0225% Adenine, 0.0225% Histidine, 0.0225% Leucine, 0.0225% Lysine, 0.0225% Uracil |
| YEPS-light | *U. maydis* |  |
| Sc-Trp | *S. cerevisiae* | 0.2% Sc-Trp, 0.67% yeast nitrogen base, 2% glucose |
| EMM | *S. pombe*  *U. maydis* | 0.3% potassium phthalate monobasic, 0.22% sodium phosphate dibasic, 0.5% ammonium chloride, 2% anhydrous glucose, 2% salts, 1% vitamins, 0.1% minerals |
| PEG | *S. cerevisiae* | 100nM LiAc, 10 mM Tris-HCl pH8, 1 mM EDTA pH8, 10% PEG |
| SORB | *S. cerevisiae* | 100nM LiAc, 10 mM Tris-HCl pH8, 1 mM EDTA pH8, 1 M Sorbitol |

Media used in this study. For plates 2% agar was added to these media.
