# Supplementary material for: Evolutionarily diverged on-switch for actin assembly in fungal endocytosis
Source: J Cell Biol. 2026 Oct 28;226(1):e202506135. doi: 10.1083/jcb.202506135 (PMC13715591; doi:10.1083/jcb.202506135)
Supplement: Supplementary file 6 [file jcb_202506135_tables4.docx]

**Table S4: Protein molecular masses**

| Protein | Fraction (%) | Mass (kDa) | σ (kDa) |
| --- | --- | --- | --- |
| Ldb17 | 93% | 62 | 11.1 |
|  | 3% | 123 | 12.7 |
| Ldb17-∆tail | 95% | 55 | 7.5 |
| Dip1 | 89% | 52 | 7.1 |
|  | 6% | 82 | 12.8 |

Measured molecular mass (Mw) and standard deviation (σ) of the detected oligomeric species of Ldb17, Ldb17-∆tail and Dip1 are shown. Data analysis (Gaussian fitting) was performed for each Gaussian fitted peak using the Dynamic MP software. For each fitted peak, the proportion of counts fitted to each Gaussian relative to the total number of counts is indicated.
